# Ligand-Mediated and Copper-Catalyzed C(sp^3^)-H Bond Functionalization of Aryl Ketones with Sodium Sulfinates under Mild Conditions

**DOI:** 10.1038/srep18391

**Published:** 2015-12-18

**Authors:** Xing-Wang Lan, Nai-Xing Wang, Cui-Bing Bai, Wei Zhang, Yalan Xing, Jia-Long Wen, Yan-Jing Wang, Yi-He Li

**Affiliations:** 1 Technical Institute of Physics and Chemistry, Chinese Academy of Sciences, Beijing, 100190, China; 2 Department of Chemistry, William Paterson University of New Jersey, 300 Pompton Road, Wayne, New Jersey 07470, United States; 3 Beijing Key Laboratory of Lignocellulosic Chemistry, Beijing Forestry University, Beijing, 100083, P. R. China

## Abstract

A novel and convenient copper (II) bromide and 1,8-diazabicyclo[5.4.1]undec-7-ene (DBU) or 1,10-phenanthroline catalysis protocol for the construction of *α*-alkyl-*β*-keto sulfones *via* C(sp^3^)-H bond functionalization followed by C(sp^3^)-S bond formation between aryl ketones and sodium sulfinates at room temperature has been developed. This method is applicable to a wide range of aryl ketones and sodium sulfinates. The electronic effects of aryl ketones and ligands effects of the copper salts are crucial for this transformation. Typically, substituted aryl ketones with electron-withdrawing group do not need any ligand to give a good to excellent yield, while substituted aryl ketones with electron-donating group and electron-rich heteroaromatic ketones offer a good to excellent yield only under the nitrogen-based ligands. The practical value of this transformation highlights the efficient and robust one-pot synthesis of *α*-alkyl-*β*-keto sulfones.

Since its initial discovery, transition-metal-catalyzed C-H bond functionalization has emerged as an efficient and powerful synthetic strategy in modern synthetic chemistry^1^,^2^,^3^,^4^,^5^, and there are extensive applications in academic research and industry. In particular, the formation of C-S bonds has been widely investigated and significant efforts have been devoted to the development of synthetic methodology on the compounds containing C-S bond scaffolds^6^,^7^,^8^. For example, Guo *et al.*^9^ have recently presented an approach on copper-catalyzed aerobic decarboxylative sulfonylation of alkenyl carboxylic acids with sodium sulfinates. Liu *et al.*^10^ have reported an unprecedented approach on the construction of 2-sulfonylbenzo[b]furans from readily available *trans*-2-hydroxycinnamic acids and sodium sulfinates mediated by copper and silver salts under mild conditions. Therefore, the development of efficient C-S bond formation still remains a challenge in recent decades.

*β*-Keto sulfones are of much significant intermediates owing to their ubiquitous presence and versatile applications in biological and pharmaceutical areas^11^,^12^, as well as in the synthesis of acetylenes^13^, olefins^14^, allenes^15^, vinyl sulfones^16^, and some natural products^17^. As a result of their synthetic value and versatile reactivity, great efforts have been made towards constructing *β*-keto sulfones during the past decades^18^,^19^,^20^,^21^,^22^,^23^,^24^. For instance, Ramaiah’s^23^ and Venkateswarlu’s^24^ groups have reported a traditional nucleophilic substitution reaction using the toxic *α*-halogenated aryl ketones and sodium sulfinates, respectively. However, up to now, rare examples have been reported on using C-H bond functionalization to synthesize *β*-keto sulfones. Recently, Lei *et al.*^25^ have reported an unprecedented protocol of achieving *β*-keto sulfones by using terminal alkynes and benzenesulfinic acid *via* a dioxygen-triggered radical process (Fig. 1(a)). The process involves C(sp)-H bond carbonylation and C(sp^3^)-H bond functionalization. Yadav *et al.*^26^ later described a AgNO_3_/K_2_S_2_O_8_ catalyzed radical route to the synthesis of *β*-keto sulfones *via* C(sp^2^)-H bond carbonylation and C(sp^3^)-H bond functionalization using dioxygen as oxidant (Fig. 1(b)). However, no report on the synthesis of *β*-keto sulfones *via* a formal C(sp^3^)-H bond functionalization coupling reaction has been presented so far, especially on the synthesis of *α*-alkyl-*β*-keto sulfones.

In this paper, we wish to report a simple and novel copper (II) bromide and 1,8-diazabicyclo[5.4.1]undec-7-ene (DBU) or 1,10-phenanthroline catalytic protocol involving the C(sp^3^)-H functionalization aryl ketones with sodium sulfinates for the synthesis of *α*-alkyl-*β*-keto sulfones under mild conditions (Fig. 1(c)). This reaction can be regarded as a dehydrogenative coupling involving a formal C(sp^3^)-H bond functionalization at the *α*-position of the aryl ketones. The nitrogen-based ligands play a key role in the electronic effects of aryl ketones. Comparing to previous works, this method can realize the transformation by the nitrogen-based ligand under simple operation and mild conditions. To the best of our knowledge, this is first report of the C(sp^3^)-H functionalization followed by C(sp^3^)-S bond formation of aryl ketones for the synthesis of *α*-alkyl-*β*-keto sulfones *via* copper/DBU catalytic system with and without nitrogen-based ligand.

## Results

We commenced our studies by using propiophenone (**1a**) and sodium benzenesulfinate **(2a)** as model substrates under various conditions, and the results are summarized in Table 1. As shown in Table 1, when the reaction was carried out in the presence of CuBr_2_ (20 mol%) in DMSO in an open flask (in air) for 24 h, no product was detected in the mixtures in spite of the *α*-C-H of a ketone possessing weak activity (entry 1), and the similar results were found after several bases being employed under the same conditions (entries 2–9). Gratifyingly, when the strong organic base DBU was examined, the desired product was generated in a 47% yield (entry 10). The structure of the product **3aa** was confirmed by comparison of ^1^H NMR, ^13^C NMR, and HRMS, even 2D NMR (see Supporting Information). To further improve the yield of **3aa**, we subsequently enhanced the amount of DBU, finding that the best yield (87%) was obtained in 5 hours when 1.0 equiv of DBU was added (entry 11), indicating that addition of the appropriate amount of DBU was crucial for the reaction. In these reactions, DBU has a dual functionality as a ligand and as a base to enolize the substrate. To further improve the yield, the 1.4 equiv of DBU was added, but the yield was not improved (entry 12). In addition, the blank experiments were also performed to verify the effect of the catalyst. As envisaged, no reaction occurred with DBU alone in the absence of CuBr_2_ (entry 13), indicating that CuBr_2_ as the catalyst plays a tremendously important role in this transformation. To further confirm our conclusion, several catalysts were also tested, but with disappointing results (entries 14–18). In addition, other solvents were also examined and were found to give lower yields (see Supporting Information), thus DMSO was considered as the most suitable solvent for the reaction.

**Figure 2:**
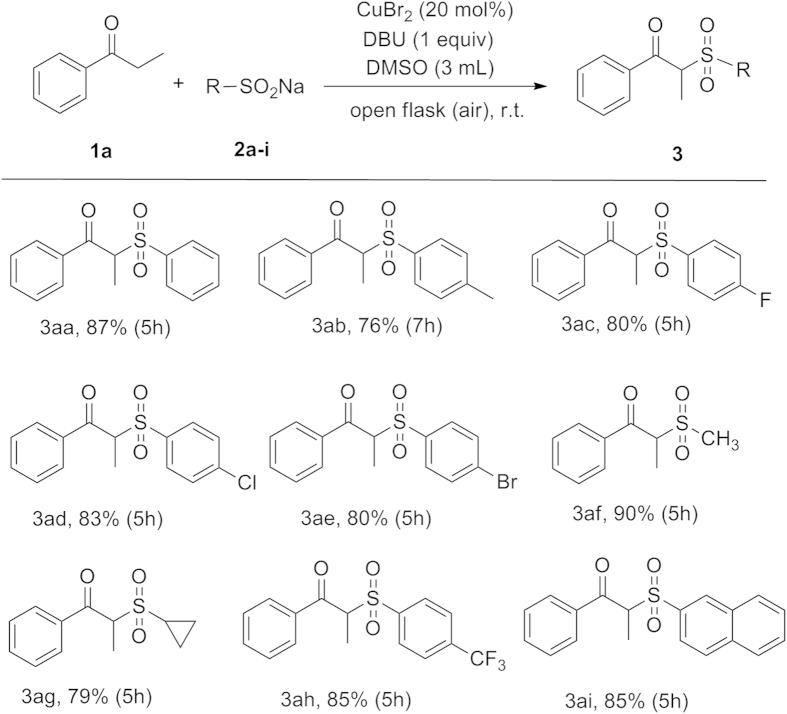
Scope of substrates 2. Reaction conditions: (**1a**) (0.5 mmol), (**2a–i**) (1.0 mmol), and CuBr_2_ (20 mol%) and DBU (1 equiv) in 3 mL DMSO at room temperature in an open flask (in air).

**Figure 3:**
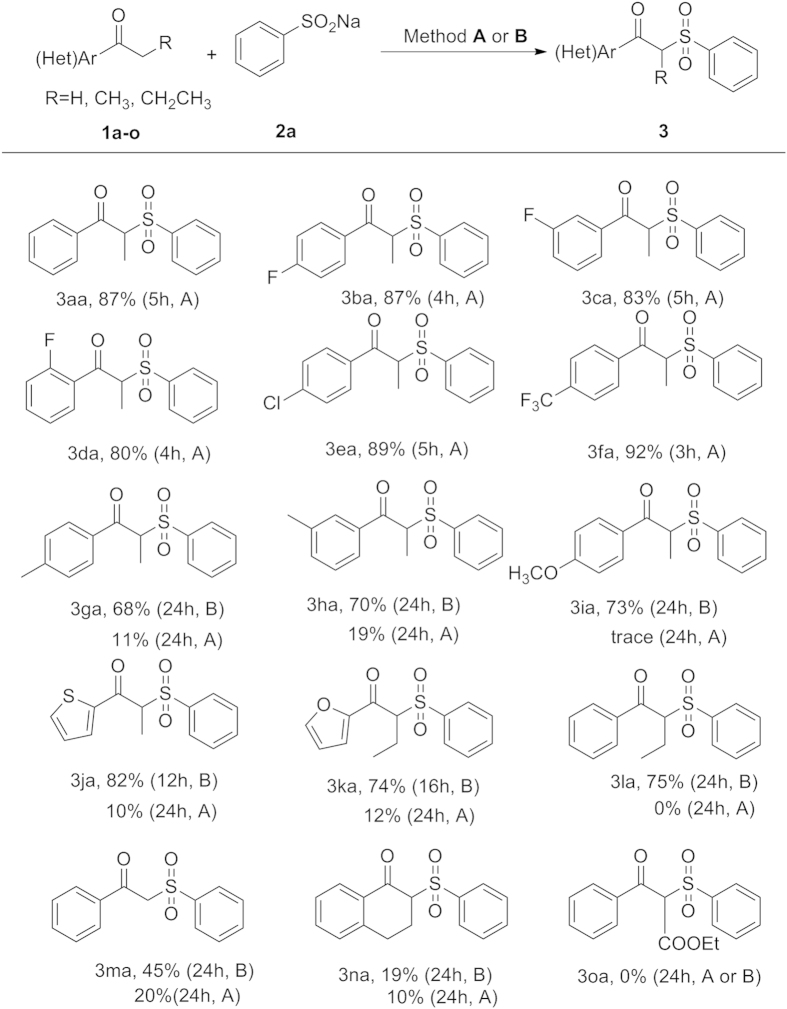
Scope of substrates 1. (**A**) Reaction conditions: (**1a–o**) (0.5 mmol), **2a** (1.0 mmol), and CuBr_2_ (20 mol%) and DBU (1 equiv) in 3 mL DMSO at room temperature in an open flask (in air). (**B**) Reaction conditions: (**1a–o**) (0.5 mmol), (**2a**) (1.0 mmol), and CuBr_2_ (20 mol%), 1,10-phenanthroline (20 mol%) and DBU (1 equiv) in 3 mL DMSO at room temperature in an open flask (in air).

With the optimal conditions in hand, we defined the scope of the reaction with respect to various aryl ketones and sodium sulfinates, and the results are summarized in Figs 2 and 3. Firstly, several sodium sulfinates were explored with **1a**. All the reactions, including sodium sulfinates with electron-withdrawing and electron-donating groups, proceeded smoothly to give their corresponding products (**3aa–3ai**) in moderate to good yields.

Subsequently, various aryl ketones were also investigated with **2a**. As listed in Fig. 3, we were pleased to find that electron-withdrawing substituents on aryl ring of ketone, such as halogen and trifluoromethyl functional groups, were well tolerated under the optimal reaction conditions, and gave the corresponding products (**3ba–fa**) in good yields. In this process, the steric factor was also verified by employing *para-, meta*- and *ortho***-**F**-**substituted propiophenones as substances. The similar yields indicated that this transformation was not affected by the steric factor. However, electron-donating substituents on aryl ring of ketone, such as methyl and methoxy, and electron-rich heteroaromatic ketone, such as thiophenes and furans, were subjected to this transformation under the optimal conditions (method **A**: reaction conditions: **1** (0.5 mmol), **2** (1.0 mmol), and CuBr_2_ (20 mol%) and DBU (1 equiv) in 3 mL DMSO at room temperature in an open flask (in air)), indicating that electronic factor plays the major role.

Given that copper salts own strong coordination ability with the nitrogen and phosphine-based ligands, we attempted to add a ligand into the mixtures. We focused on the less effective aryl ketone with electron-donating groups and compared these reactions with and without ligand in each case. Firstly, **1g** was chosen as the starting substrate to react with **2a** under standard conditions in the presence of a ligand (e.g. **L1–L6**). To our delight, it was found that the **1g** could be efficiently converted to the corresponding product **3ga** in the assistance of **L2** or **L3** as ligand (Table 2). The ligand effect is obviously demonstrated in these reactions, we speculated that the coordinated nitrogen-based ligands can also withdraw electrons from electron-rich arenes leading to enable yield. Moreover, the methyl substituted propiophenone at *meta* position (**1h**) was also explored to compare with **1g**, it was found that a similar yield (70%) was obtained. Finally, the **L3** was regarded as the most appropriate ligand for the copper salt. With the optimal ligand **L3** identified, treatment of aryl ketones bearing methoxy under the modified conditions (method **B**: reaction conditions: **1** (0.5 mmol), **2** (1.0 mmol), and CuBr_2_ (20 mol%) and DBU (1 equiv) in 3 mL DMSO at room temperature in an open flask (in air)), as expected, gave the desired product in good yield (**3ia**). In addition, under the modified conditions, heteroaromatic ketones (**3ja** and **3ka**) were also effective coupling partners, delivering corresponding products in high yields. *n*-Butyrophenone (**3la**) and acetophenone (**3ma**) are successively explored, 75% and 45% yield were achieved, respectively. Unfortunately, a lower yield was obtained when *α*-tetralone (**3na**) was used as the substrate. Notably, the use of ethyl benzoylacetate (**3oa**) did not provide product.

**Figure 4:**
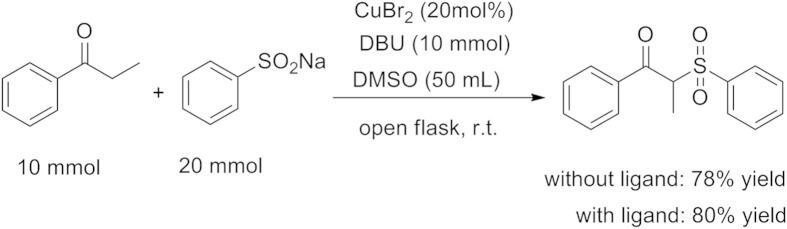
A scale-up experiment.

We conducted a scale-up experiment using **1a** (10 mmol) (Fig. 4). This reaction proceeded smoothly in the scale-up experiment, and resulted in a yield of up to 80%.

## Discussion

**Figure 5:**
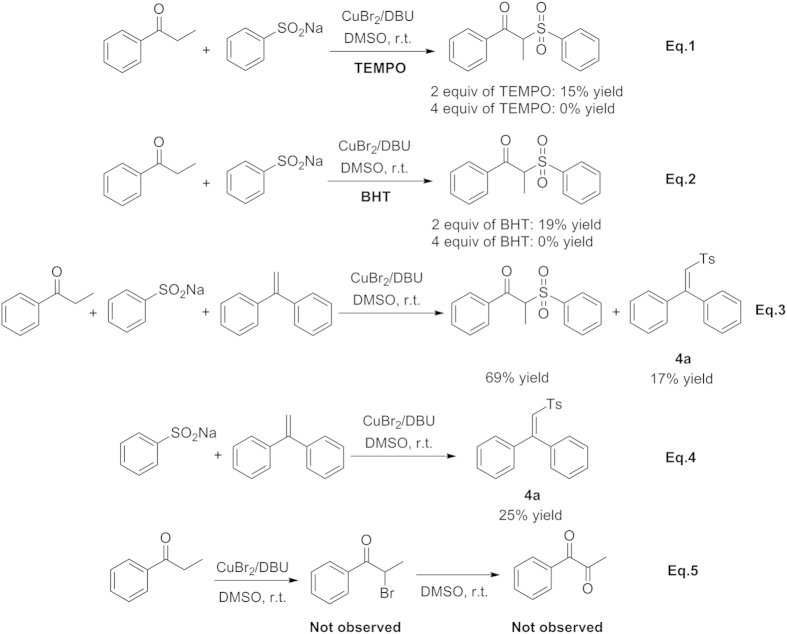
Mechanistic studies and control experiments.

To investigate the reaction mechanism, several control experiments were performed, as shown in Fig. 5. Firstly, the radical scavenger 2,2,6,6-tetramethyl-piperidine-1-oxyl (TEMPO) (Fig. 5, Eq. 1) and butylated hydroxytoluene (BHT) (Fig. 5, Eq. 2) were employed under the optimized conditions, respectively. In accordance with the references^27^,^28^,^29^,^30^, when 2.0 equiv of TEMPO or BHT (2 times to **1a** ) was added to the reaction, **3aa** was obtained in low yield. However, this transformation was completely inhibited with the addition of 4.0 equiv of TEMPO or BHT to the system, indicating a radical pathway should be involved in titled reaction. To further verify this point, we attempted to utilize 1,1-diphenylethylene to capture the radical. We tested the reaction with **1a** and 1,1-diphenylethylene in the present or absence of **2a** under the method **A** (Fig. 5, Eq. 3 and Eq. 4). A decreasing yield of **3aa** and a 17% yield of desired product **4a** were achieved in the present of **2a**; in the absence of **2a**, a 25% yield of **4a** was given, which can be interpreted as the evidence that sulfonyl radical was generated in this transformation. In view of the fact that carbonyl substrates could undergo bromination at the *α*-position *via* copper-boundenolate to generate *α*-bromo carbonyl, as MacMillan *et al.* have described^31^. Thus, we designed the experiment that propiophenone itself reacted in the absence of sodium benzenesulfinate under the optimal conditions, but no *α*-bromo propiophenone was detected (Fig. 5, Eq. 5). Considering that *α*-bromo propiophenon can be quickly oxidized to 1,2-dicarbonyl compound^32^ leading to the complete consumption of *α*-bromo propiophenone, we further prolonged the reaction time, but no products was observed. Afterwards, the reaction of propiophenone with superstoichiometric amounts of copper (II) bromide was carried out, however, no *α*-bromo propiophenone or other products were still formed. Thus, the possibility was firstly eliminated.

**Figure 6:**
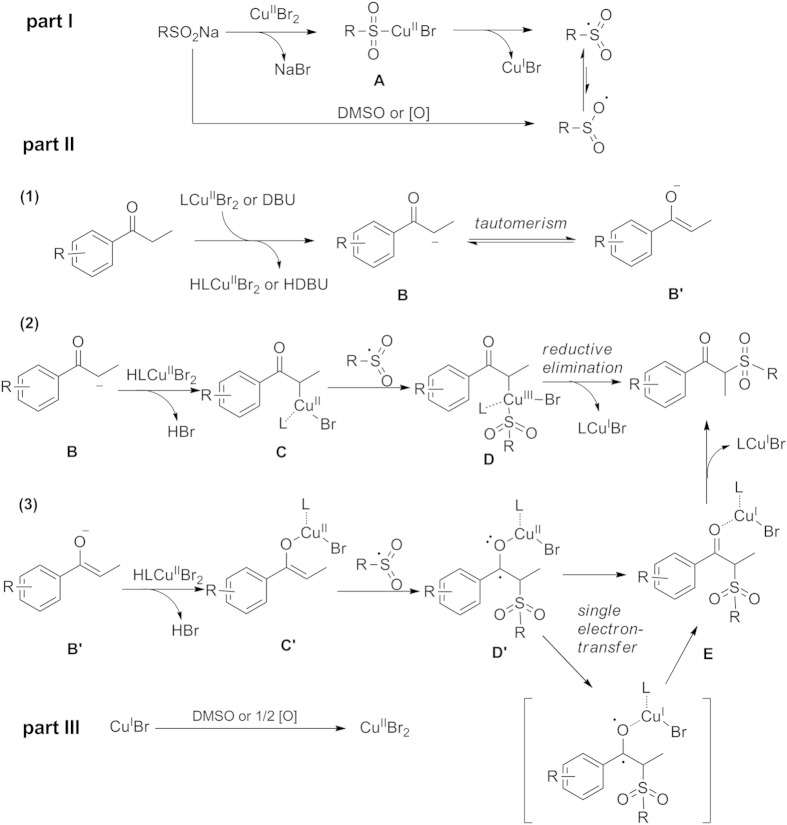
Possible mechanistic pathways.

Based on the preliminary results and previous reports, a plausible mechanism of this transformation is proposed (Fig. 6). A free radical pathway *via* C(sp^3^)-H bond functionalization of aryl ketones with sodium sulfinates is involved. We divided the mechanism into three parts. Part I, the intermediate **A** is generated by the coordination of copper to the sodium sulfinate^28^,^29^,^33^, and then the sulfonyl free radical intermediate is produced through the release of CuBr^28^,^29^,^34^. In addition, sodium sulfinate can also be quickly oxidized by DMSO *via* single electron-transfer (SET) to an oxygen centered radical resonating with the sulfonyl radical intermediate^9^,^29^. Part II, aryl ketones undergo deprotonation under LCu(II)Br_2_ and/or DBU involving *in situ* dehydrogenation of a ketone to intermediate **B** and **B’** which exists the tautomerism, and released the HLCu(II)Br_2_ and/or HDBU (part II, (1)). Afterwards, the intermediate **B** can be converted to alkylcopper intermediate **C**^35^,^36^, and then the intermediate **C** is subsequently attacked by sulfonyl radical to form the Cu(III) intermediate **D**, which undergoes a reductive elimination to give the target product with the release of LCu(I)Br (part II, (2))^27^,^37^. Simultaneously, the intermediate **B’** with HLCu(II)Br_2_ can be transformed to a copper enolate intermediate **C’**. The sulfonyl radical intermediate undergoes coupling with the copper enolate **C’** to generate a copper-coordinated ketyl radical **D’**, which forms copper-coordinated product **E** by a final SET and releases the LCu(I)Br. Finally, the copper-coordinated product **E** can be smoothly converted to the target product (part II, (3))^38^,^39^. Part III, the Cu(I) is oxidized to regenerate the Cu(II) species^27^ thereby forming a copper oxidative and reductive recycle process. However, the specific process of the ligand promoting this transformation is still not clear yet, but further studies on this are ongoing.

## Conclusion

In conclusion, we have developed a robust and efficient one-pot synthetic approach to the synthesis of complex *α*-alkyl-*β*-keto sulfones *via* C(sp^3^)-H bond functionalization followed by C(sp^3^)-S bond formation of aryl ketones with sodium sulfinates using copper (II) bromide and DBU or 1,10-phenanthroline under mild conditions. Preliminary mechanism revealed that a radical process may be involved in this method. In this reaction, substituted aryl ketones with electron-withdrawing group do not need any ligand to give a good to excellent yield; and substituted aryl ketones with electron-donating group and electron-rich heteroaromatic ketones offer good to excellent yield only under the nitrogen-based ligands. Further research on electronic effects of aryl ketones is also proceeding in our group.

## Methods

CuBr_2_ (20 mol%), DBU (1 equiv) and 1,10-phenanthroline (20 mol%) (It was employed in the mixtures under the modified conditions) were added into a mixture of aryl ketones (0.5 mmol, 1 equiv), sodium sulfinates (1.0 mmol, 2 equiv) in solvent (3 mL) at room temperature in open flask for the requisite period of time while monitoring the reaction progress by TLC. After completion of the reaction determined by TLC, the reaction mixture was poured into water (10 mL) and extracted with CH_2_Cl_2_ (3 × 25 mL), and the organic phase was dried over anhydrous MgSO_4_. The combined organic phase and washings were concentrated by a rotary evaporator, and the residue was purified by column chromatography on silica gel (200–300 mesh) using petroleum ether and ethyl acetate as eluent to provide the desired product.

## Additional Information

**How to cite this article**: Lan, X.-W. *et al.* Ligand-Mediated and Copper-Catalyzed C(sp^3^)-H Bond Functionalization of Aryl Ketones with Sodium Sulfinates under Mild Conditions. *Sci. Rep.* **5**, 18391; doi: 10.1038/srep18391 (2015).

## Supplementary Material

Supplementary Information

## Figures and Tables

**Figure 1:**
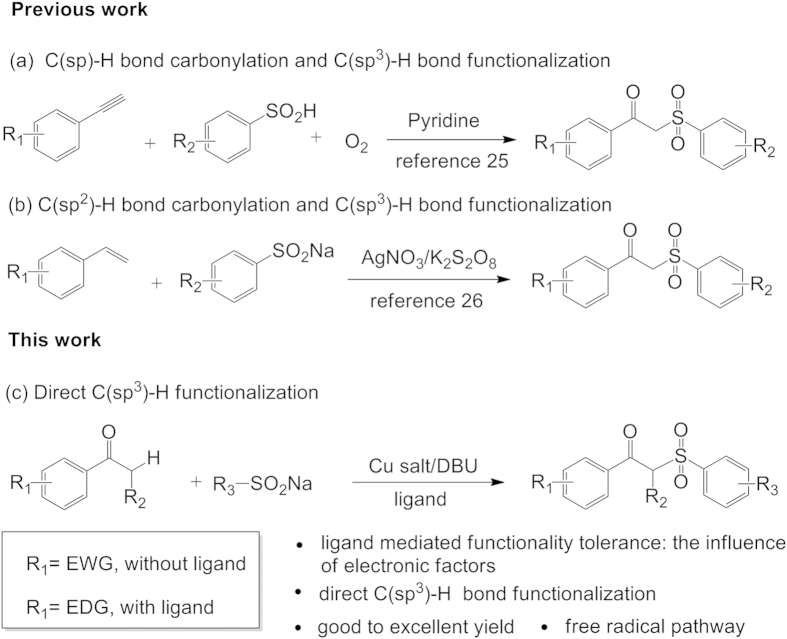
Synthetic methods for the *β*-keto sulfones.

**Table 1:**
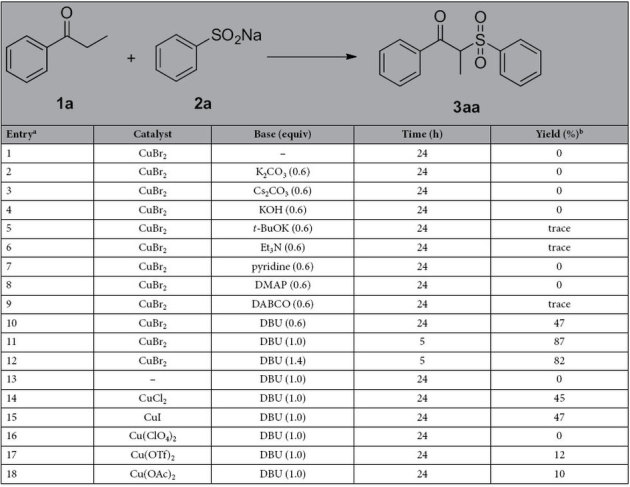
Optimization of reaction conditions.

^a^Reaction conditions: **1a** (0.5 mmol), **2a** (1.0 mmol), and catalyst (20 mol%) and base in 3 mL solvent at room temperature in an open flask (in air). ^b^Isolated yields.

**Table 2:**
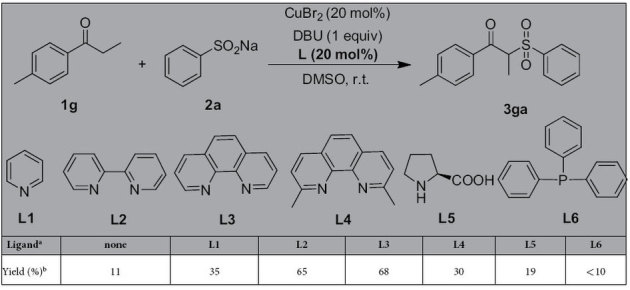
The investigation of ligand for copper salt in the reaction.

^a^Reaction conditions: 1**g** (0.5 mmol), 2**a** (1.0 mmol), CuBr_2_ (20 mol%), DBU (1 equiv) and ligand (20 mol%) in 3 mL DMSO at room temperature in an open flask (in air). ^b^Isolated yields.
