# Supplementary material for: Ligand-Mediated and Copper-Catalyzed C(sp3)-H Bond Functionalization of Aryl Ketones with Sodium Sulfinates under Mild Conditions
Source: Sci Rep. 2015 Dec 18;5:18391. doi: 10.1038/srep18391 (PMC4683520; doi:10.1038/srep18391)
Supplement: Supplementary Information [file srep18391-s1.pdf]

Supporting Information

Ligand-Mediated and Copper-Catalyzed C(sp^3)-H Bond Functionalization of Aryl Ketones with Sodium Sulfinates under Mild Conditions

Xing-Wang Lan,[1] Nai-Xing Wang,[1,*] Cui-Bing Bai,[1] Wei Zhang,[1] Yalan Xing,[2,*] Jia-Long Wen[3], Yan-Jing Wang[1] & Yi-He Li,[1]

[1]*Technical Institute of Physics and Chemistry, Chinese Academy of Sciences, Beijing, 100190, China*

E-Mail: nxwang@mail.ipc.ac.cn; Tel.: +86-10-82543575; Fax: +86-10-62554670.

[2]*Department of Chemistry, William Paterson University of New Jersey, 300 Pompton Road, Wayne, New Jersey 07470, United States.*

E-Mail: xingy@wpunj.edu

[3]*Beijing Key Laboratory of Lignocellulosic Chemistry, Beijing Forestry University, Beijing, 100083, P. R.China.*

Table of Contens

1. General Information 1

2. Experimental Section 1

3. Segmental Experiment Data 1

4. Characterization Data of All Products 1

5. Copies of NMR Spectra of All Products 4

6. Copies of HRMS Spectra of All Products 28

7. Copy of HSQC Spectrum of 3aa 39

8. Copy of HMBC Spectrum of 3aa 40

1. General Information.

Unless otherwise specified, all commercially available reagents were purchased from chemical suppliers without further purification. In particular, 1,8-diazabicyclo[5.4.1]undec-7-ene (DBU) was dried over 4 Å molecular sieves. ^{1}H NMR (400 MHz) and ^{13}C NMR (100 MHz) spectra were recorded in $CDCl_3$ with TMS as internal standard at room temperature. High-resolution mass spectra (HRMS) were obtained by ESI. Column chromatography was performed on silica gel (200-300 mesh). All products were characterized by comparison of ^{1}H NMR, ^{13}C NMR, and HRMS, especially the **3aa** was also characterized by using HSQC and HMBC to further confirm the structure.

2. Experimental Section

General Procedure for Preparation of Sodium Sulfinates (2b-2e, 2g-2i). [1]

4-Chlorobenzenesulfinate (**2d**) sodium salt was prepared by heating 2.5 g of sodium sulfite, 2.10 g of 4-chlorobenzenesulphonyl chloride, and 1.68 g of sodium bicarbonate in 10.0 mL of water at 70-80 ℃ for 4 h. After cooling to room temperature, water was removed by filtering under vacuum and the residue was extracted by ethanol, recrystallization or evaporation as a white solid. Similarly, other sodium arenesulfinates (**2b, 2c, 2e, 2g-2i**) was prepared from their corresponding sulphonyl chlorides.

3. Segmental Experiment Data

Table 1 Optimization of reaction conditions [a]

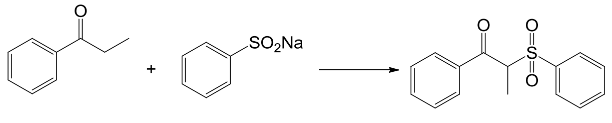

1a 2a 3aa

Entry	Catalyst	Base (equiv.)	Solvent	Time(h)	Yield(%)[b]
1	$CuBr_2$	DBU (1.0)	CH_3CN	24	23
2	$CuBr_2$	DBU (1.0)	CH_2Cl_2	24	<10
3	$CuBr_2$	DBU (1.0)	EtOH	24	<10
4	$CuBr_2$	DBU (1.0)	DMF	24	53
5	$CuBr_2$	DBU (1.0)	H_2O	24	0

[a] Reaction conditions: **1a** (0.5 mmol), **2a** (1.0 mmol), and catalyst (20 mol%) and base in 3 mL solvent at room temperature in open flask (air). [b] Isolated yields.

4. Characterization Data of All Products.

*1-phenyl-2-(phenylsulfonyl)propan-1-one (**3aa**):* White solid, mp 85-86℃. ^{1}H NMR (400 MHz, $CDCl_3$) δ 7.96 – 7.94(m, 2H), 7.80 – 7.77(m, 2H), 7.64 – 7.57(m, 2H), 7.52 – 7.43(m, 2H), 5.17 (dd, J_1 = 14.0 Hz, J_2=6.8 Hz, 1H), 1.57 (d, J = 7.2 Hz, 3H). ^{13}C NMR (100 MHz, $CDCl_3$) δ 192.45, 136.25, 136.23, 134.18, 134.03, 129.73, 129.10, 128.88, 128.74, 64.97, 13.11. HRMS (ESI, *m/z*) calcd. for $C_{15}H_{14}O_3SNa$ $[M+Na]^+$ 297.0556, found 297.0559.

*1-phenyl-2-tosylpropan-1-one (**3ab**):* White solid, mp 101-102℃. ^{1}H NMR (400 MHz, $CDCl_3$) δ 7.98 – 7.96 (m, 2H), 7.65 (d, J = 8.4 Hz, 2H), 7.60 (t, J = 7.4 Hz, 1H), 7.47 (t, J = 7.8 Hz, 2H), 7.30 (d, J = 8.1 Hz, 2H), 5.15 (dd, J_1= 13.8 Hz, J_2 = 7.0 Hz, 1H), 2.42 (s, 3H), 1.55 (d, J = 6.9 Hz, 3H). ^{13}C NMR (100 MHz, $CDCl_3$) δ 192.60, 145.30, 136.31, 133.94, 133.16, 129.80, 129.50, 129.15, 128.70, 65.03, 21.63, 13.17. HRMS (ESI, *m/z*) calcd. for $C_{16}H_{16}O_3SNa$ $[M+Na]^+$ 311.0712,

found 311.0713.

*2-((4-fluorophenyl)sulfonyl)-1-phenylpropan-1-one (**3ac**):* White solid, mp 113-114℃. ^{1}H NMR (400 MHz, $CDCl_3$) δ 7.90 – 7.87 (m, 2H), 7.75 – 7.70 (m, 2H), 7.56 – 7.52 (m, 1H), 7.40 (t, J = 7.8 Hz, 2H), 7.13 – 7.09 (m, 2H), 5.10 (dd, J_1 = 7.0 Hz, J_2 = 3.4 Hz, 1H), 1.49 (d, J = 6.9 Hz, 3H). ^{13}C NMR (100 MHz, $CDCl_3$) δ 192.57, 167.49, 164.93, 136.09, 134.22, 132.84, 132.74, 132.05, 132.02, 129.13, 128.84, 116.35, 116.12, 65.04, 13.28. HRMS (ESI, *m/z*) calcd. for $C_{15}H_{13}FO_3SNa$ $[M+Na]^+$ 315.0462, found 315.0461.

*2-((4-chlorophenyl)sulfonyl)-1-phenylpropan-1-one (**3ad**):* White solid, mp 113-118℃. ^{1}H NMR (400 MHz, $CDCl_3$) δ 7.91 – 7.89 (m, 2H), 7.66 – 7.64 (m, 2H), 7.58 – 7.54 (m, 1H), 7.44 – 7.41 (m, 4H), 5.10 (dd, J_1 = 7.0 Hz, J_2 = 3.6 Hz, 1H), 1.50 (d, *J* = 6.9 Hz, 3H). ^{13}C NMR (100 MHz, $CDCl_3$) δ 192.47, 141.19, 136.06, 134.42, 134.25, 131.35, 129.22, 129.15, 128.86, 65.10, 13.31. HRMS (ESI, *m/z*) calcd. for $C_{15}H_{13}ClO_3SNa$ $[M+Na]^+$ 331.0166, found 331.0166.

*2-((4-bromophenyl)sulfonyl)-1-phenylpropan-1-one (**3ae**):* White solid, mp 149-151 ℃. ^{1}H NMR (400 MHz, $CDCl_3$) δ 7.90 – 7.88 (m, 2H), 7.61 – 7.54 (m, 5H), 7.42 (t, *J* = 8.0 Hz, 2H), 5.09 (dd, J_1 = 13.8 Hz, J_2 = 7.0 Hz, 1H), 1.50 (d, *J* = 7.0 Hz, 3H). ^{13}C NMR (100 MHz, $CDCl_3$) δ 192.45, 136.05, 134.97, 134.25, 132.22, 131.39, 130.90, 129.86, 129.15, 128.86, 77.34, 77.02, 76.70, 65.55, 65.09, 30.59, 19.19, 13.71, 13.30. HRMS (ESI, *m/z*) calcd. for $C_{15}H_{13}BrO_3SNa$ $[M+Na]^+$ 376.9641, found 376.9642.

*2-(methylsulfonyl)-1-phenylpropan-1-one (**3af**):* Yellow oil liquid. ^{1}H NMR (400 MHz, $CDCl_3$) δ 8.01 – 7.99 (m, 2H), 7.61 (t, *J* = 7.6 Hz, 1H), 7.49 (t, *J* = 7.6 Hz, 2H), 4.96 (dd, J_1 = 14.2 Hz, J_2 = 7.0 Hz, 1H), 2.94 (s, 3H). ^{13}C NMR (100 MHz, $CDCl_3$) δ 193.97, 135.73, 134.43, 129.16, 128.93, 63.95, 37.04, 13.76. HRMS (ESI, *m/z*) calcd. for $C_{10}H_{12}O_3SNa$ $[M+Na]^+$ 235.0399, found 235.0397.

*2-(cyclopropylsulfonyl)-1-phenylpropan-1-one (**3ag**):* Yellow oil liquid. ^{1}H NMR (400 MHz, $CDCl_3$) δ 7.97 (d, *J* = 7.7 Hz, 2H), 7.57 (t, *J* = 7.2 Hz, 1H), 7.45 (t, *J* = 7.6 Hz, 2H), 4.99 (dd, J_1 = 14.0 Hz, J_2 = 7.2 Hz, 1H), 2.52 – 2.46 (m, 1H), 1.69 (d, *J* = 7.1 Hz, 3H), 1.20 – 1.17 (m, 1H), 1.15 – 1.05 (m, 1H), 1.04 – 0.97 (m, 1H), 0.93 – 0.86 (m, 1H). ^{13}C NMR (100 MHz, $CDCl_3$) δ 194.05, 136.91, 134.87, 129.85, 129.56, 64.88, 28.16, 13.87, 6.30, 4.74. HRMS (ESI, *m/z*) calcd. for $C_{12}H_{15}O_3S$ $[M+H]^+$ 239.0736, found 239.0735.

*1-phenyl-2-((4-(trifluoromethyl)phenyl)sulfonyl)propan-1-one (**3ah**):* White solid, mp 94-97℃. ^{1}H NMR (400 MHz, $CDCl_3$) δ 7.90 – 7.85 (m, 4H), 7.72 (d, *J* = 8.4 Hz, 2H), 7.56 (t, *J* = 7.2 Hz, 1H), 7.42 (t, *J* = 7.6 Hz, 2H), 5.13 (dd, J_1= 13.6 Hz, J_2 = 6.8 Hz, 1H), 1.52 (d, *J* = 7.0 Hz, 3H). ^{13}C NMR (100 MHz, $CDCl_3$) δ 192.30, 139.49, 135.90, 134.39, 130.60, 129.12, 128.91, 126.02, 125.98, 125.94, 125.91, 65.11, 13.29. HRMS (ESI, *m/z*) calcd. for $C_{16}H_{14}F_3O_3S$ $[M+H]^+$ 343.0610, found 343.0608.

*2-(naphthalen-2-ylsulfonyl)-1-phenylpropan-1-one (**3ai**):* White solid, mp 119-121℃. ^{1}H NMR (400 MHz, $CDCl_3$) δ 8.29 (s, 1H), 7.91 – 7.83 (m, 5H), 7.68 (dd, J_1 = 8.6 Hz, J_2 = 0.8 Hz, 1H), 7.63 – 7.47 (m, 3H), 7.36 (t, *J* = 4.0 Hz, 2H), 5.17 (dd, J_1 = 6.8 Hz, J_2 = 3.4 Hz, 1H), 1.54 (d, *J* = 6.9 Hz, 3H). ^{13}C NMR (100 MHz, $CDCl_3$) δ 192.64, 136.59, 135.74, 134.05, 133.70, 132.17, 132.07, 129.73, 129.61, 129.25, 129.15, 128.84, 128.09, 127.73, 124.34, 65.56, 13.34. HRMS (ESI, *m/z*) calcd. for $C_{19}H_{16}O_3SNa$ $[M+Na]^+$ 347.0712, found 347.0712.

*1-(4-fluorophenyl)-2-(phenylsulfonyl)propan-1-one (**3ba**):* White solid, mp 116-117 ℃. ^{1}H NMR (400 MHz, $CDCl_3$) δ 8.04 – 8.01 (m, 2H), 7.79 – 7.76 (m, 2H), 7.68 – 7.64 (m, 1H), 7.55 – 7.51 (m, 2H), 7.17 – 7.13 (m, 2H), 5.11 (dd, J_1 = 13.8 Hz, J_2 =7.0 Hz, 1H), 1.56 (d, *J* =

6.9 Hz, 3H). ^{13}C NMR (100 MHz, $CDCl_3$) δ 190.79, 167.60, 134.27, 132.04, 131.95, 129.77, 128.90, 116.07, 115.85, 65.10, 13.11. HRMS (ESI, *m/z*) calcd. for $C_{15}H_{13}FO_3SNa$ $[M+Na]^+$ 315.0462, found 315.0464.

*1-(3-fluorophenyl)-2-(phenylsulfonyl)propan-1-one (**3ca**):* White solid, mp 110-112℃. ^{1}H NMR (400 MHz, $CDCl_3$) δ 7.72 – 7.70 (m, 3H), 7.62 – 7.55 (m, 2H), 7.48 – 7.37 (m, 3H), 7.26 – 7.22 (m, 1H), 5.03 (dd, J_1 = 13.6 Hz, J_2 = 7.6 Hz, 1H), 1.50 (d, J = 6.9 Hz, 3H). ^{13}C NMR (100 MHz, $CDCl_3$) δ 191.41, 191.39, 164.03, 161.56, 138.31, 138.25, 135.92, 134.37, 130.50, 130.42, 129.80, 128.98, 125.05, 125.02, 121.27, 121.05, 115.90, 115.67, 65.29, 13.12. HRMS (ESI, *m/z*) calcd. for $C_{15}H_{13}FO_3SNa$ $[M+Na]^+$ 315.0462, found 315.0461.

*1-(2-fluorophenyl)-2-(phenylsulfonyl)propan-1-one (**3da**):* Yellow oil liquid. ^{1}H NMR (400 MHz, $CDCl_3$) δ 7.74 – 7.66 (m, 3H), 7.58 – 7.53 (m, 1H), 7.49 – 7.42 (m, 3H), 7.19 – 7.13 (m, 1H), 7.02 (ddd, J_1 = 11.6 Hz, J_2=8.3 Hz, J_3 = 0.8 Hz, 1H), 5.15 (m, 1H), 1.52 (dd, J_1 = 6.8 Hz, J_2 = 0.4 Hz, 3H). ^{13}C NMR (100 MHz, $CDCl_3$) δ 190.98, 190.94, 162.78, 160.26, 136.82, 135.61, 135.52, 134.13, 131.13, 131.11, 129.55, 128.92, 125.64, 125.53, 124.81, 124.78, 116.88, 116.64, 69.20, 69.11, 12.39. HRMS (ESI, *m/z*) calcd. for $C_{15}H_{14}FO_3S$ $[M+H]^+$ 293.0642, found 293.0641.

*1-(4-chlorophenyl)-2-(phenylsulfonyl)propan-1-one (**3ea**):* White solid, mp 148-150 ℃. ^{1}H NMR (400 MHz, $CDCl_3$) δ 7.95 – 7.91 (m, 2H), 7.79 – 7.76 (m, 2H), 7.69 – 7.65 (m, 1H), 7.55 – 7.51 (m, 2H), 7.48 – 7.44 (m, 2H), 5.10 (dd, J_1 = 13.6 Hz, J_2 = 6.8 Hz, 1H), 1.56 (d, J = 6.9 Hz, 3H). ^{13}C NMR (100 MHz, $CDCl_3$) δ 191.25, 140.80, 135.90, 134.57, 134.30, 130.56, 129.76, 129.09, 128.92, 65.14, 13.08. HRMS (ESI, *m/z*) calcd. for $C_{15}H_{13}ClO_3SNa$ $[M+Na]^+$ 331.0166, found 331.0162.

*2-(phenylsulfonyl)-1-(4-(trifluoromethyl)phenyl)propan-1-one (**3fa**):* White solid, mp 140-142 ℃. ^{1}H NMR (400 MHz, $CDCl_3$) δ 8.10 (d, J = 8.2 Hz, 2H), 7.79 – 7.74 (m, 4H), 7.70 – 7.66 (m, 1H), 7.56 – 7.52 (m, 2H), 5.15 (dd, J_1 = 14.0 Hz, J_2 = 6.8 Hz, 1H), 1.58 (d, J = 6.9 Hz, 3H). ^{13}C NMR (100 MHz, $CDCl_3$) δ 191.73, 138.90, 135.79, 135.34, 134.41, 129.74, 129.51, 129.00, 125.80, 125.76, 65.47, 13.02. HRMS (ESI, *m/z*) calcd. for $C_{16}H_{13}F_3O_3SNa$ $[M+Na]^+$ 365.0430, found 365.0432.

*2-(phenylsulfonyl)-1-(p-tolyl)propan-1-one (**3ga**):* White solid, mp 103-104 ℃. ^{1}H NMR (400 MHz, $CDCl_3$) δ 7.87 (d, J = 8.2 Hz, 2H), 7.80 – 7.78 (m, 2H), 7.65 (t, J = 7.0 Hz, 1H), 7.52 (t, J = 7.8 Hz, 2H), 7.27 (d, J = 9.4 Hz, 2H), 5.14 (dd, J_1 = 12.8 Hz, J_2 = 6.8 Hz, 1H), 2.42 (s, 3H), 1.56 (d, J = 6.9 Hz, 3H). ^{13}C NMR (100 MHz, $CDCl_3$) δ 191.89, 145.22, 136.18, 134.09, 133.78, 129.81, 129.45, 129.29, 128.81, 64.92, 21.69, 13.17. HRMS (ESI, *m/z*) calcd. for $C_{16}H_{16}O_3SNa$ $[M+Na]^+$ 311.0712, found 311.0710.

*2-(phenylsulfonyl)-1-(m-tolyl)propan-1-one (**3ha**):* White solid, mp 83-84 ℃. ^{1}H NMR (400 MHz, $CDCl_3$) δ 7.73 – 7.86 (m, 4H), 7.57 (t, J = 7.4 Hz, 1H), 7.44 (t, J = 8.0 Hz, 2H), 7.34 – 7.26 (m, 2H), 5.09 (dd, J_1 = 14.0 Hz, J_2 = 6.8 Hz, 1H), 2.33 (s, 3H), 1.49 (d, J = 6.8 Hz, 3H). ^{13}C NMR (100 MHz, $CDCl_3$) δ 192.68, 138.66, 136.26, 134.92, 134.19, 129.84, 129.57, 128.86, 128.67, 126.41, 64.97, 21.34, 13.20. HRMS (ESI, *m/z*) calcd. for $C_{16}H_{17}O_3S$ $[M+H]^+$ 289.0893, found 289.0897.

*1-(4-methoxyphenyl)-2-(phenylsulfonyl)propan-1-one (**3ia**):* White solid, mp 75-75 ℃. ^{1}H NMR (400 MHz, $CDCl_3$) δ 7.98 – 7.96 (m, 2H), 7.80 – 7.77 (m, 2H), 7.64 – 7.62 (m, 1H), 7.54 – 7.50 (m, 2H), 6.96 – 6.93 (m, 2H), 5.11 (dd, J_1 = 14.0 Hz, J_2 = 7.2 Hz, 1H), 3.88 (s, 3H), 1.55 (d, J = 6.9 Hz, 3H). ^{13}C NMR (100 MHz, $CDCl_3$) δ 190.50, 164.38, 136.18, 134.07,

131.65, 129.80, 129.27, 128.79, 113.99, 64.79, 55.57, 13.17. HRMS (ESI, *m/z*) calcd. for $C_{16}H_{16}O_4SNa$ $[M+Na]^+$ 327.0662, found 327.0658.

*2-(phenylsulfonyl)-1-(thiophen-2-yl)propan-1-one (**3ja**):* White solid, mp 109-111 ℃. ^1H NMR (400 MHz, $CDCl_3$) δ 7.83 – 7.81 (m, 3H), 7.74 – 7.73 (m, 1H), 7.67 – 7.63 (m, 1H), 7.52 (t, J = 7.8 Hz, 2H), 7.17 – 7.14 (m, 1H), 4.90 (dd, J_1 = 10.4 Hz, J_2 = 6.8 Hz, 1H), 1.59 (d, J = 7.0 Hz, 3H). ^{13}C NMR (100 MHz, $CDCl_3$) δ 184.50, 143.55, 135.99, 134.41, 134.23, 129.80, 128.89, 128.56, 67.04, 12.86. HRMS (ESI, *m/z*) calcd. for $C_{13}H_{12}O_3NaS_2$ $[M+Na]^+$ 303.0132, found 303.0127.

*1-(furan-2-yl)-2-(phenylsulfonyl)butan-1-one (**3ka**):* White solid, mp 99-100 ℃. ^1H NMR (400 MHz, $CDCl_3$) δ 7.81 – 7.78 (m, 2H), 7.64 – 7.59 (m, 2H), 7.52 – 7.48 (m, 2H), 7.29 (dd, J_1 = 3.6 Hz, J_2 = 0.8 Hz, 1H), 6.56 (dd, J_1 = 3.6 Hz, J_2 = 1.6 Hz, 1H), 4.79 (dd, J_1 = 11.2 Hz, J_2 = 4.0 Hz, 1H), 2.19 – 1.98 (m, 2H), 0.91 (t, J = 7.4 Hz, 3H). ^{13}C NMR (100 MHz, $CDCl_3$) δ 180.16, 152.93, 147.85, 136.77, 134.08, 129.61, 128.83, 119.62, 113.06, 72.17, 20.94, 11.43. HRMS (ESI, *m/z*) calcd. for $C_{14}H_{14}O_4SNa$ $[M+Na]^+$ 301.0505, found 301.0501.

*1-phenyl-2-(phenylsulfonyl)butan-1-one (**3la**):* White solid, mp 97-99 ℃. ^1H NMR (400 MHz, $CDCl_3$) δ 7.97 – 7.95 (m, 2H), 7.79 – 7.77 (m, 2H), 7.66 – 7.58 (m, 2H), 7.54 – 7.46 (m, 4H), 5.01 (dd, J_1 = 10.8 Hz, J_2 = 3.6 Hz, 1H), 2.23 – 2.01 (m, 2H), 0.89 (t, J = 7.2 Hz, 3H). ^{13}C NMR (100 MHz, $CDCl_3$) δ 192.59, 137.39, 136.57, 134.11, 133.97, 129.77, 128.92, 128.80, 128.76, 71.46, 22.01, 11.49. HRMS (ESI, *m/z*) calcd. for $C_{16}H_{16}O_3SNa$ $[M+Na]^+$ 311.0712, found 311.0712.

*1-phenyl-2-(phenylsulfonyl)ethanone (**3ma**):* White solid, mp 94-97 ℃. ^1H NMR (400 MHz, $CDCl_3$) δ 7.96 – 7.89 (m, 4H), 7.69 – 7.60 (m, 2H), 7.57 – 7.53 (m, 2H), 7.50 – 7.46 (m, 2H), 4.74 (s, 2H). ^{13}C NMR (100 MHz, $CDCl_3$) δ 187.91, 138.80, 135.77, 134.32, 134.19, 129.27, 129.17, 128.84, 128.58, 63.49. HRMS (ESI, *m/z*) calcd. for $C_{14}H_{12}O_3SNa$ $[M+Na]^+$ 283.0399, found 283.0396.

*2-(phenylsulfonyl)-3,4-dihydronaphthalen-1(2H)-one (**3na**):* Light yellow solid, mp 94-97 ℃. ^1H NMR (400 MHz, $CDCl_3$) δ 7.98 (d, J = 7.9 Hz, 1H), 7.92 (d, J = 7.8 Hz, 2H), 7.67 (t, J = 7.0 Hz, 1H), 7.59 – 7.50 (m, 3H), 7.34 – 7.28 (m, 2H), 4.12 (t, J = 5.6 Hz, 1H), 3.55 – 3.47 (m, 1H), 3.03 – 2.96 (m, 1H), 2.90 – 2.83 (m, 1H), 2.70 – 2.62 (m, 1H). ^{13}C NMR (100 MHz, $CDCl_3$) δ 188.63, 143.56, 139.00, 134.53, 134.02, 131.76, 129.13, 129.02, 128.97, 127.98, 127.09, 69.67, 26.61, 23.65. HRMS (ESI, *m/z*) calcd. for $C_{16}H_{14}O_3SNa$ $[M+Na]^+$ 309.0556, found 309.0554.

*(2-(phenylsulfonyl)ethene-1,1-diyl)dibenzene (**4a**):* ^1H NMR (400 MHz, $CDCl_3$) δ 7.50 (d, J = 7.6 Hz, 2H), 7.40 (t, J = 7.2 Hz, 1H), 7.30-7.18 (m, 8H), 7.13 (d, J = 7.6 Hz, 2H), 7.00 (d, J = 7.6 Hz, 2H), 6.95 (s, 1H). ^{13}C NMR (100 MHz, $CDCl_3$) δ 155.25, 141.50, 139.12, 135.49, 132.87, 130.37, 129.79, 128.91, 128.78, 128.71, 128.63, 128.24, 127.89, 127.66. HRMS (ESI, *m/z*) calcd. for $C_{20}H_{17}O_2S$ $[M+H]^+$ 321.0944, found 321.0943.

Reference

1 Xu, Y. L.; Tang, X. D.; Hu, W. G.; Wu, W. Q.; Jiang, H. F. *Green Chem.* **2014**, 16, 3720 – 3723.

5. Copies of NMR Spectra of All Products

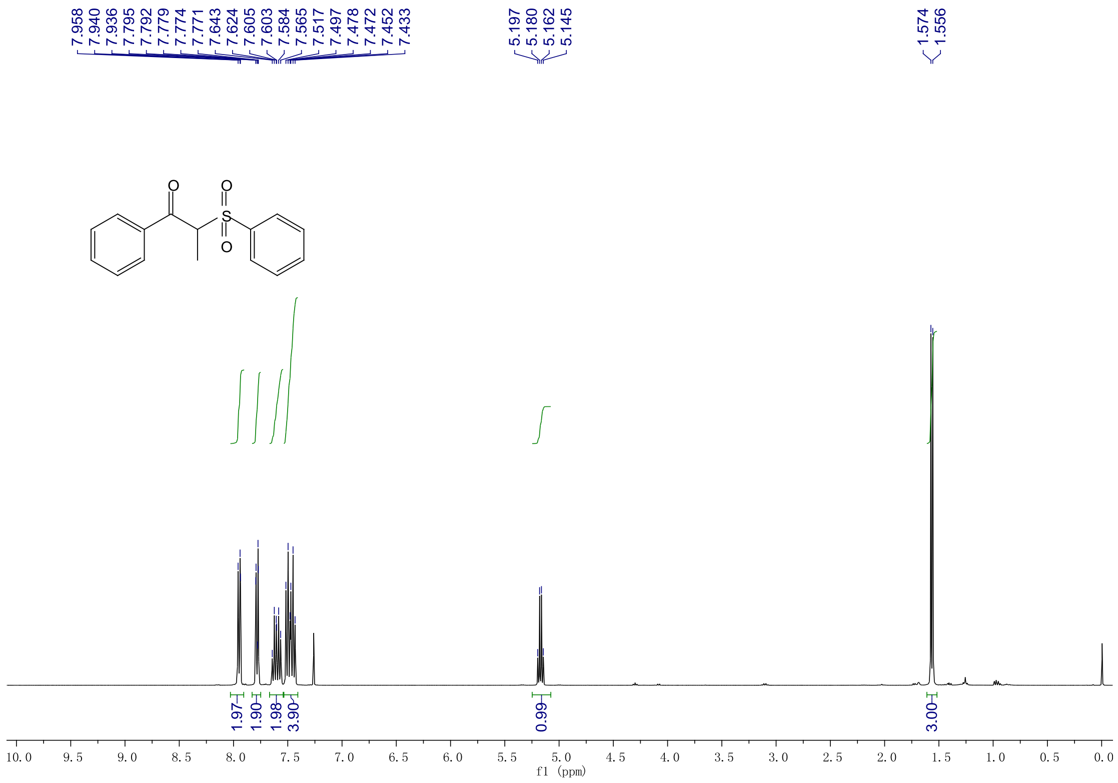

^{1}H NMR spectrum of product **3aa**

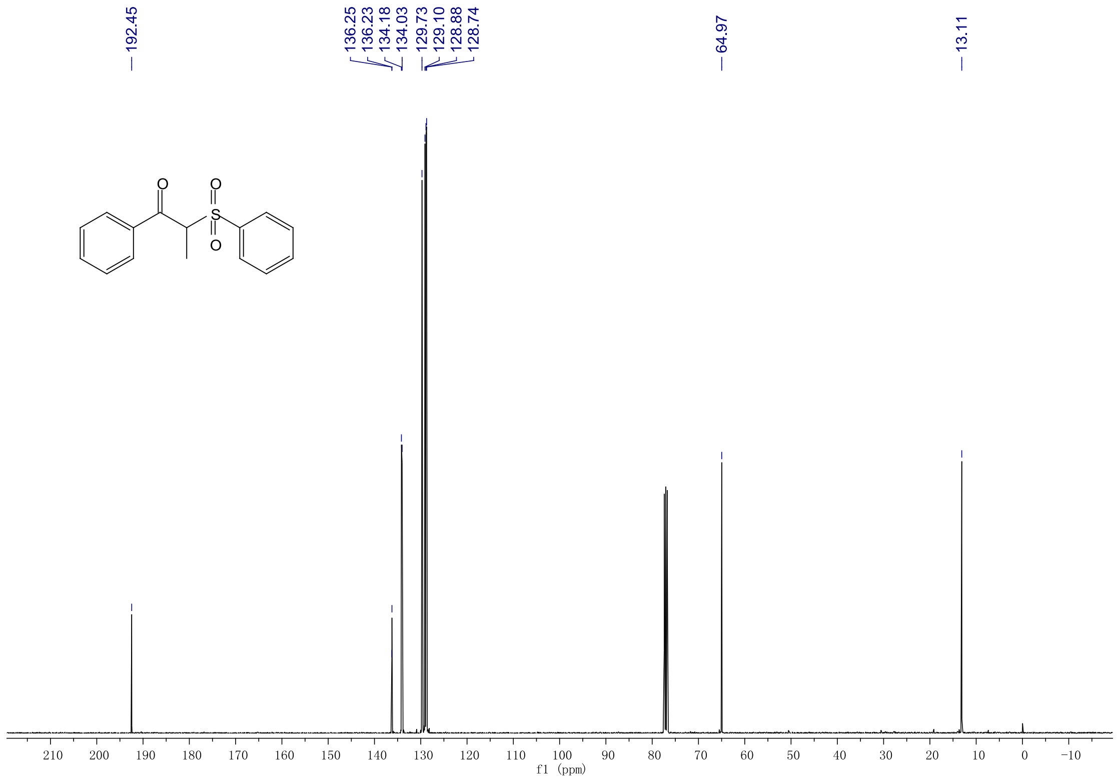

^{13}C NMR spectrum of product **3aa**

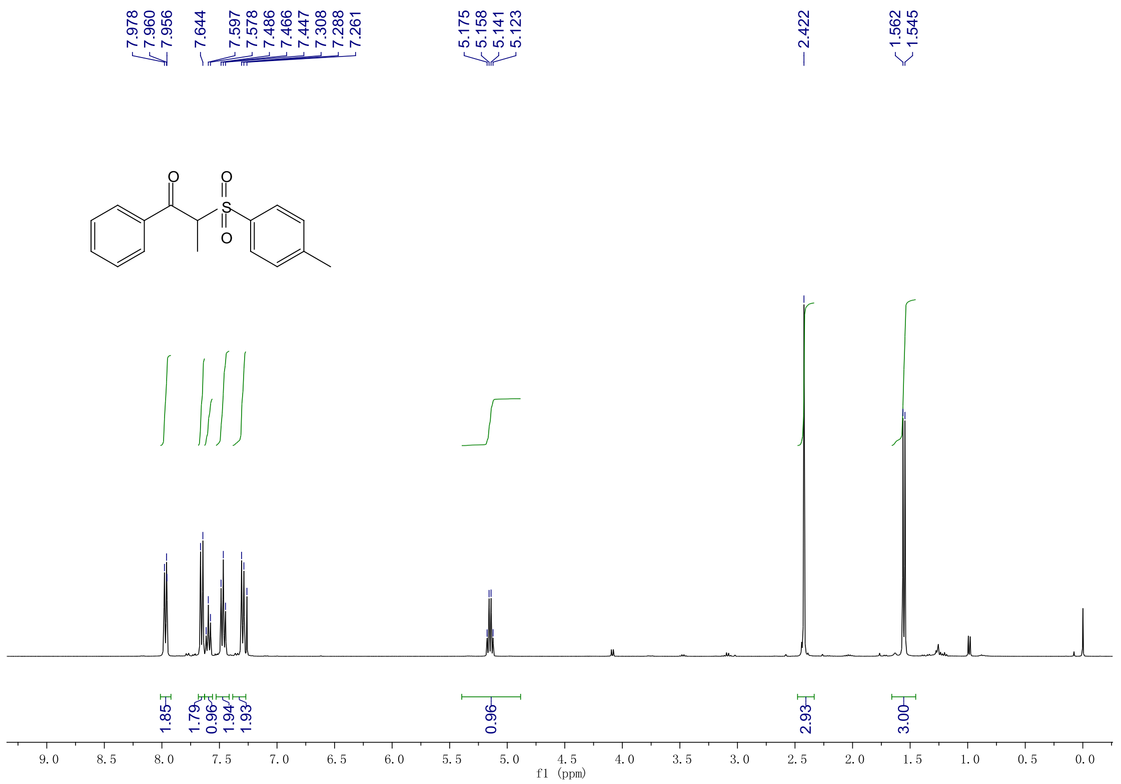

^{1}H NMR spectrum of product **3ab**

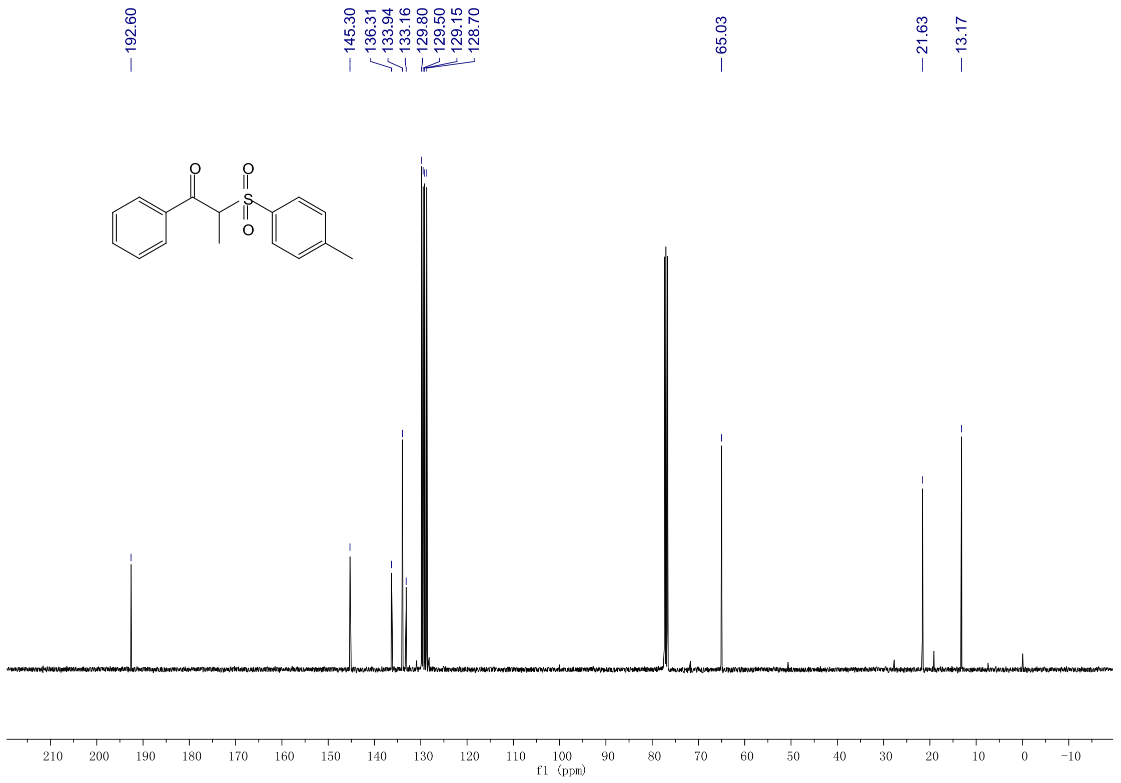

^{13}C NMR spectrum of product **3ab**

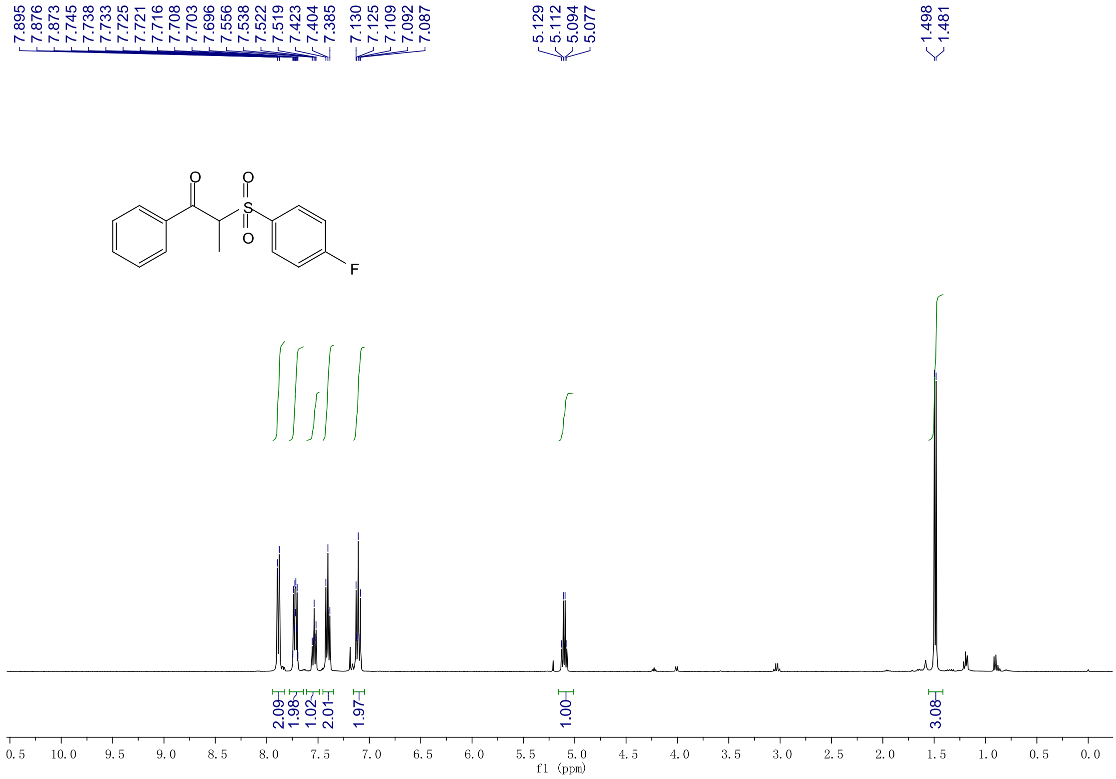

^{1}H NMR spectrum of product **3ac**

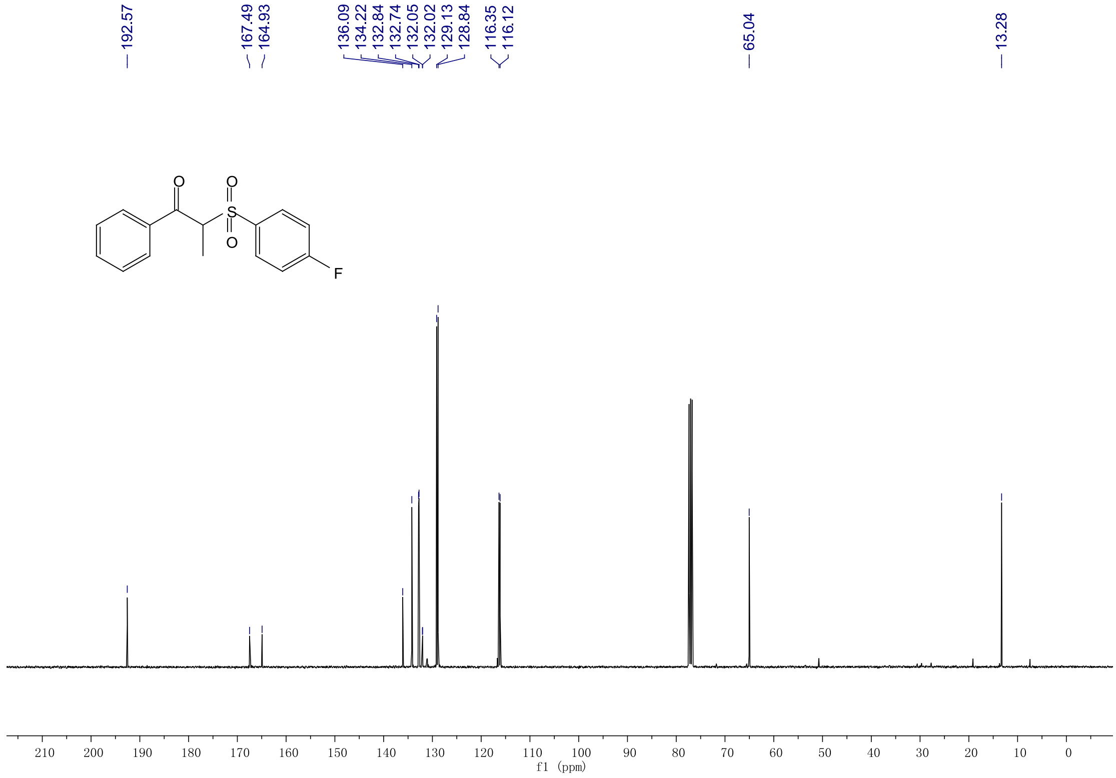

^{13}C NMR spectrum of product **3ac**

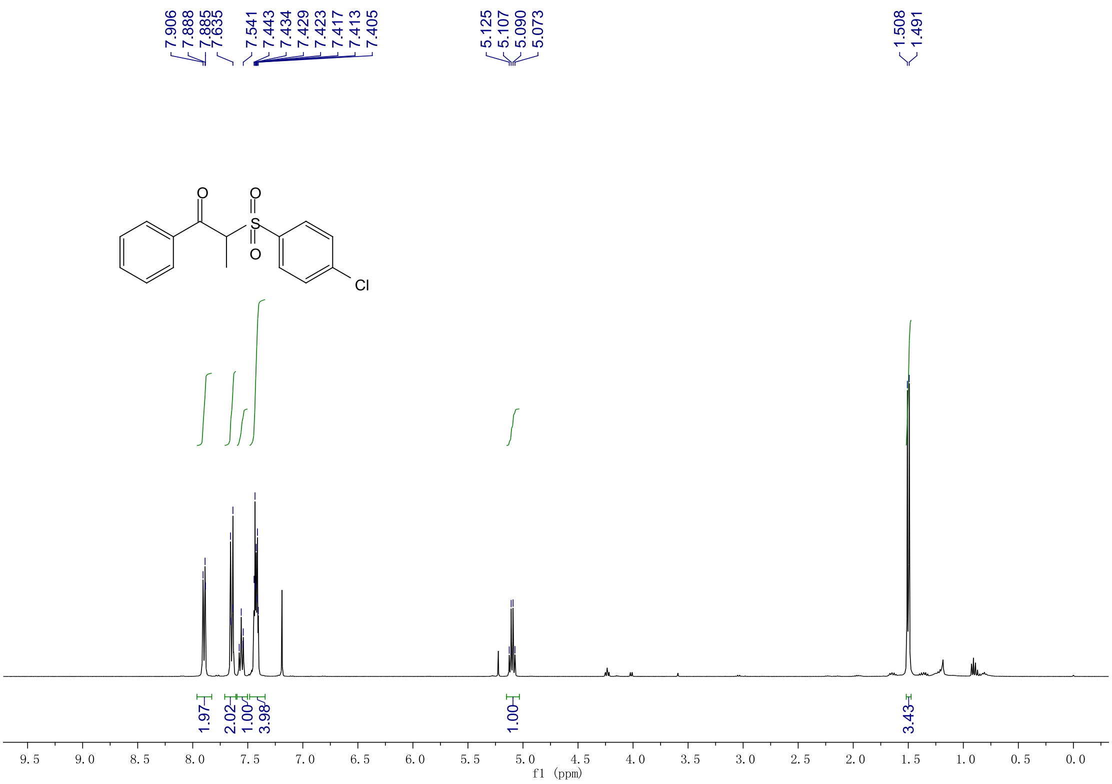

^{1}H NMR spectrum of product **3ad**

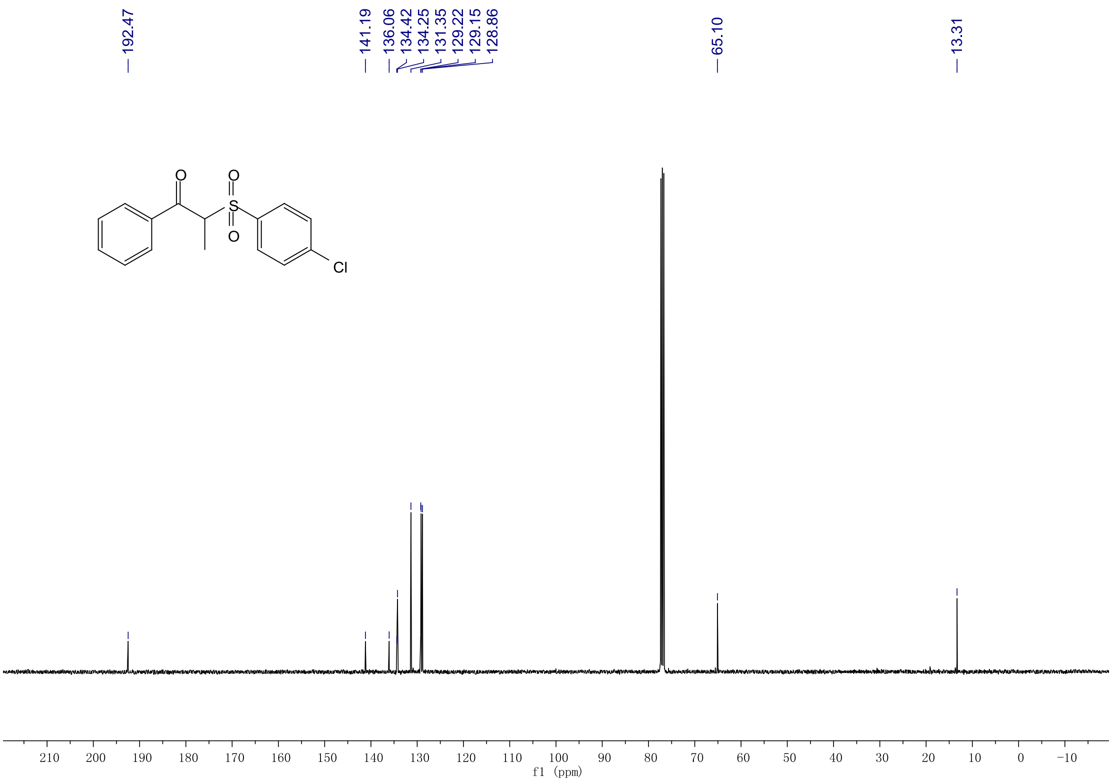

^{13}C NMR spectrum of product **3ad**

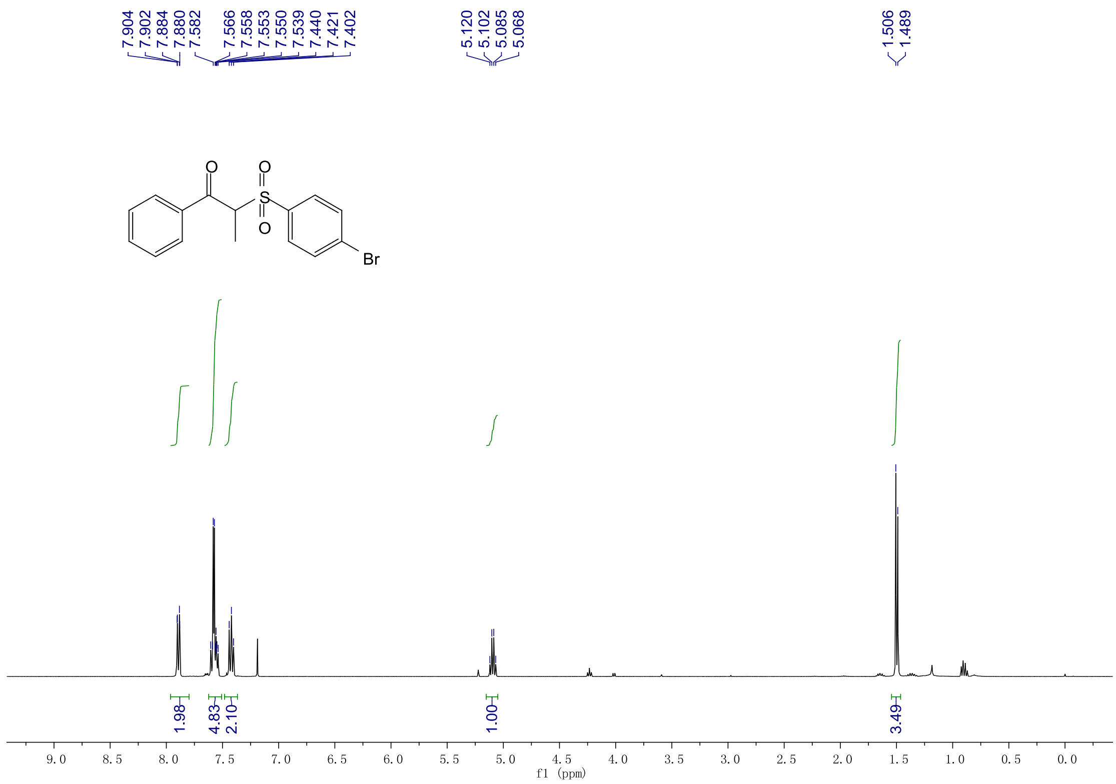

^{1}H NMR spectrum of product **3ae**

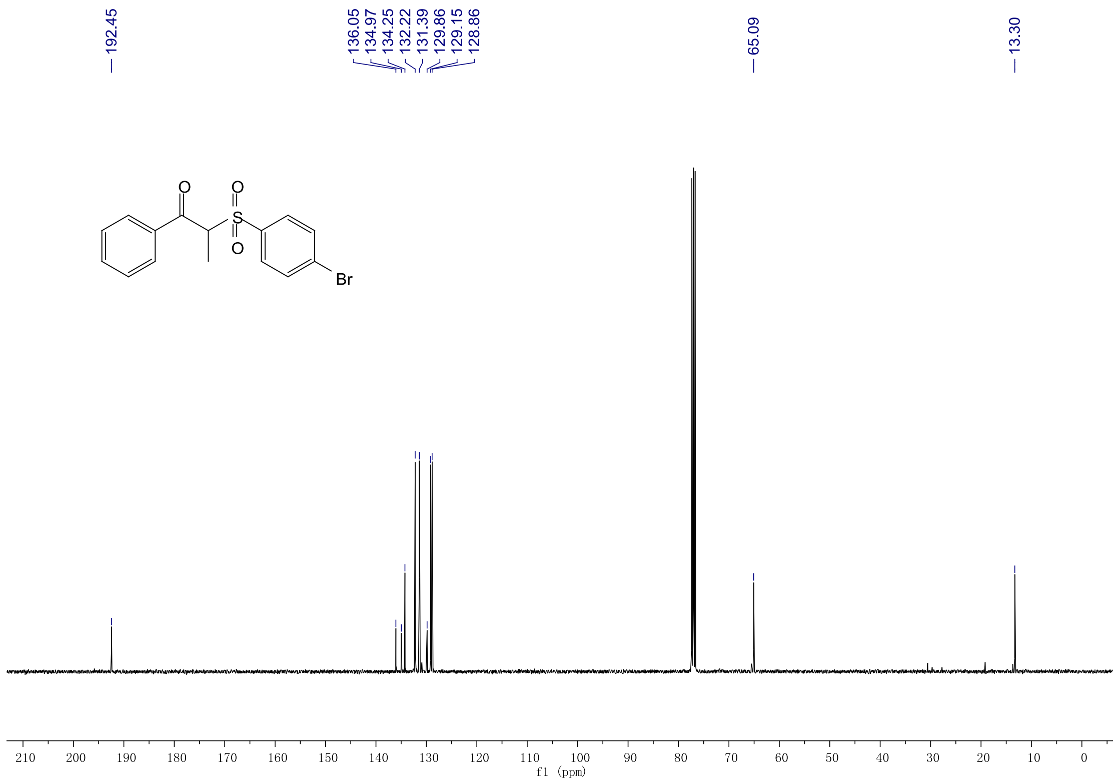

^{13}C NMR spectrum of product **3ae**

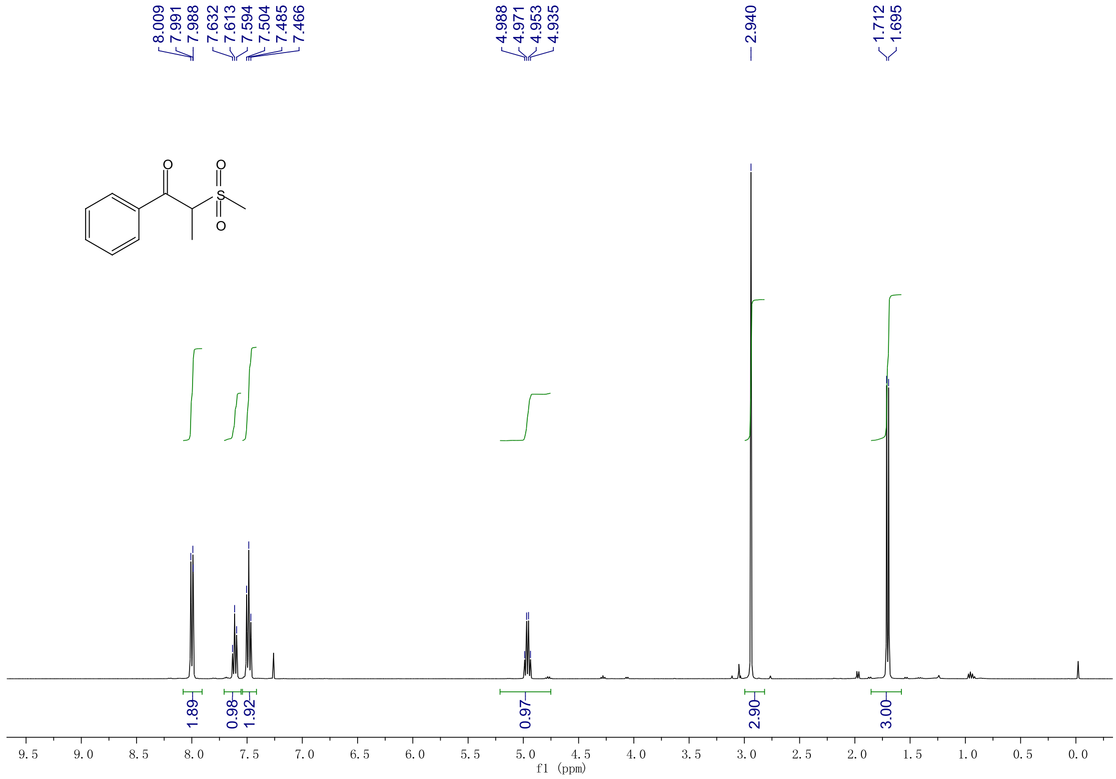

^{1}H NMR spectrum of product **3af**

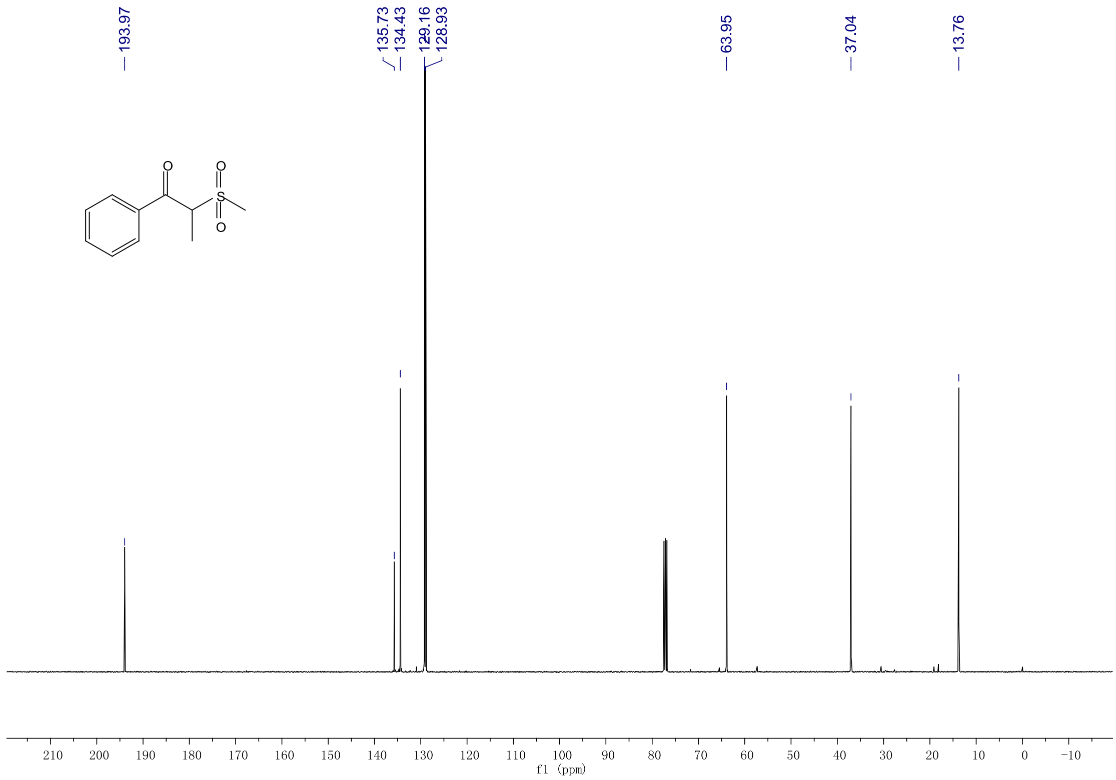

^{13}C NMR spectrum of product **3af**

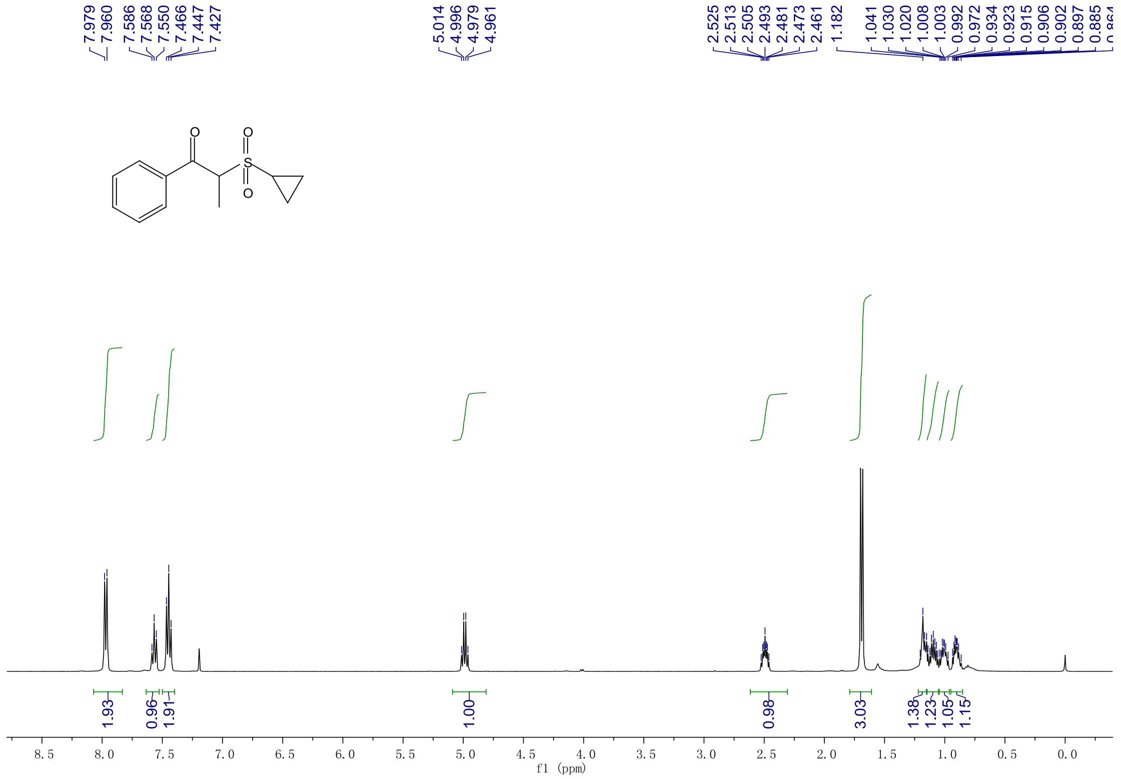

^{1}H NMR spectrum of product **3ag**

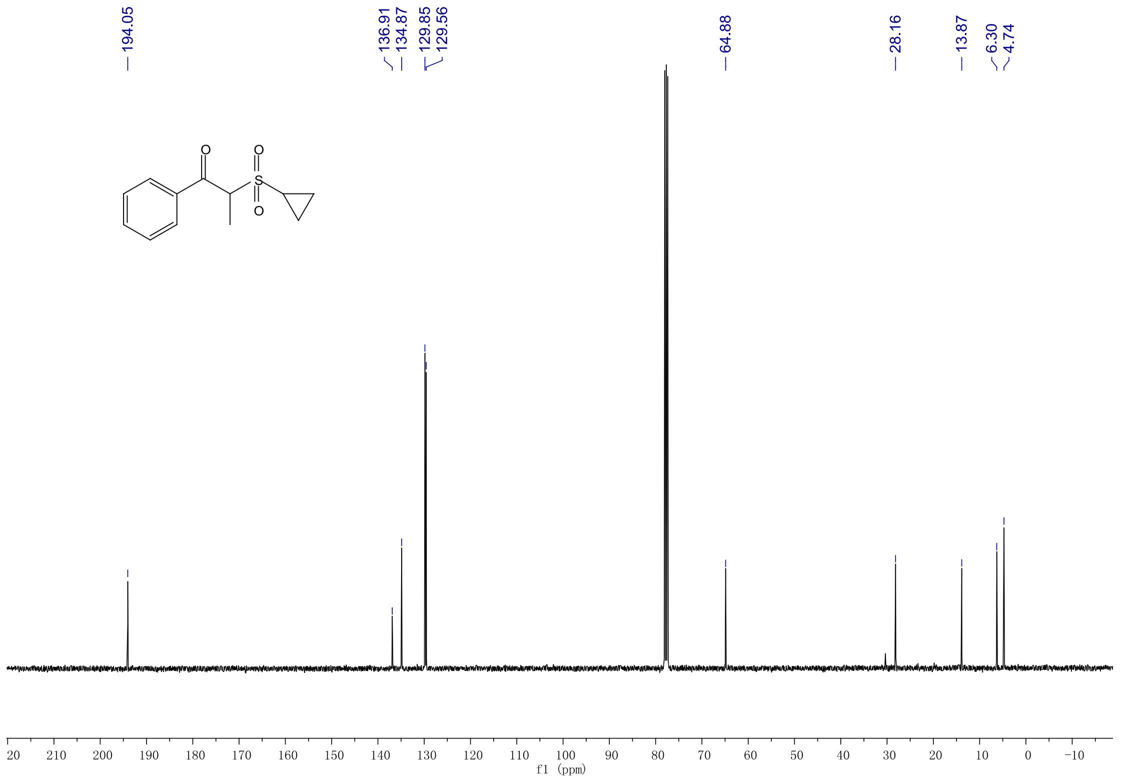

^{13}C NMR spectrum of product **3ag**

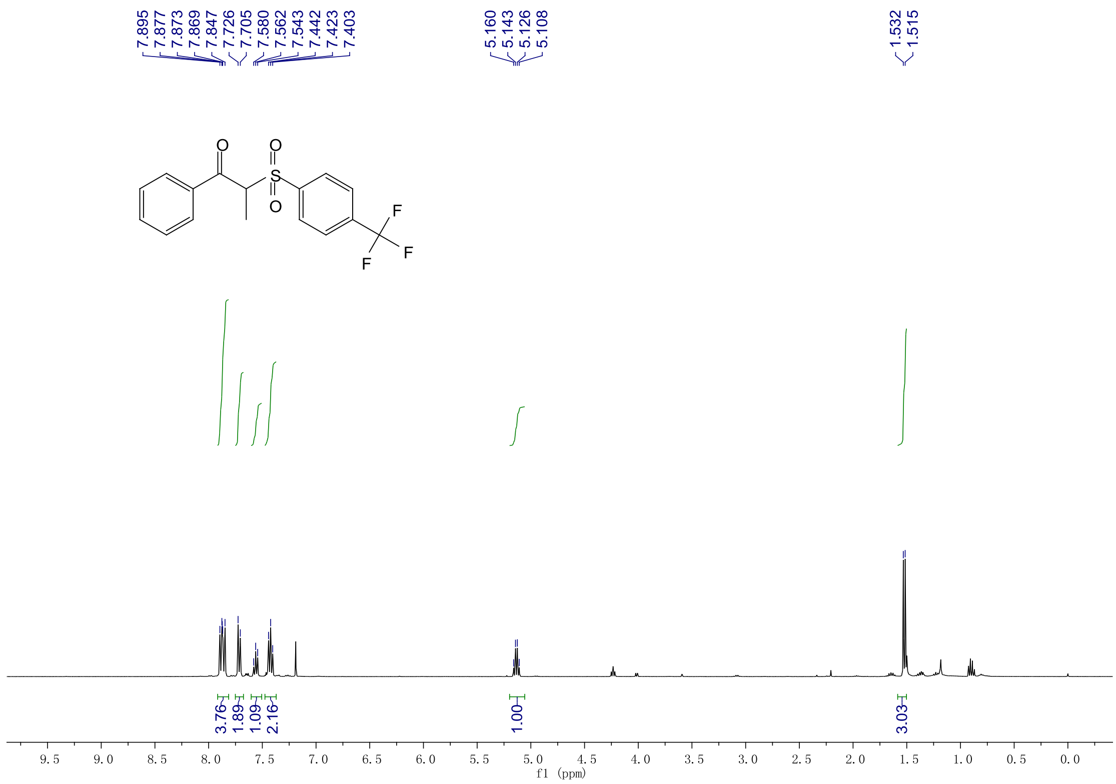

^{1}H NMR spectrum of product **3ah**

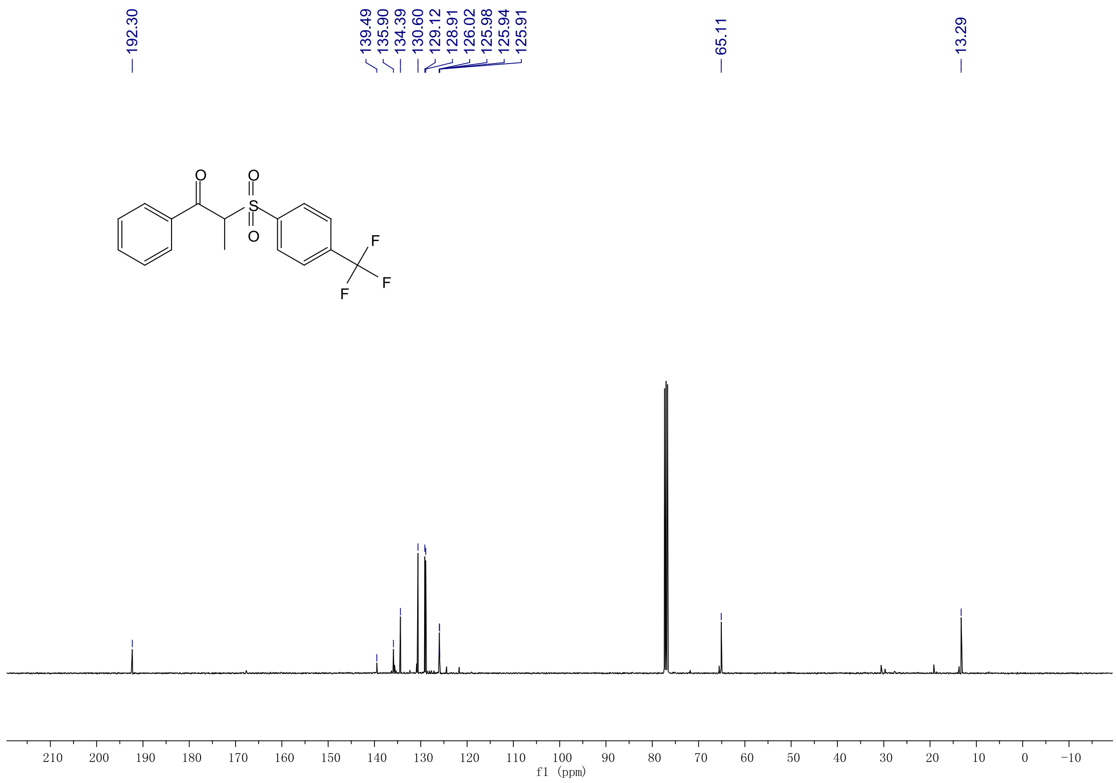

^{13}C NMR spectrum of product **3ah**

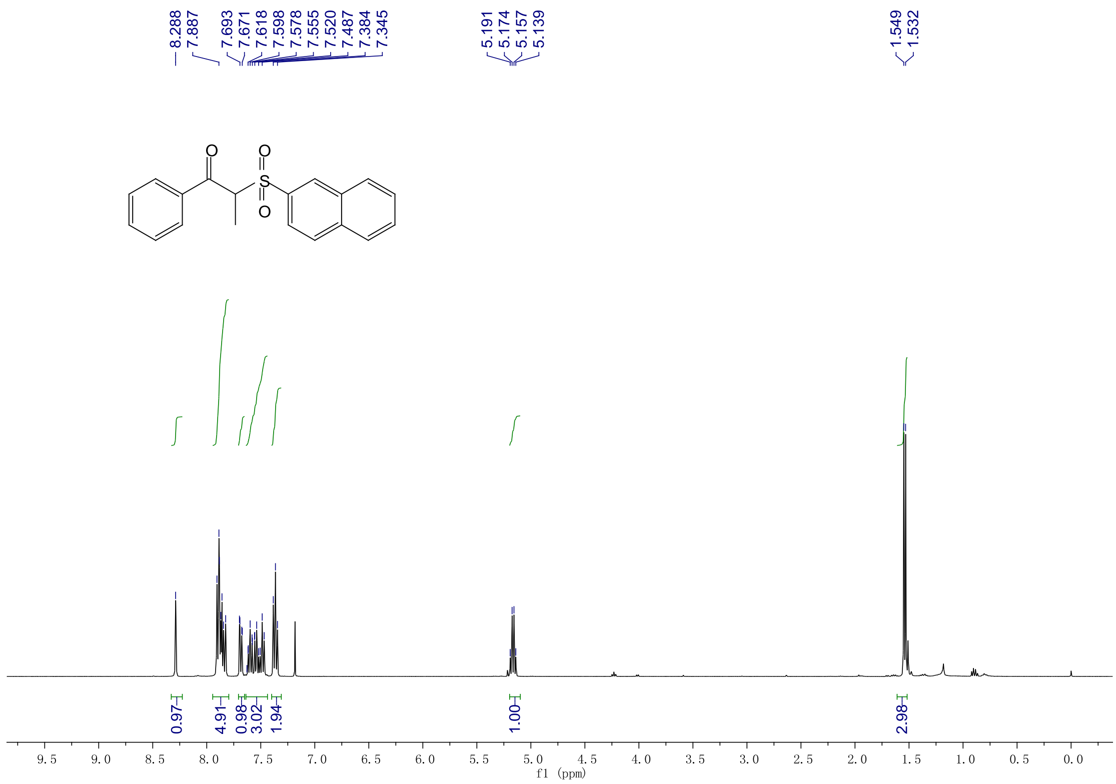

^{1}H NMR spectrum of product **3ai**

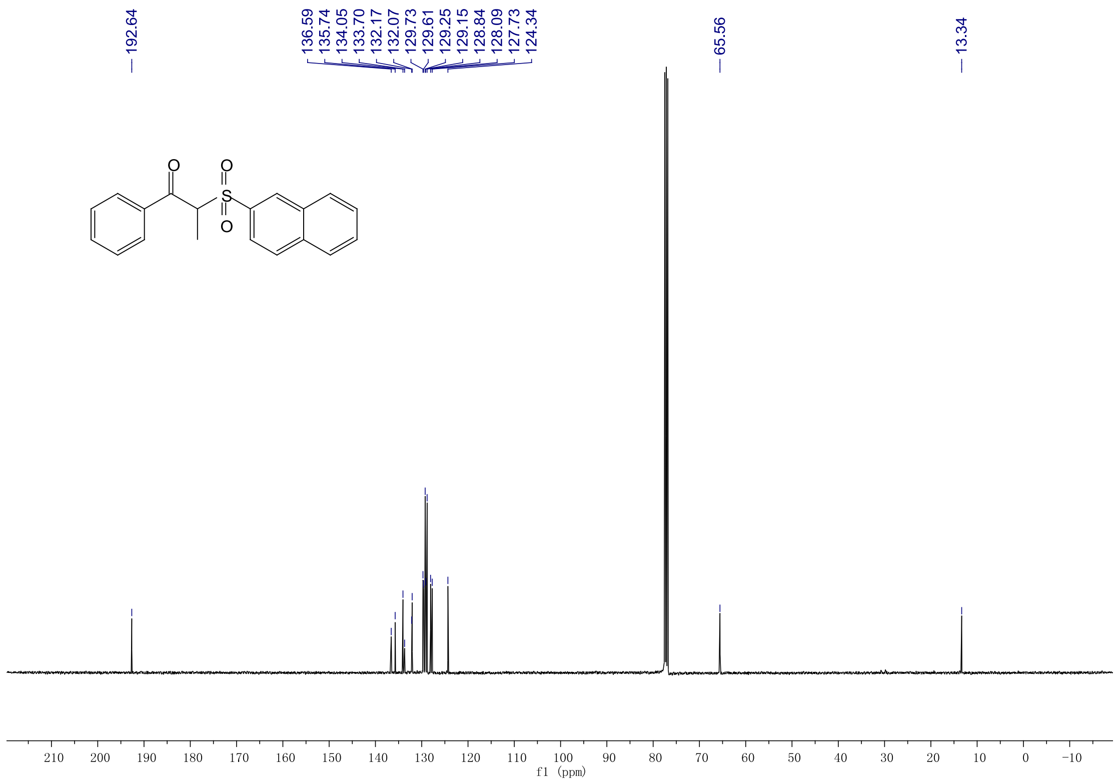

^{13}C NMR spectrum of product **3ai**

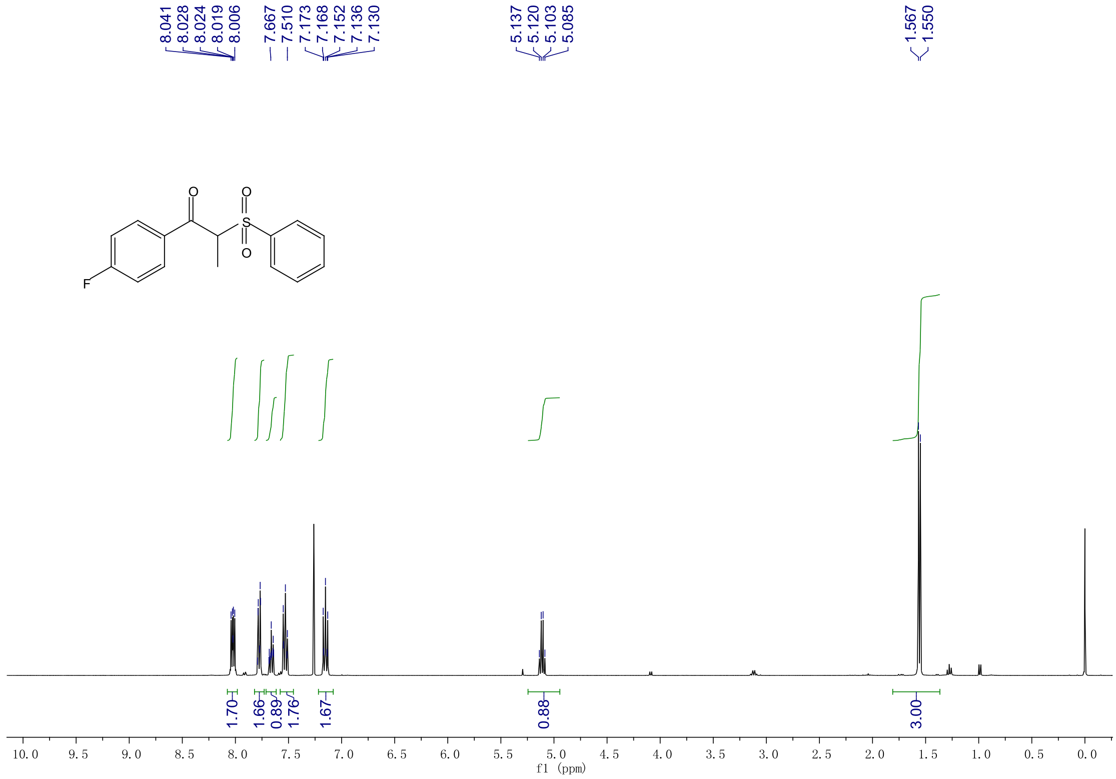

^{1}H NMR spectrum of product **3ba**

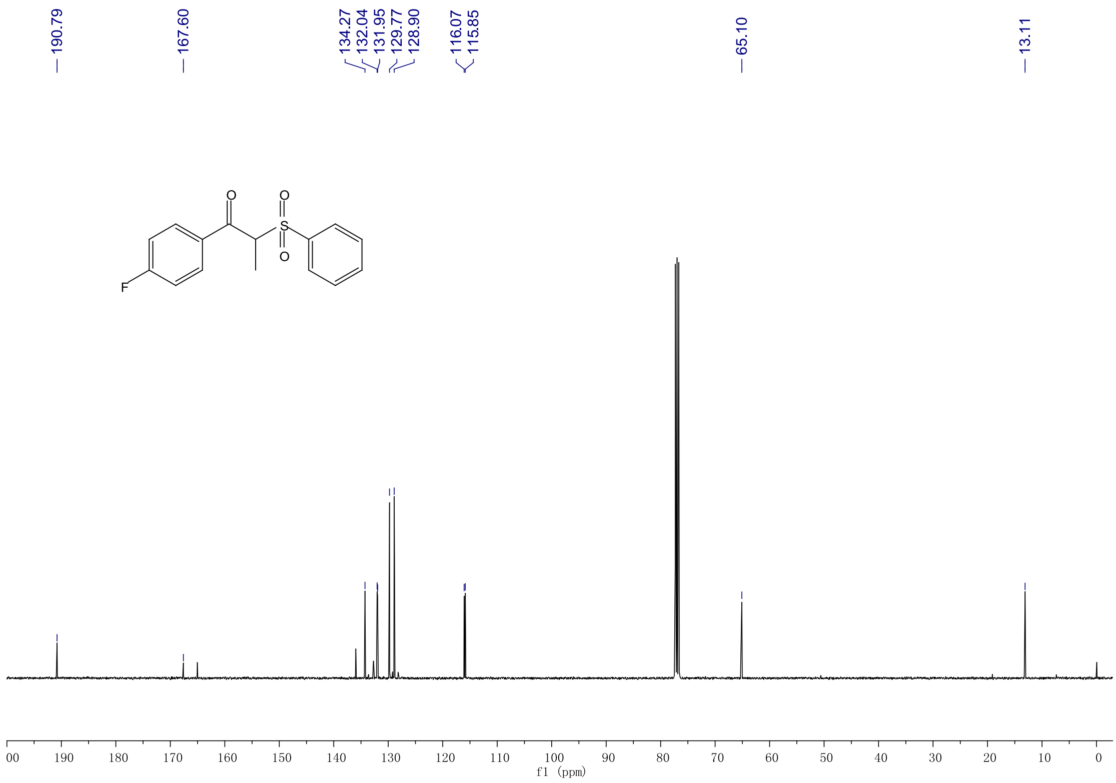

^{13}C NMR spectrum of product **3ba**

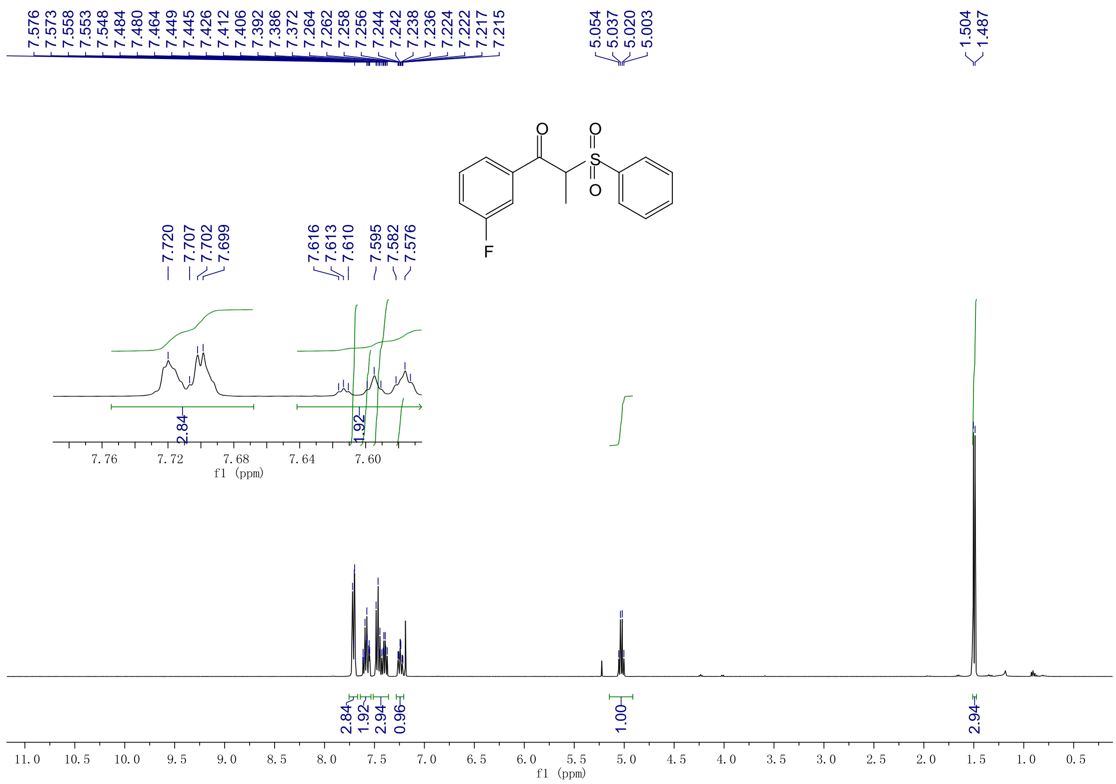

^{1}H NMR spectrum of product **3ca**

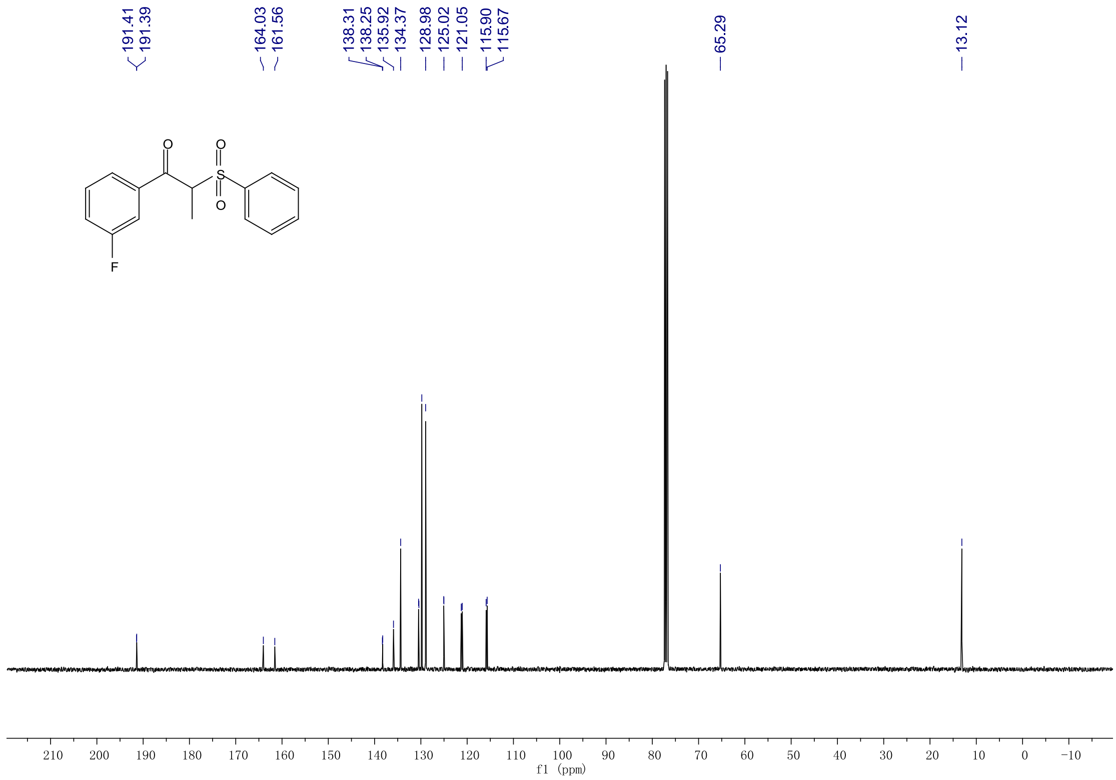

^{13}C NMR spectrum of product **3ca**

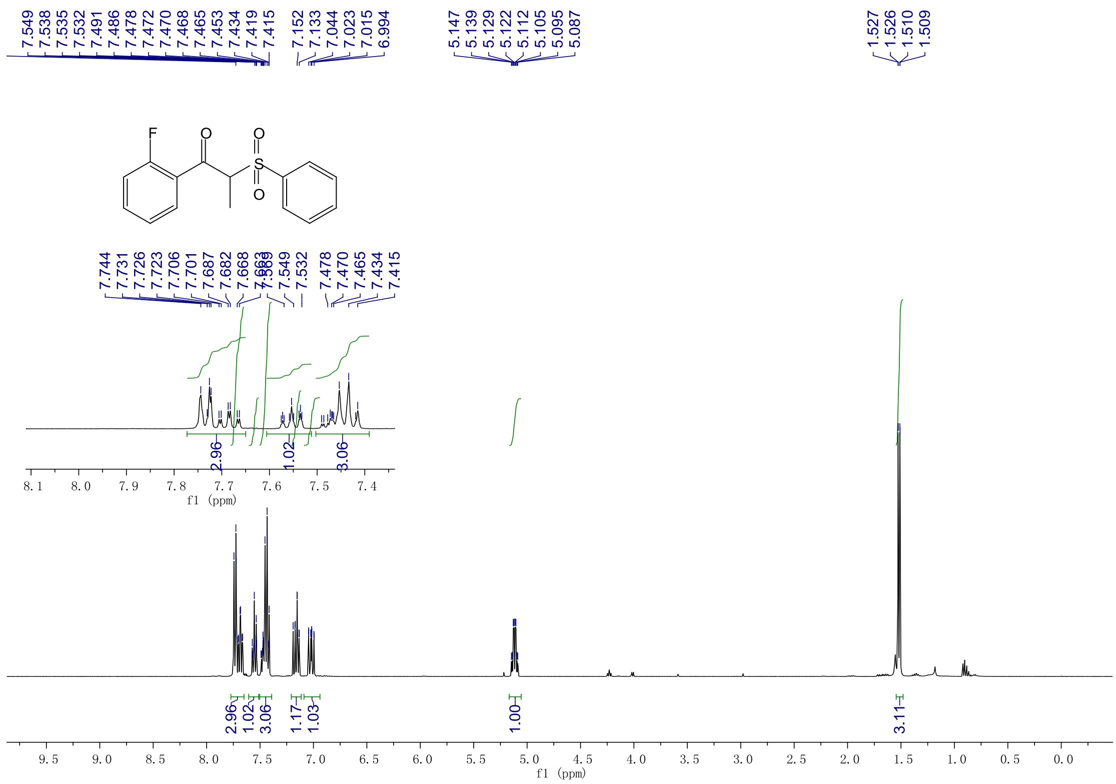

^{1}H NMR spectrum of product **3da**

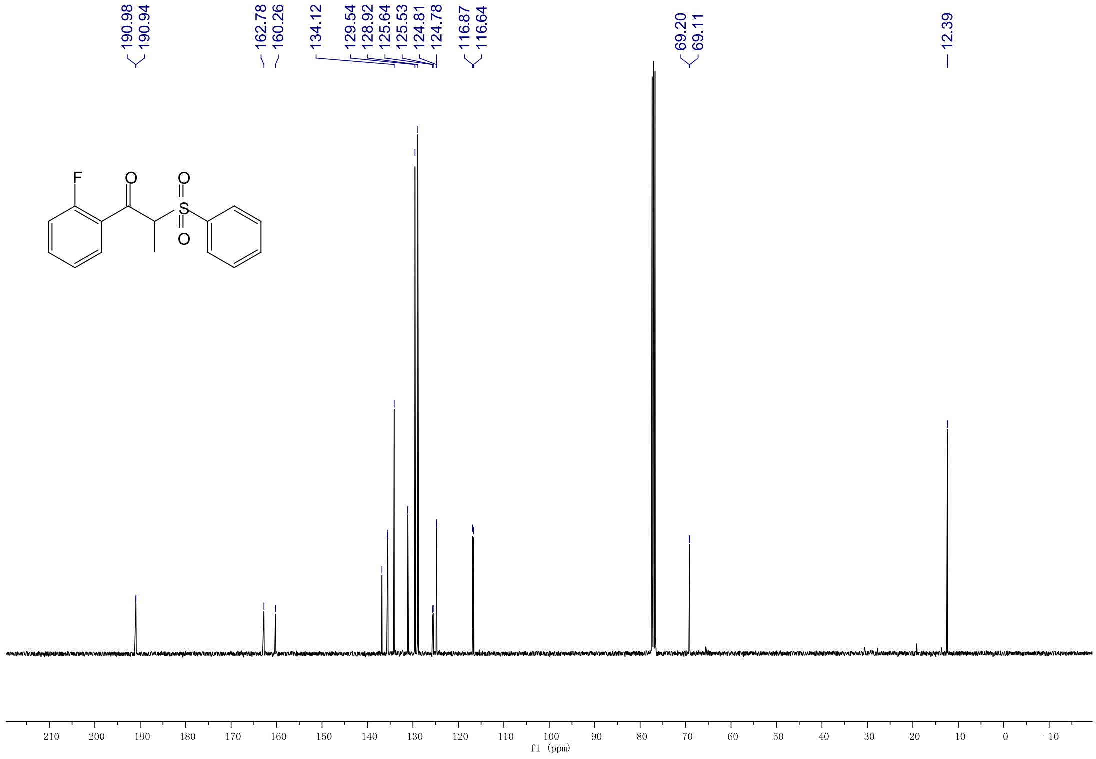

^{13}C NMR spectrum of product **3da**

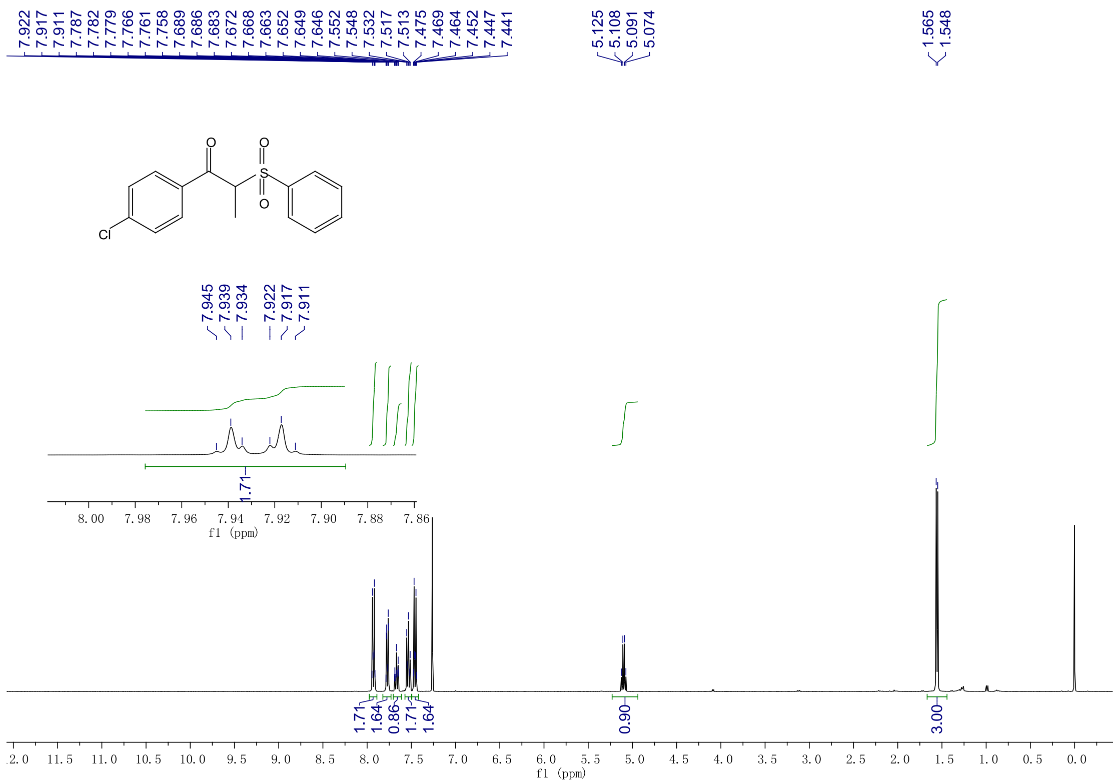

^{1}H NMR spectrum of product **3ea**

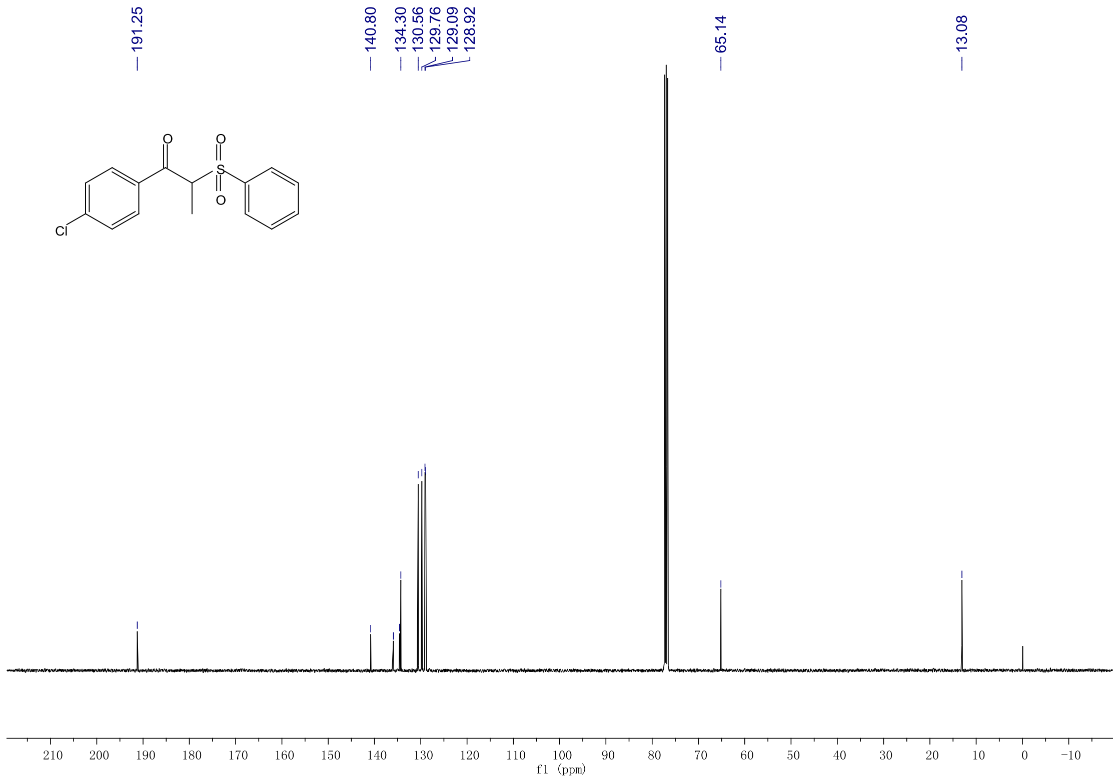

^{13}C NMR spectrum of product **3ea**

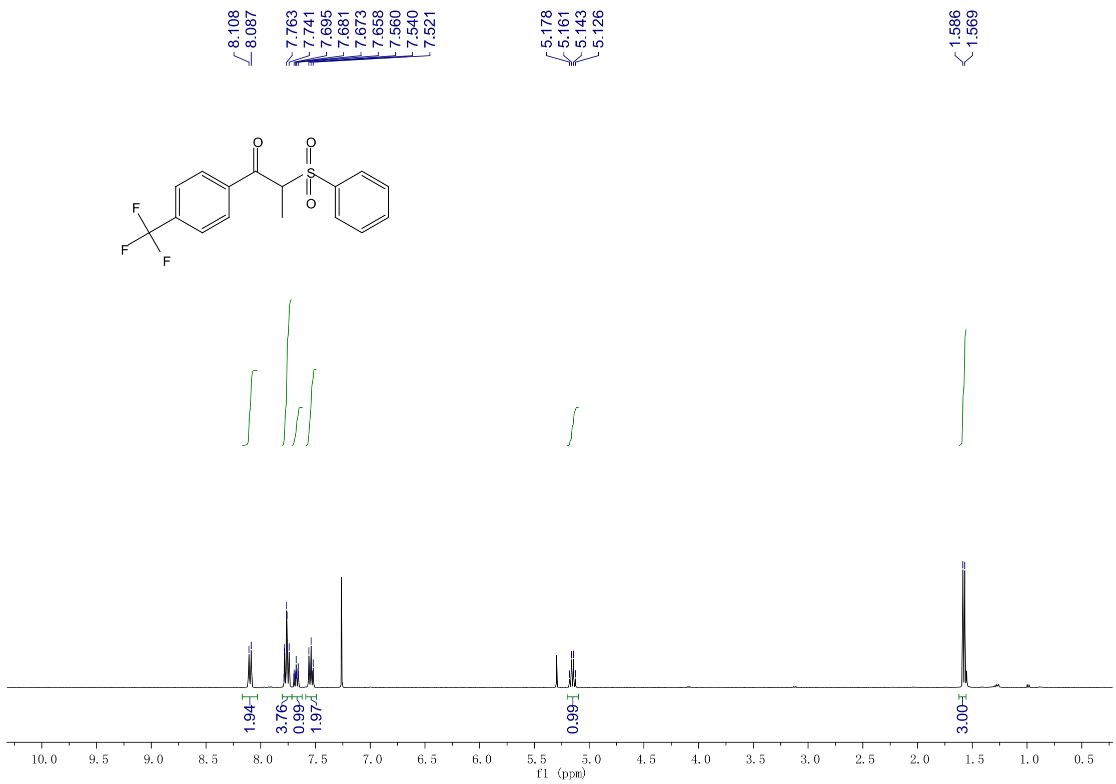

^{1}H NMR spectrum of product **3fa**

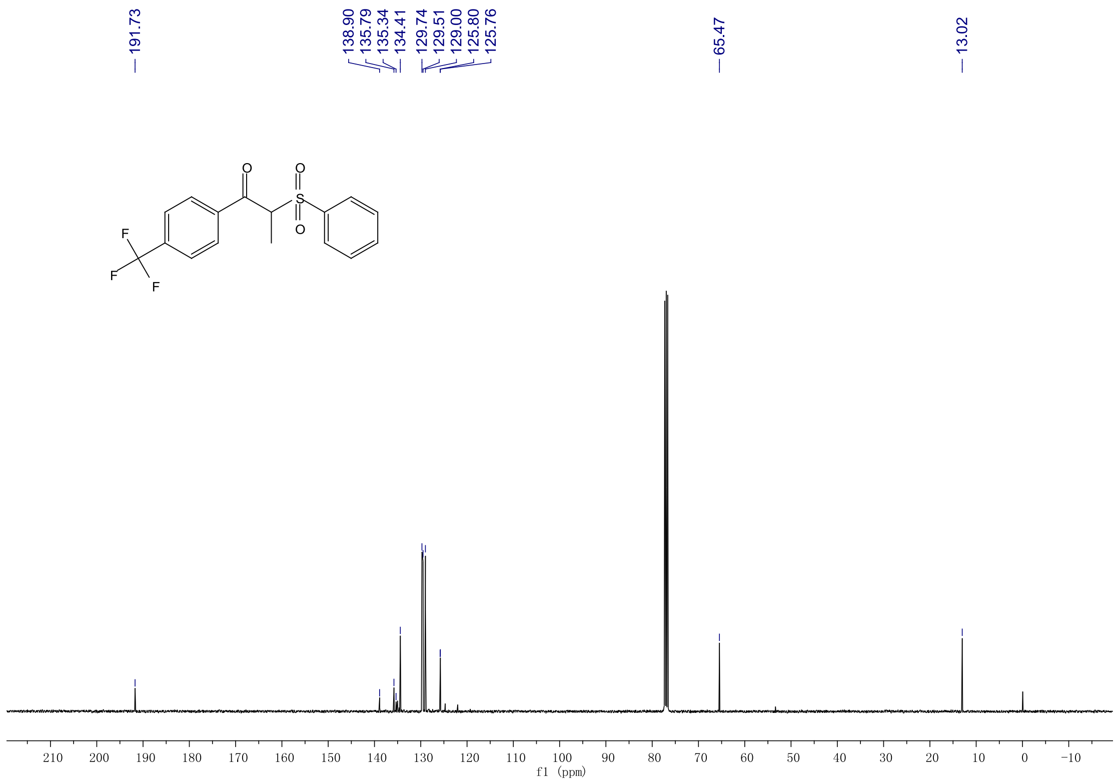

^{13}C NMR spectrum of product **3fa**

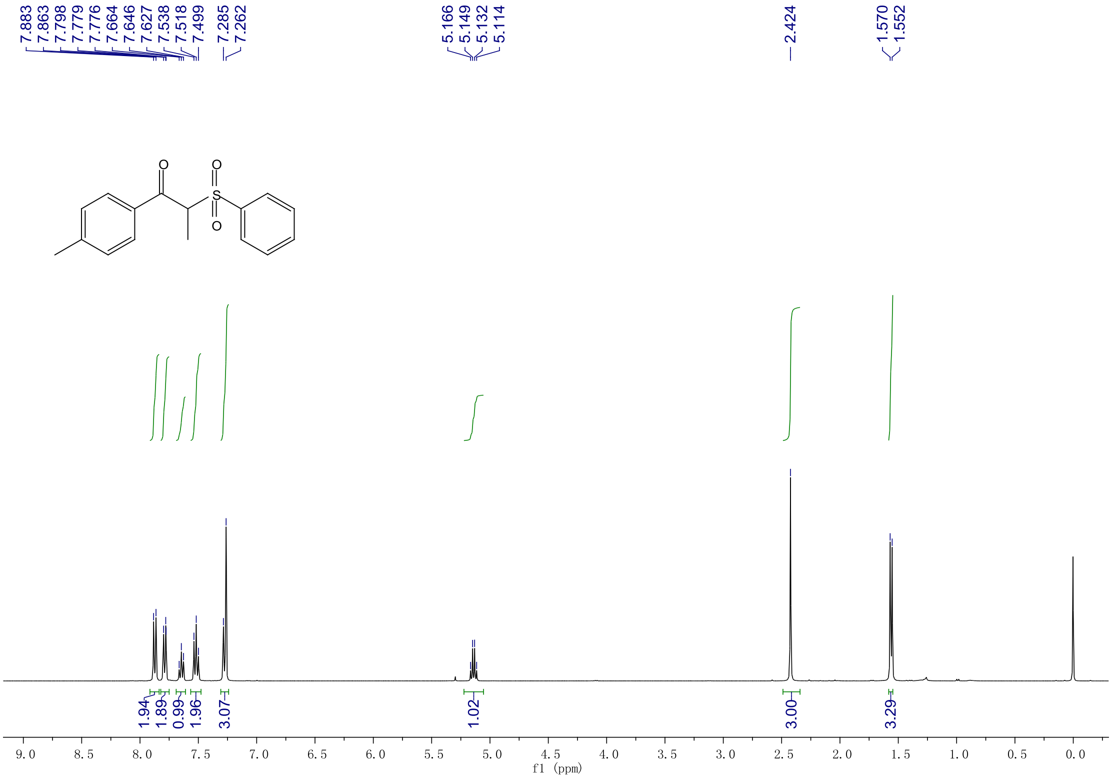

^{1}H NMR spectrum of product **3ga**

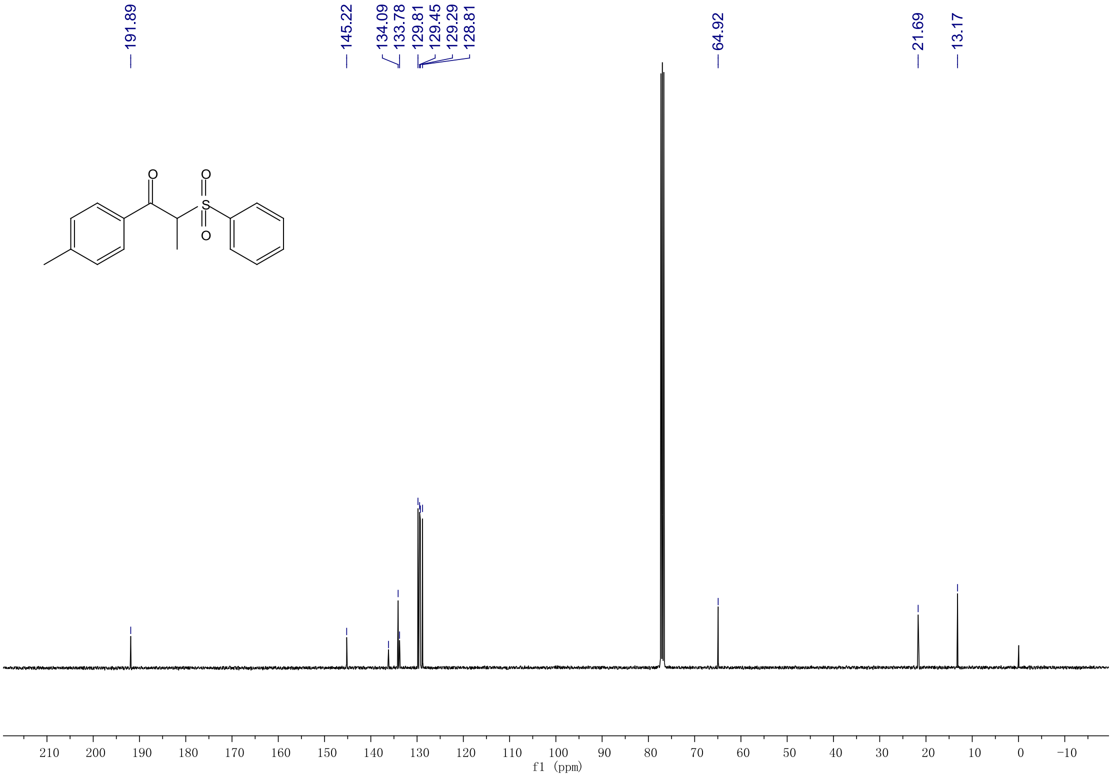

^{13}C NMR spectrum of product **3ga**

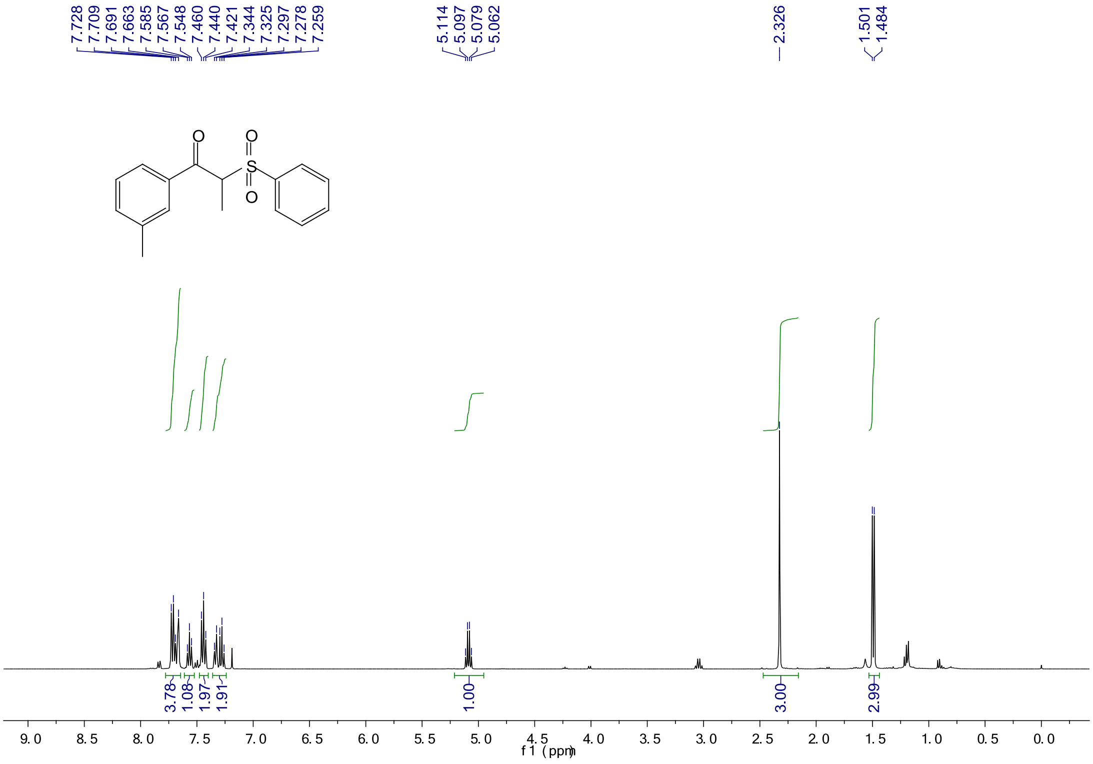

^{1}H NMR spectrum of product **3ha**

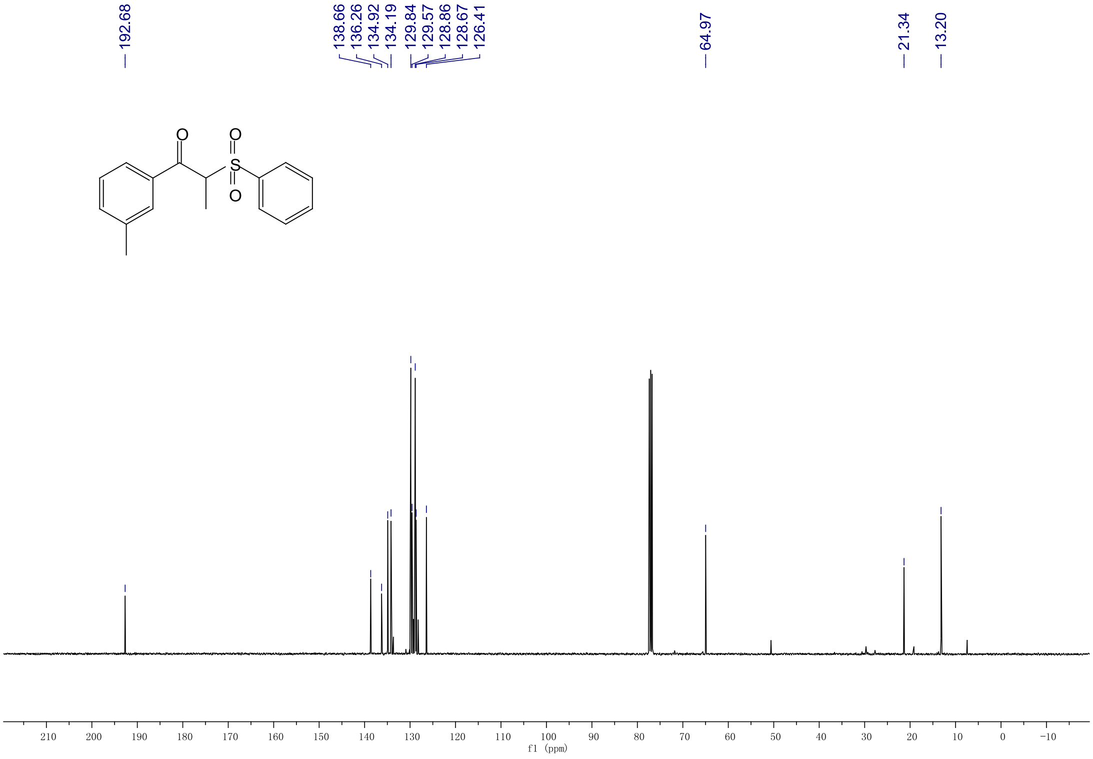

^{13}C NMR spectrum of product **3ha**

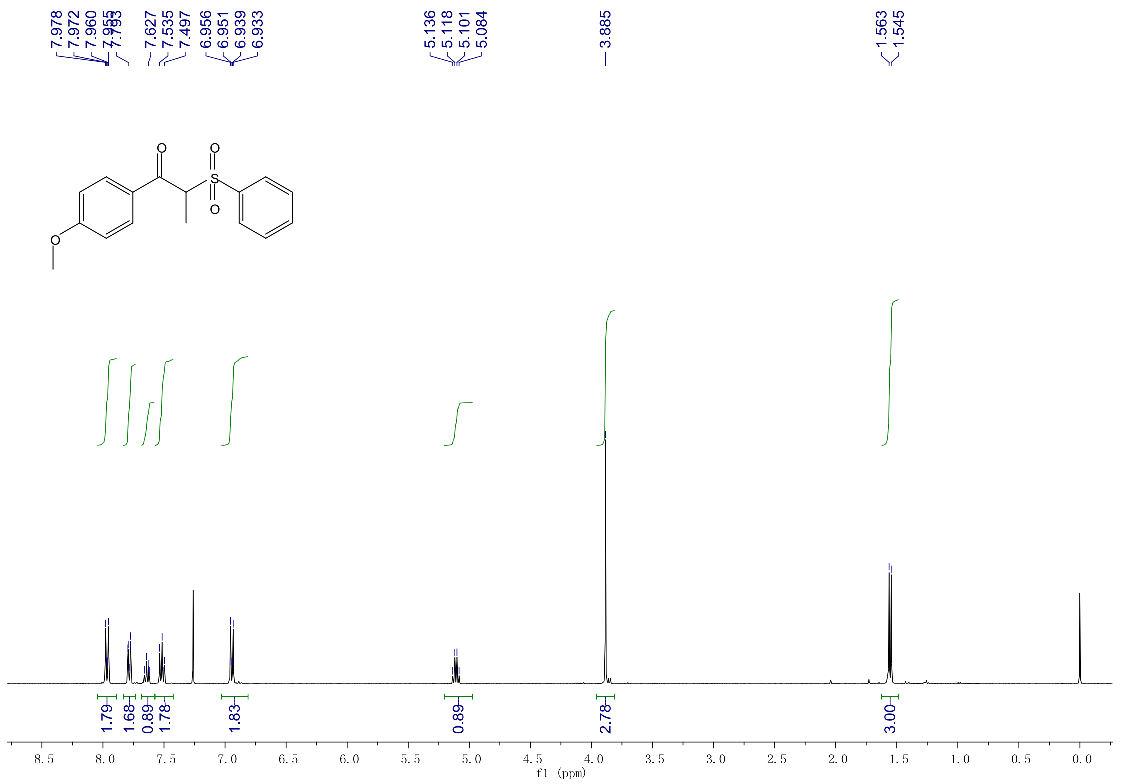

^{1}H NMR spectrum of product **3ia**

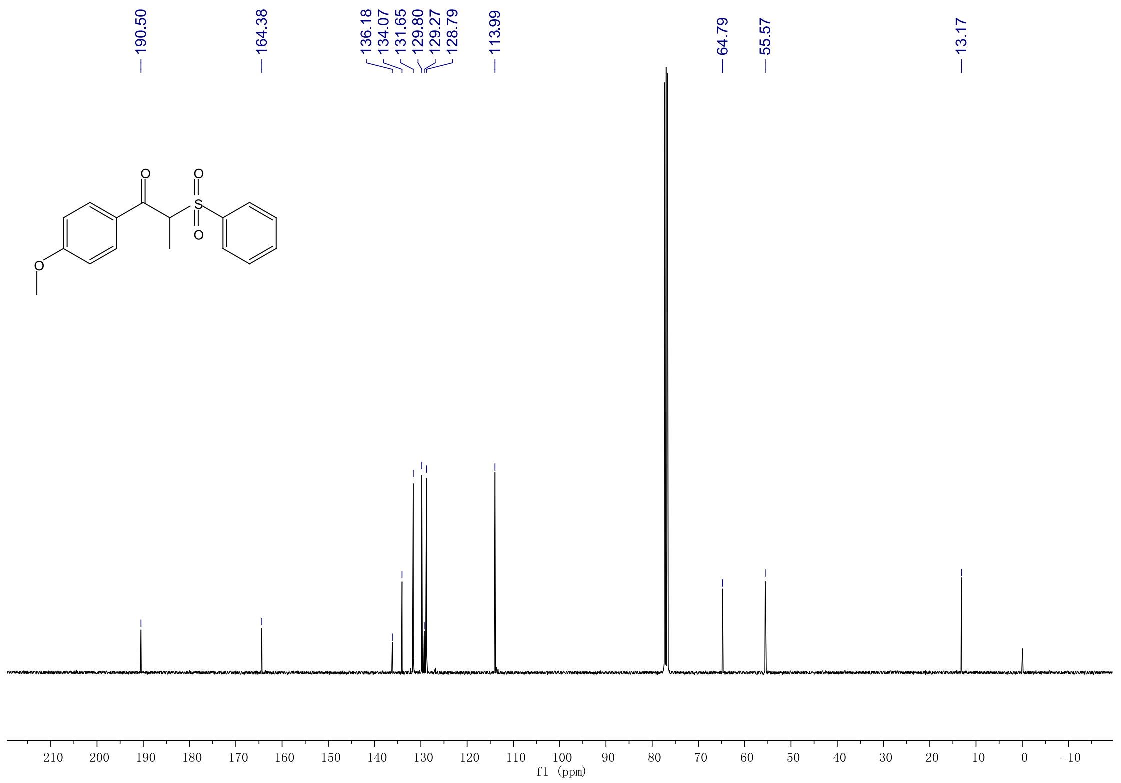

^{13}C NMR spectrum of product **3ia**

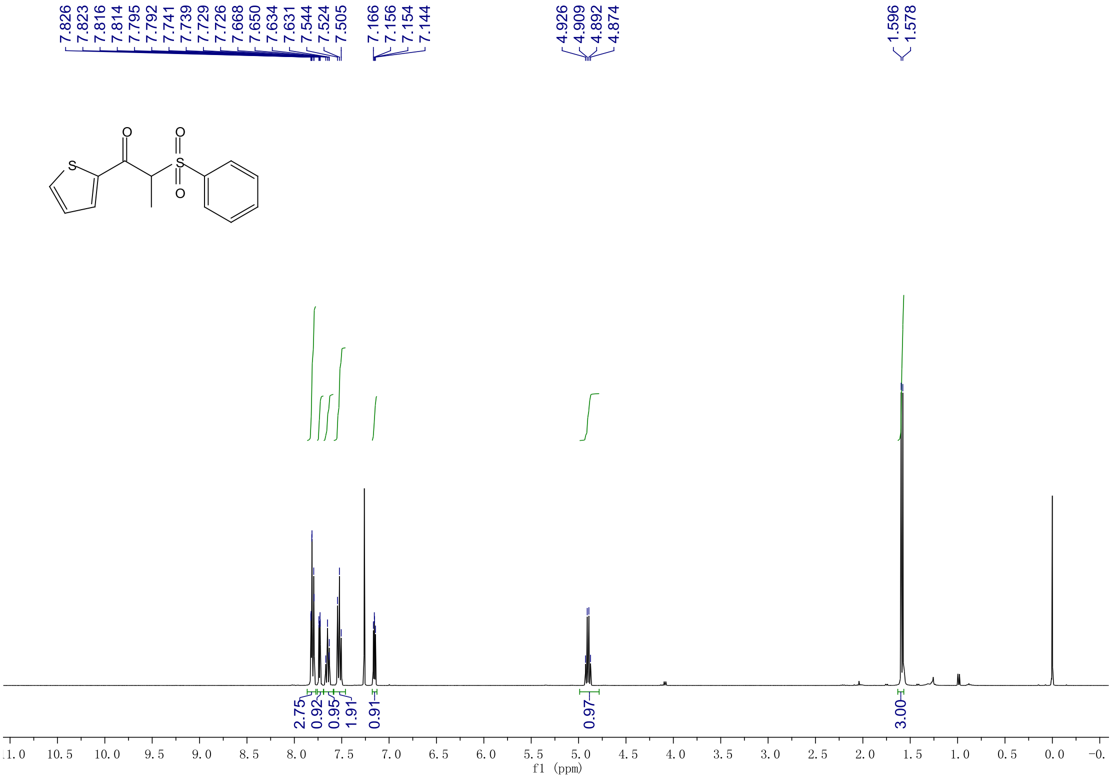

^{1}H NMR spectrum of product **3ja**

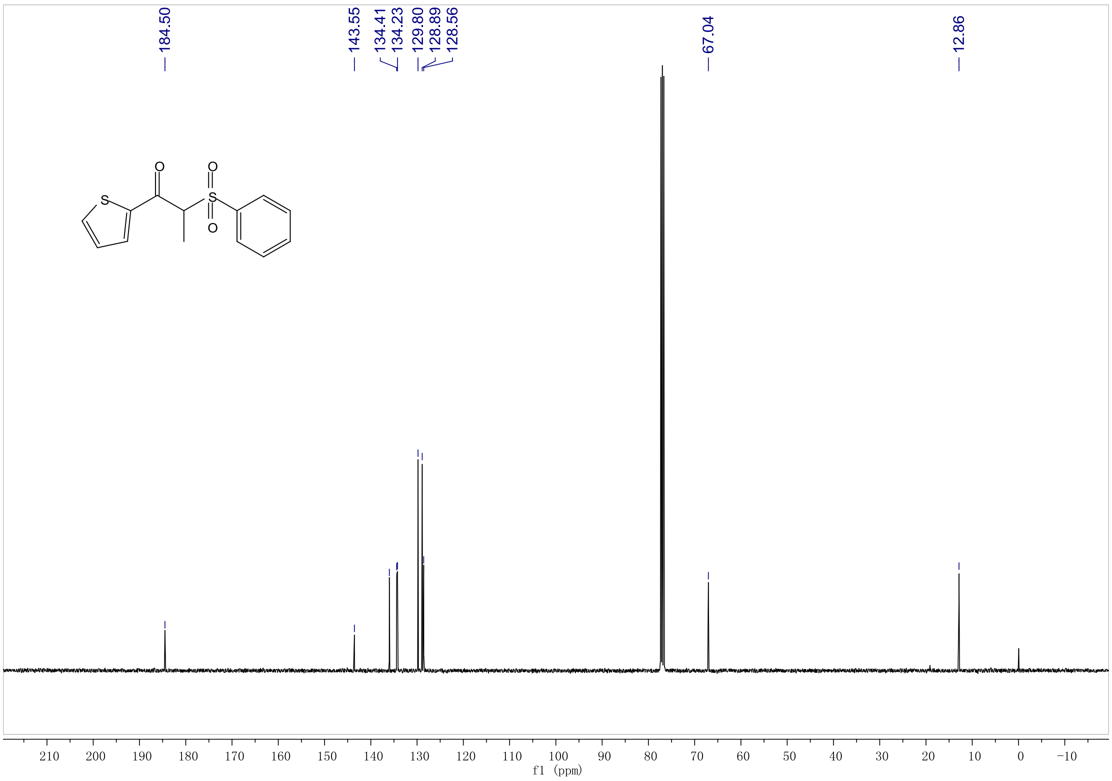

^{13}C NMR spectrum of product **3ja**

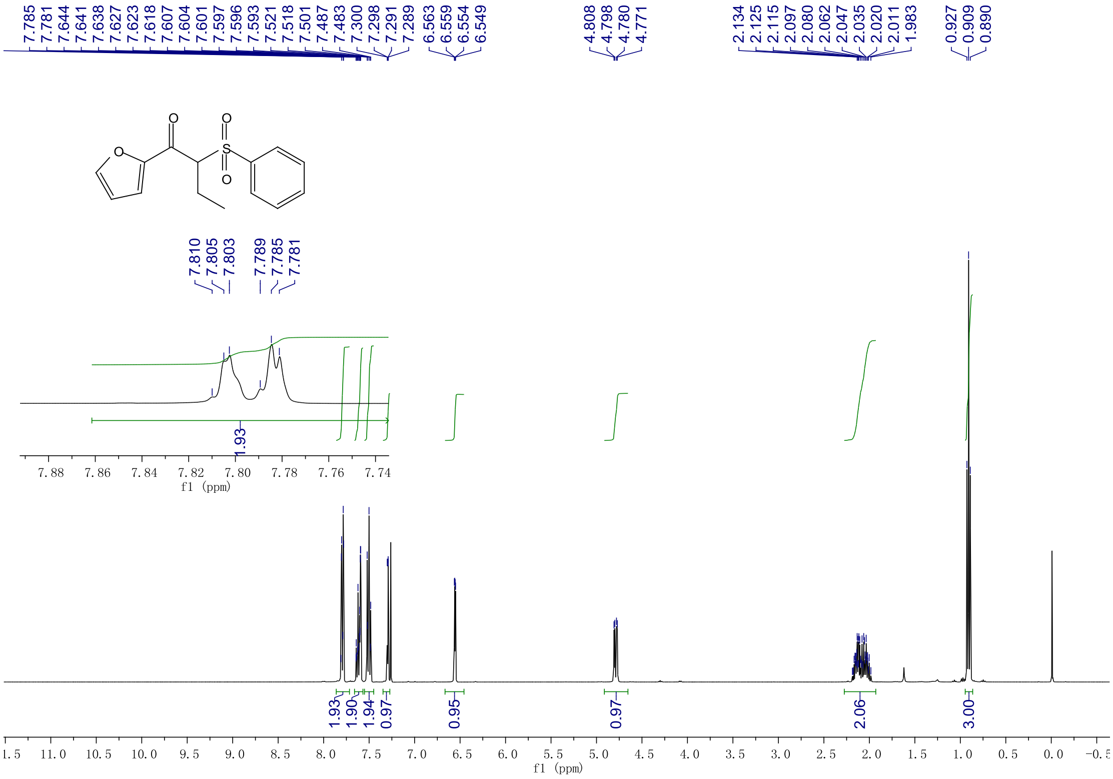

^{1}H NMR spectrum of product **3ka**

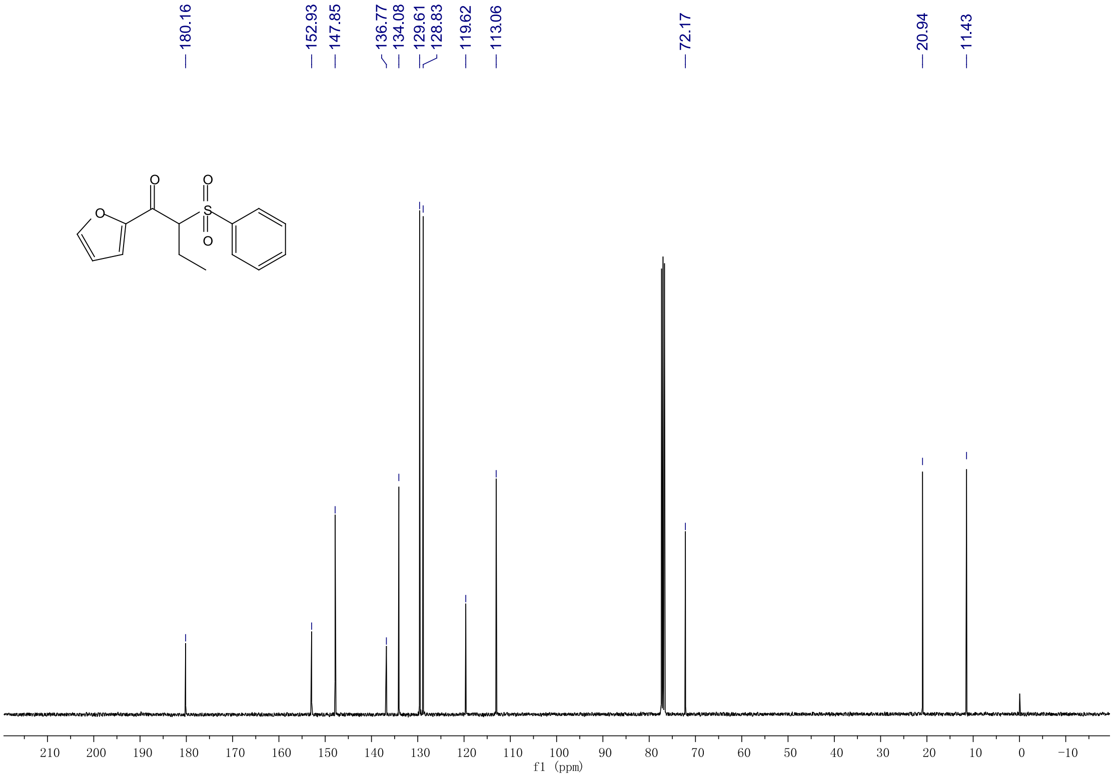

^{13}C NMR spectrum of product **3ka**

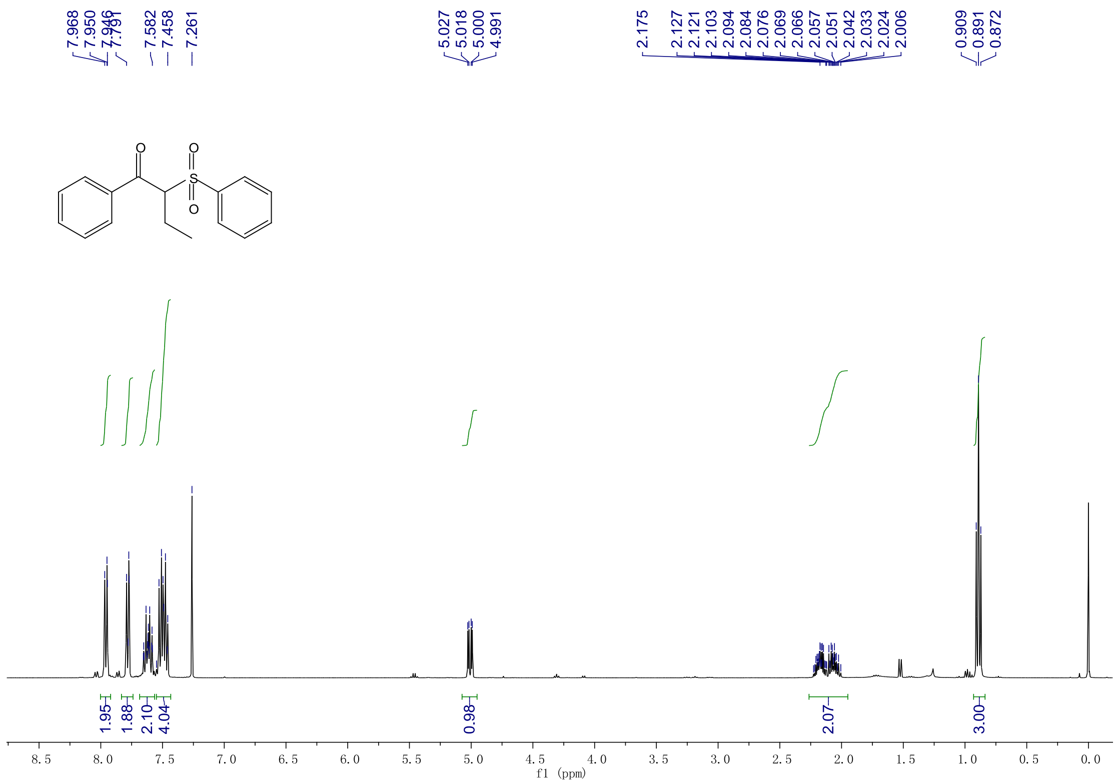

^{1}H NMR spectrum of product **3la**

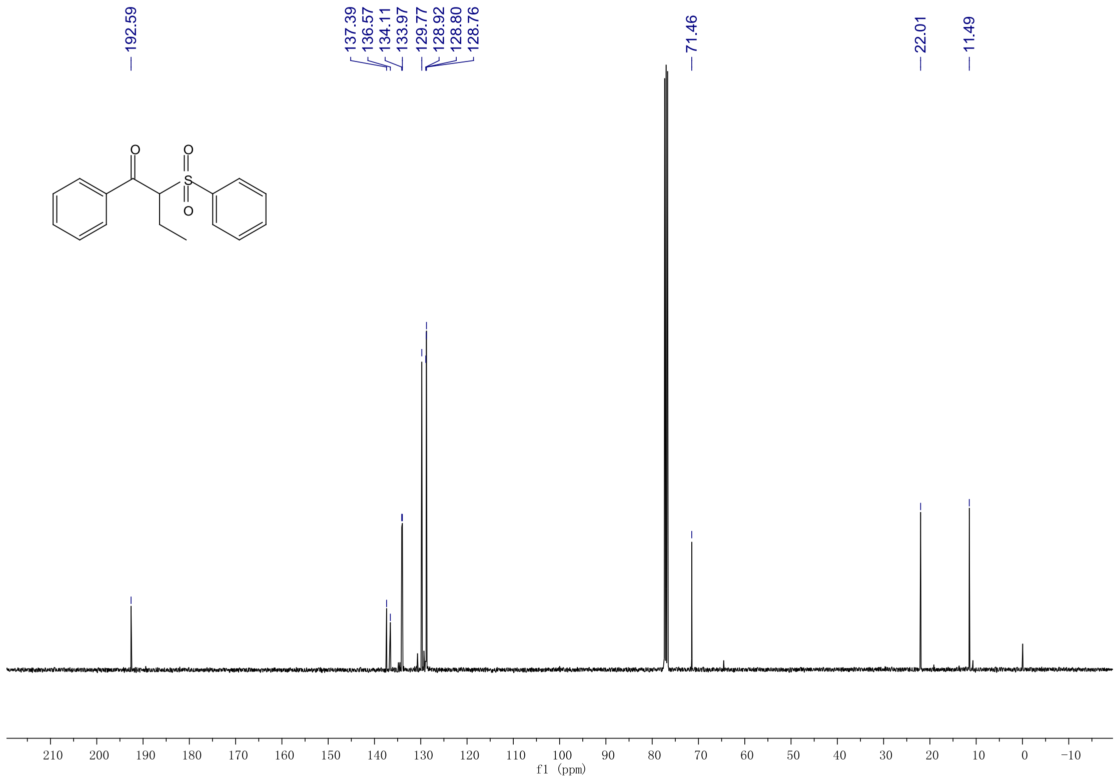

^{13}C NMR spectrum of product **3la**

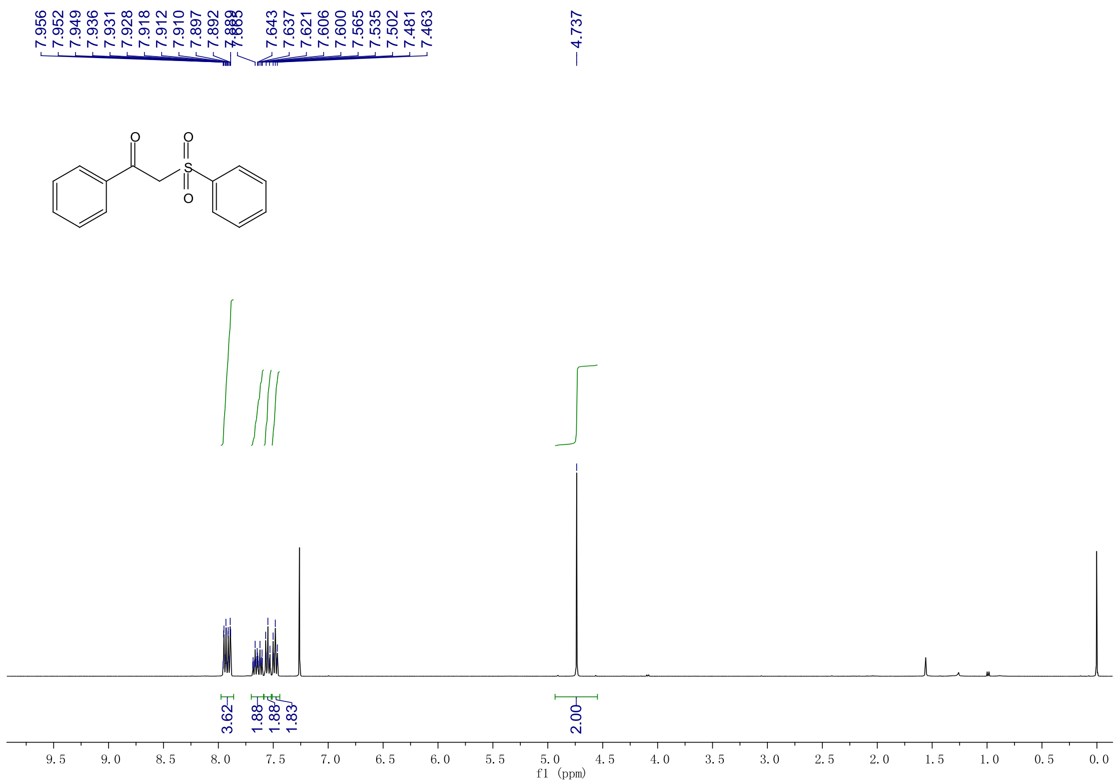

^{1}H NMR spectrum of product **3ma**

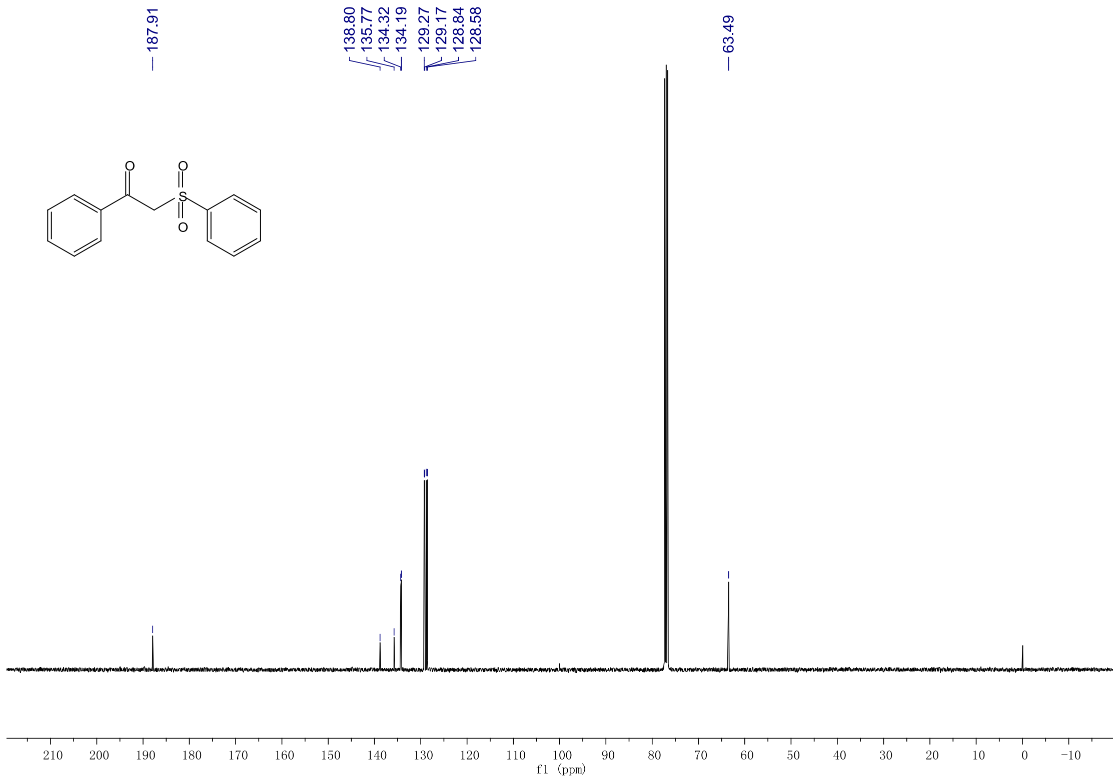

^{13}C NMR spectrum of product **3ma**

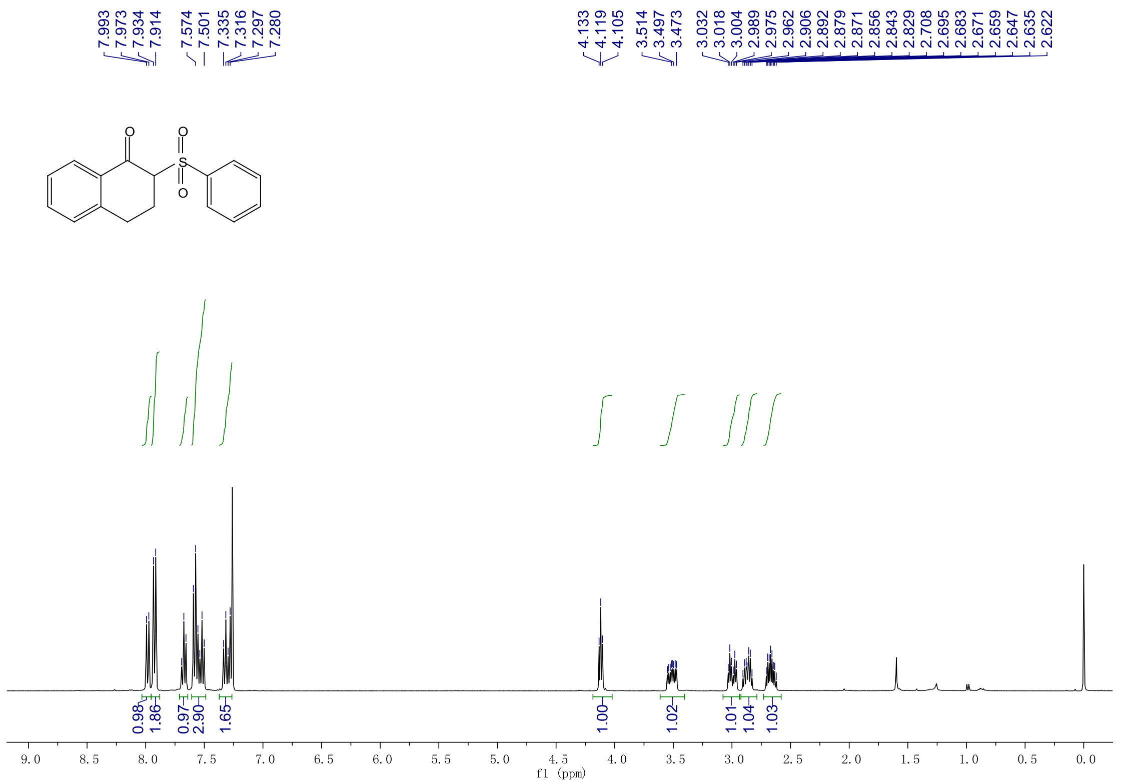

^{1}H NMR spectrum of product **3na**

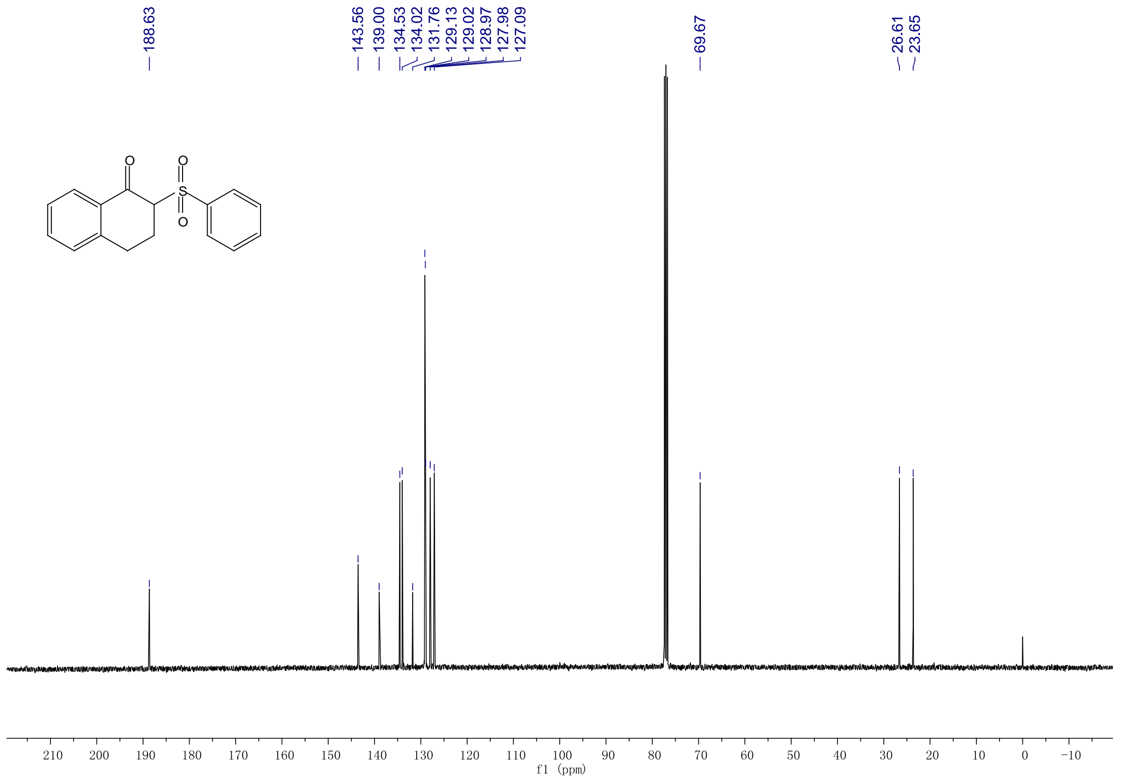

^{13}C NMR spectrum of product **3na**

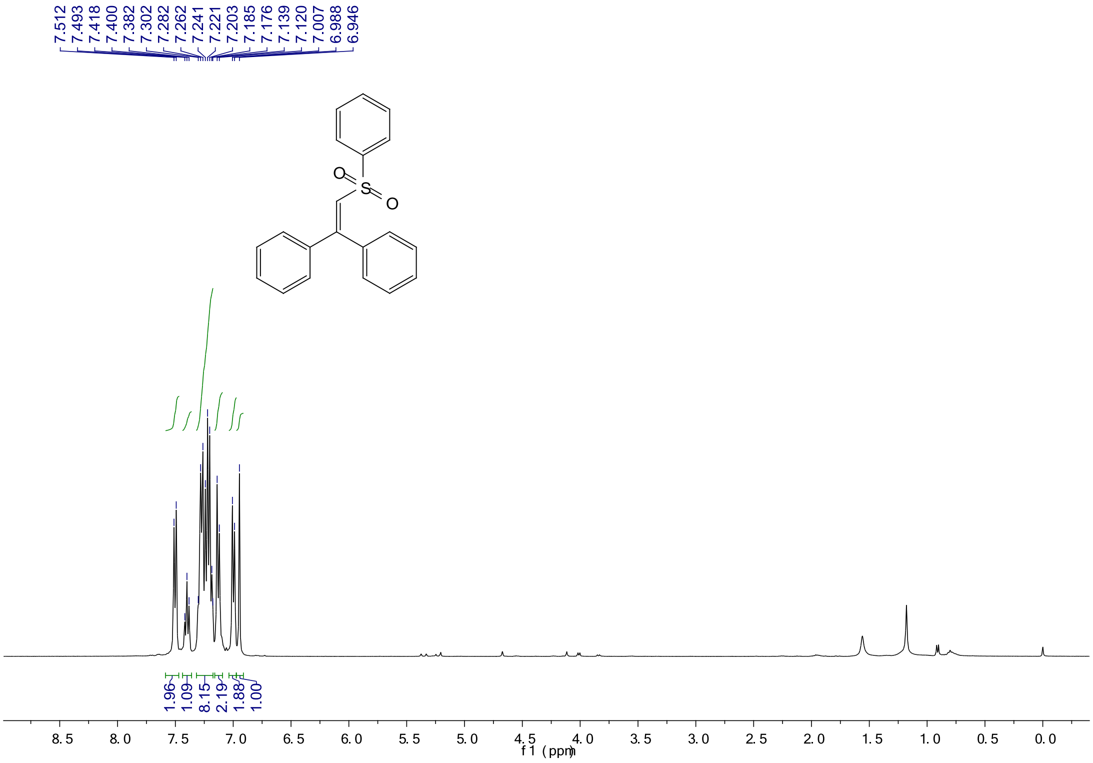

^{1}H NMR spectrum of product **4a**

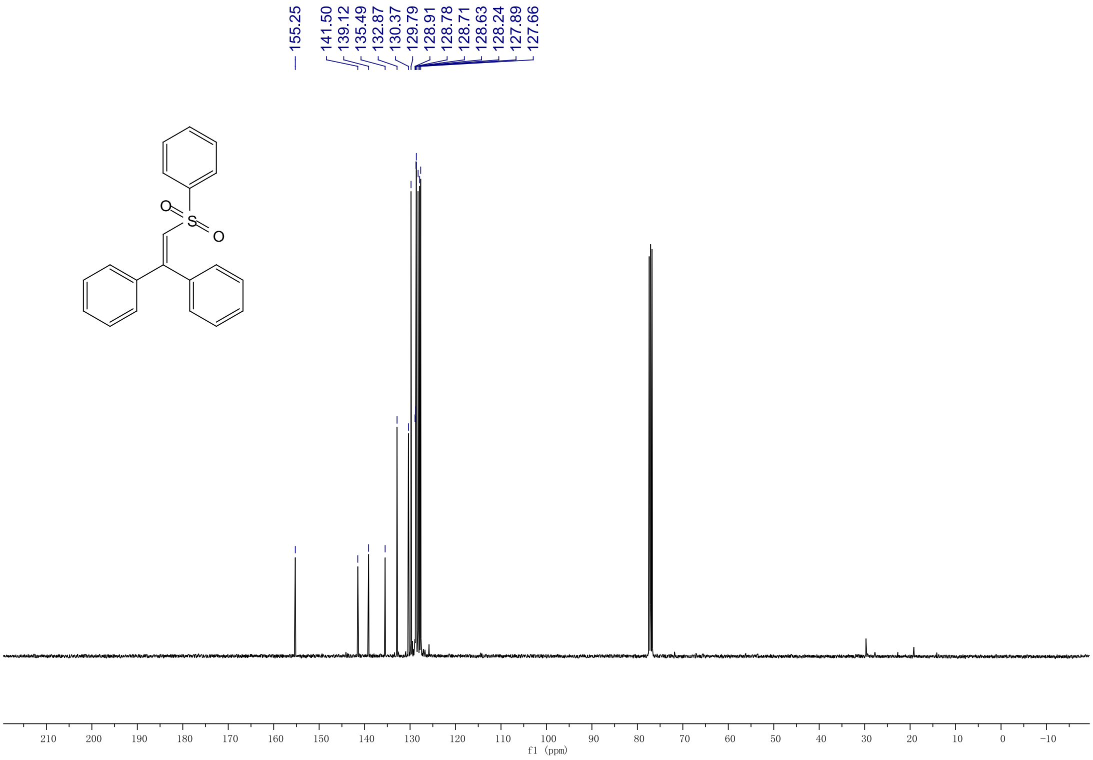

^{13}C NMR spectrum of product **4a**

6. Copies of HRMS Spectra of All Products

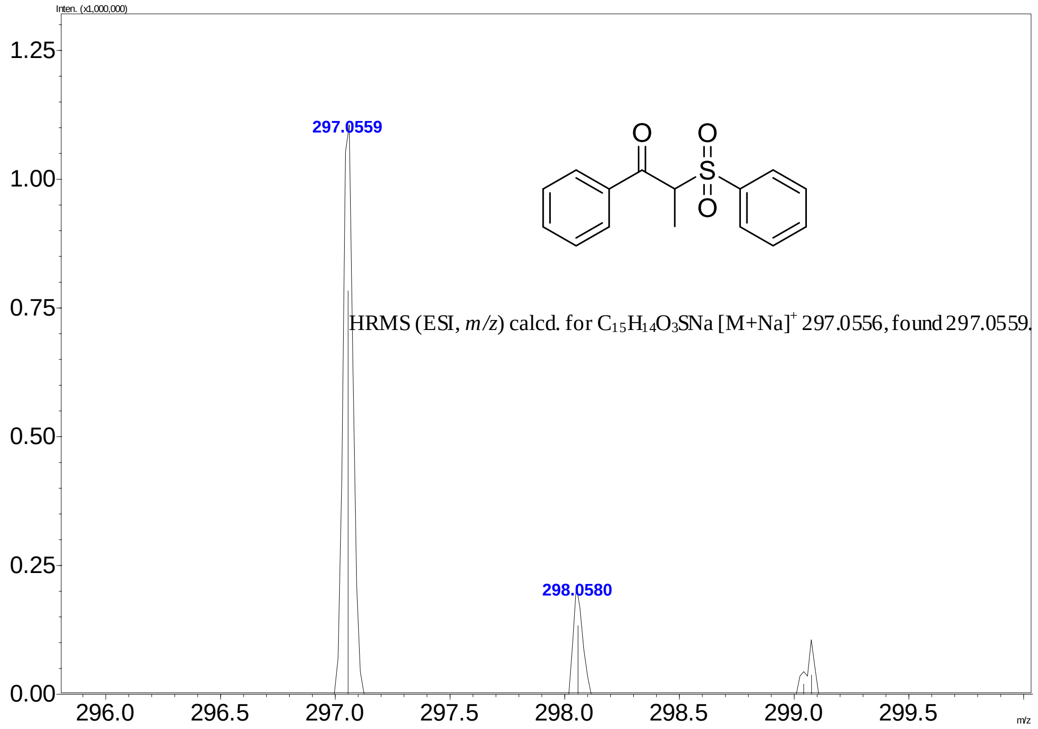

HRMS spectrum of product **3aa**

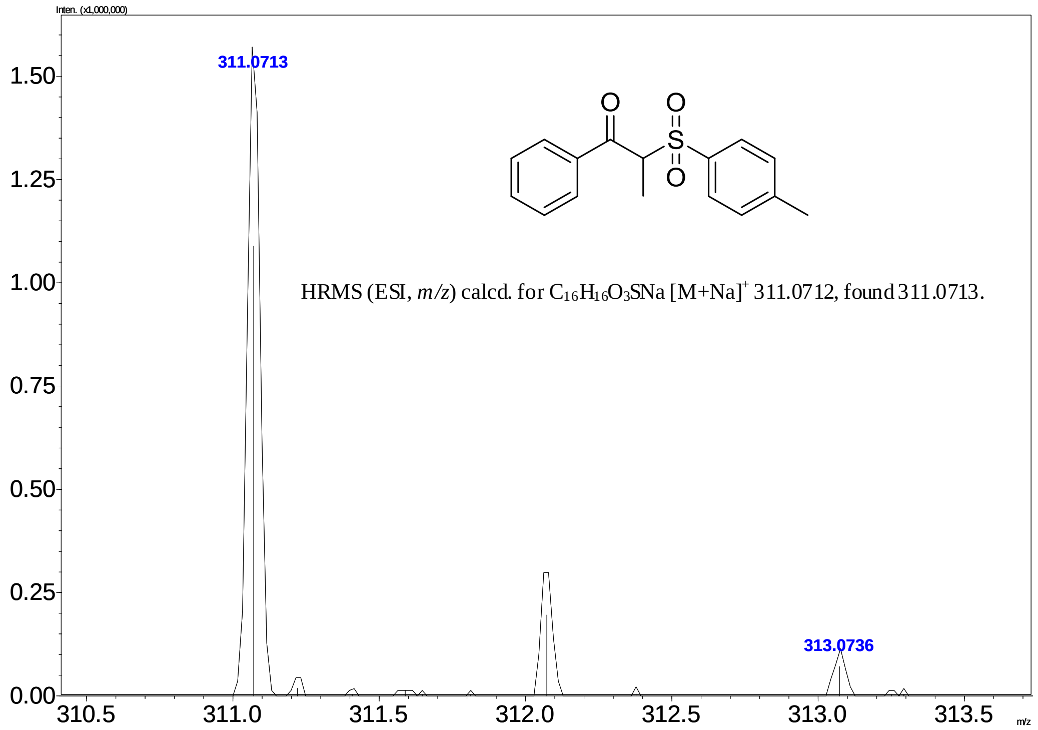

HRMS spectrum of product **3ab**

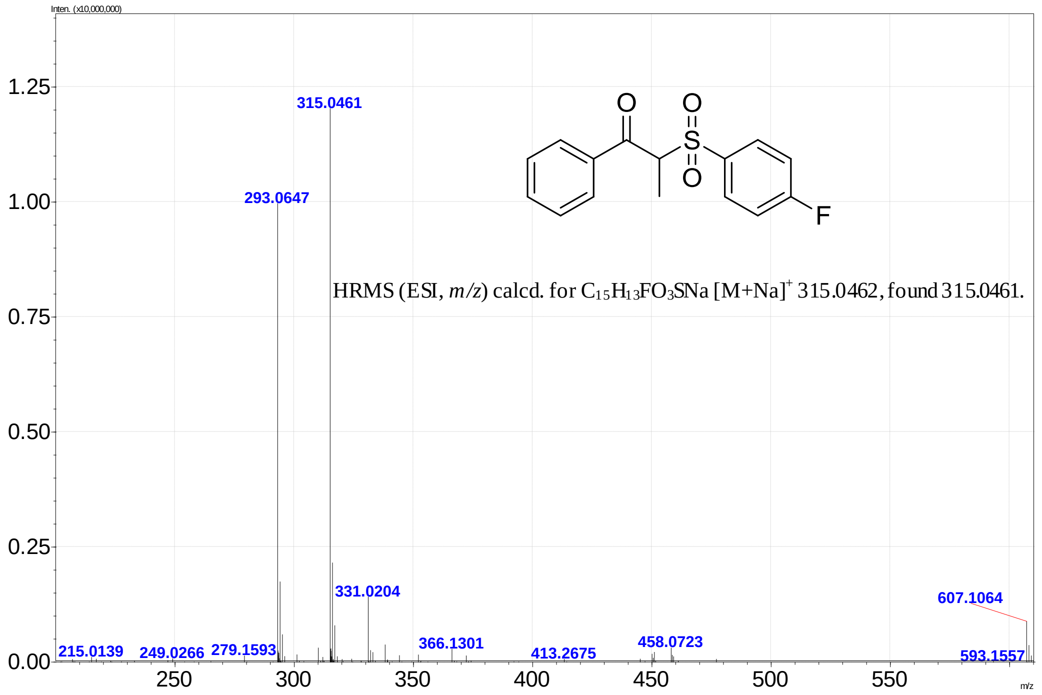

HRMS spectrum of product **3ac**

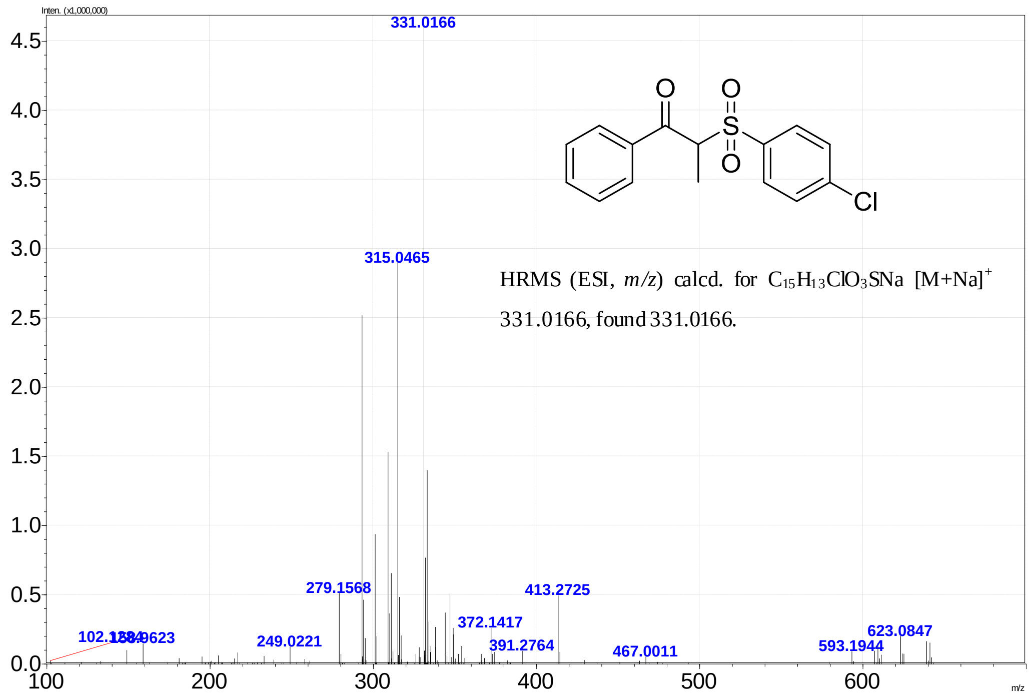

HRMS spectrum of product **3ad**

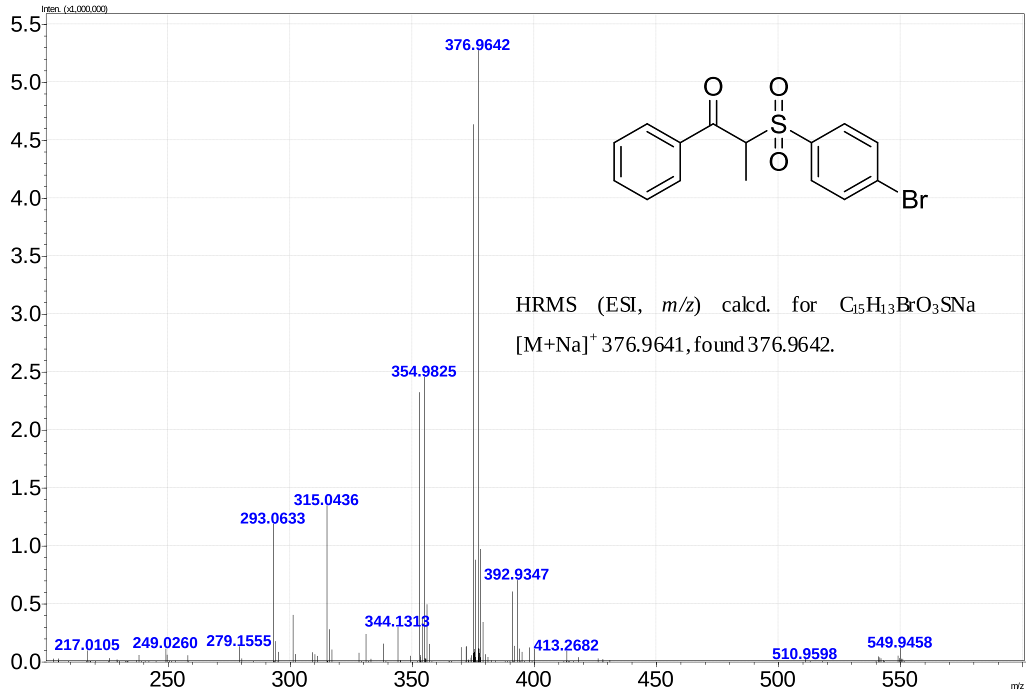

HRMS spectrum of product **3ae**

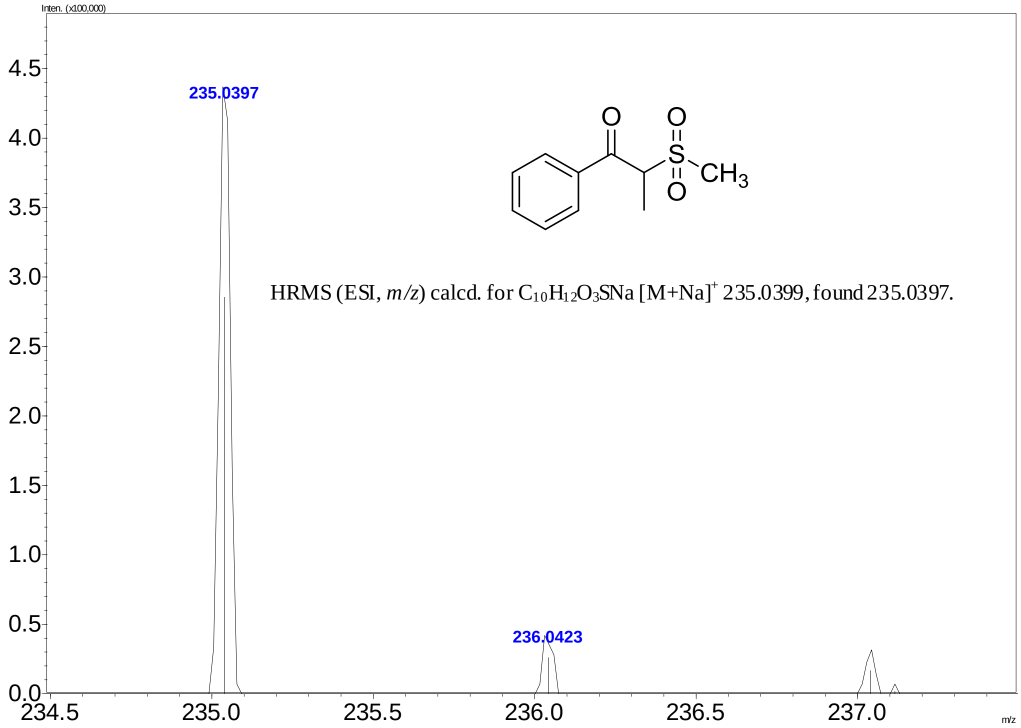

HRMS spectrum of product **3af**

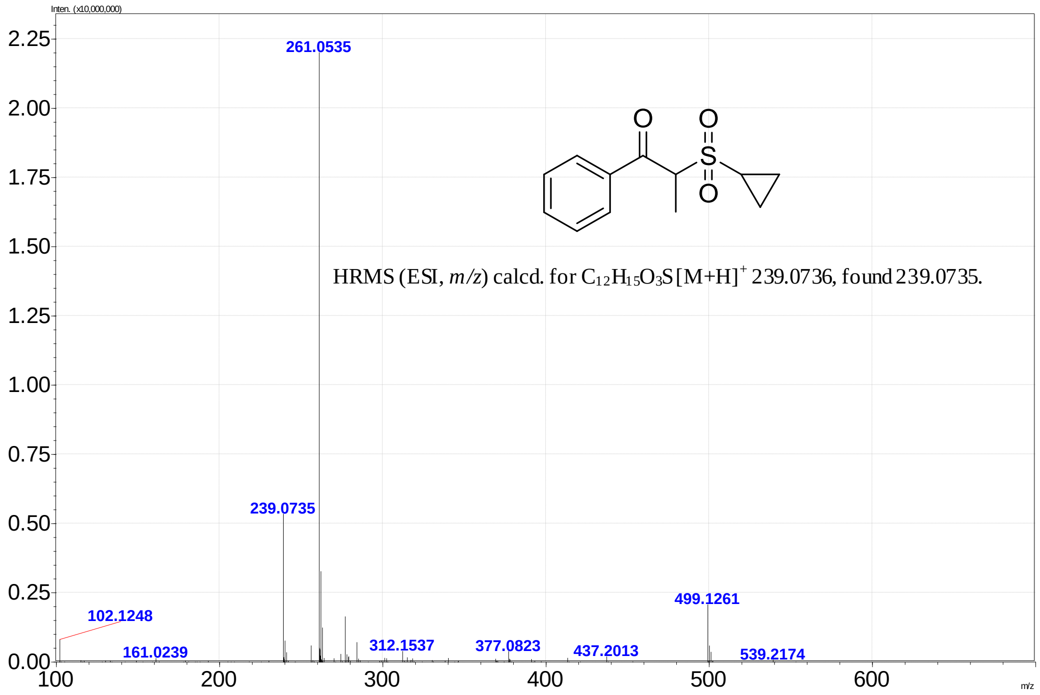

HRMS spectrum of product **3ag**

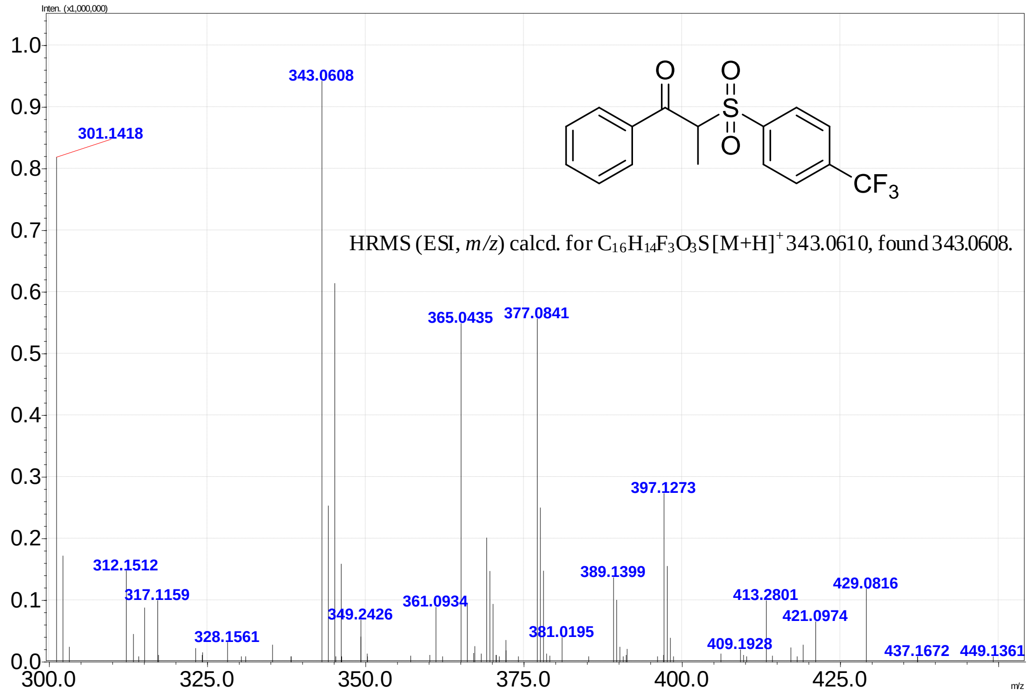

HRMS spectrum of product **3ah**

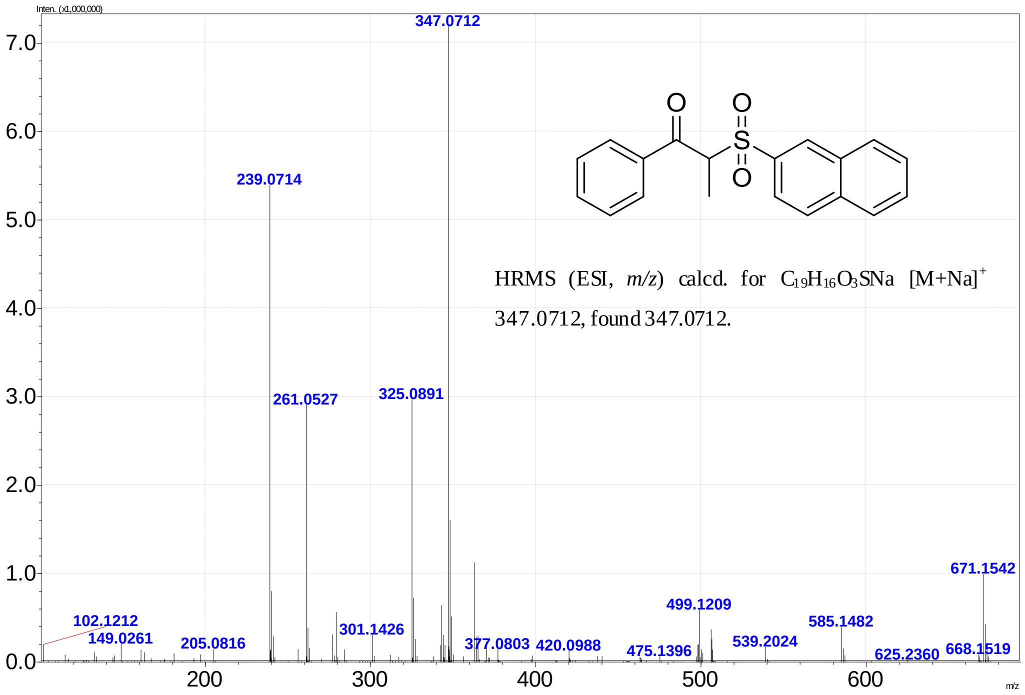

HRMS spectrum of product **3ai**

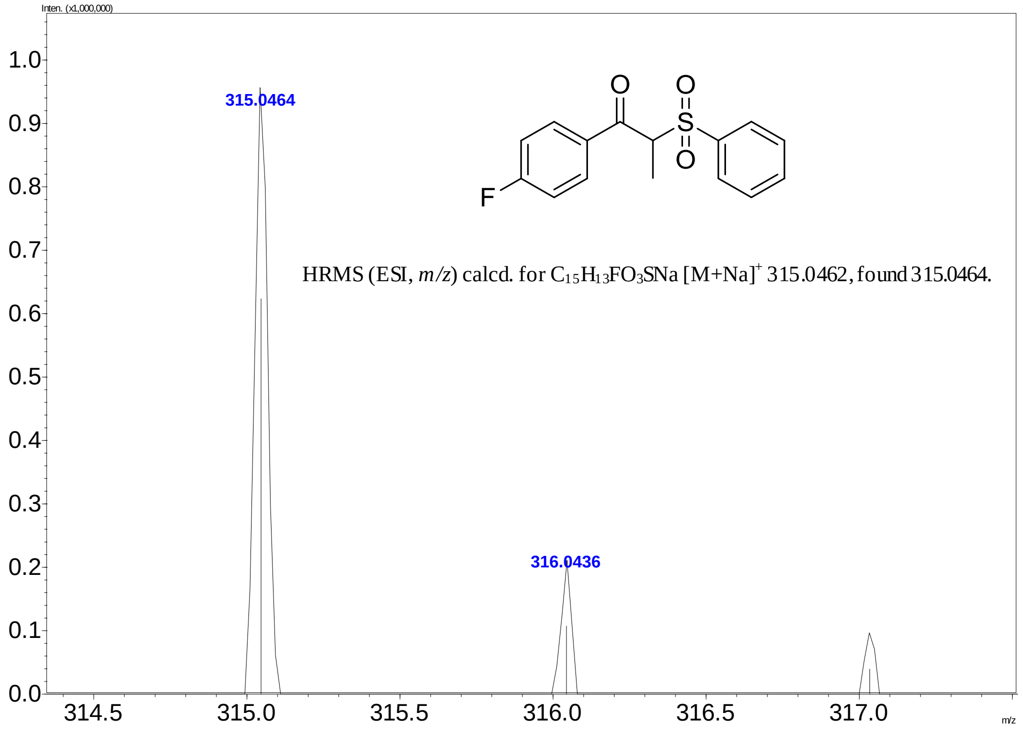

HRMS spectrum of product **3ba**

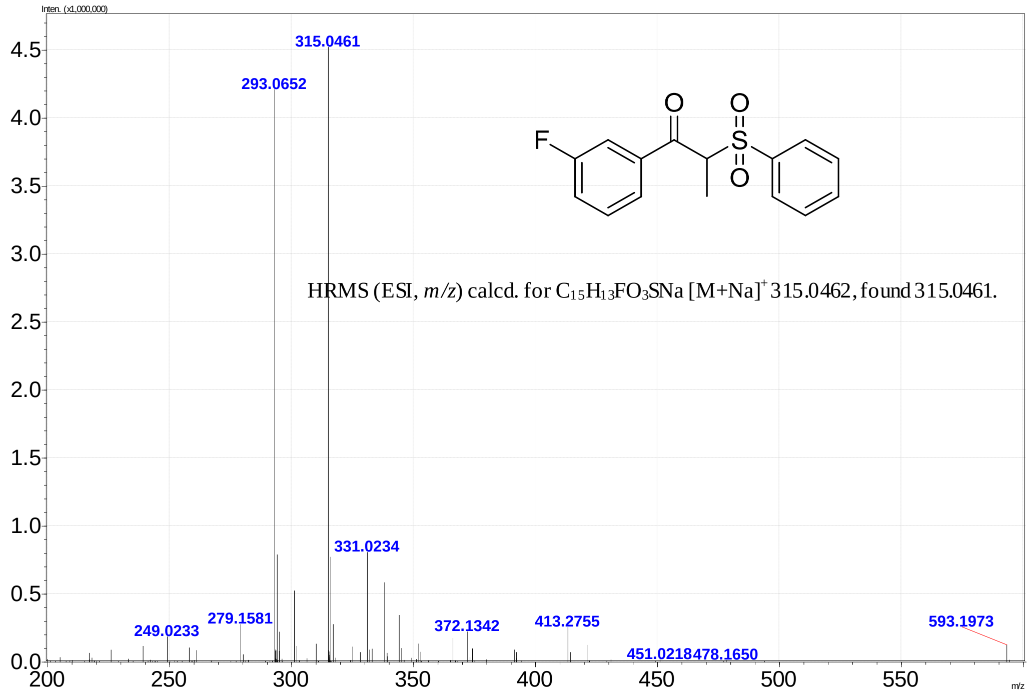

HRMS spectrum of product **3ca**

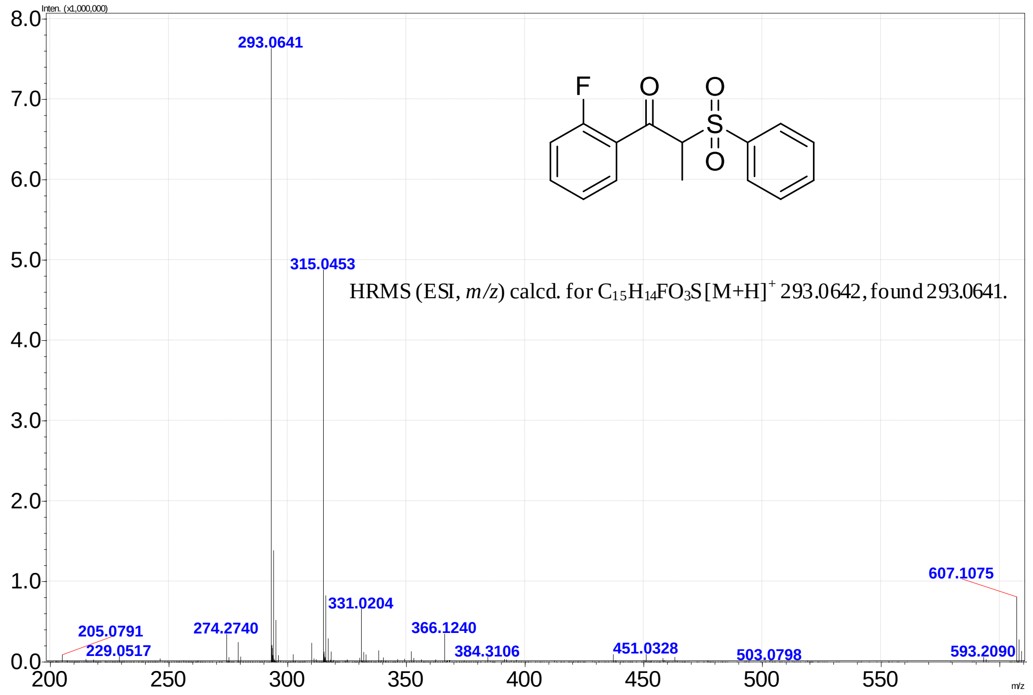

HRMS spectrum of product **3da**

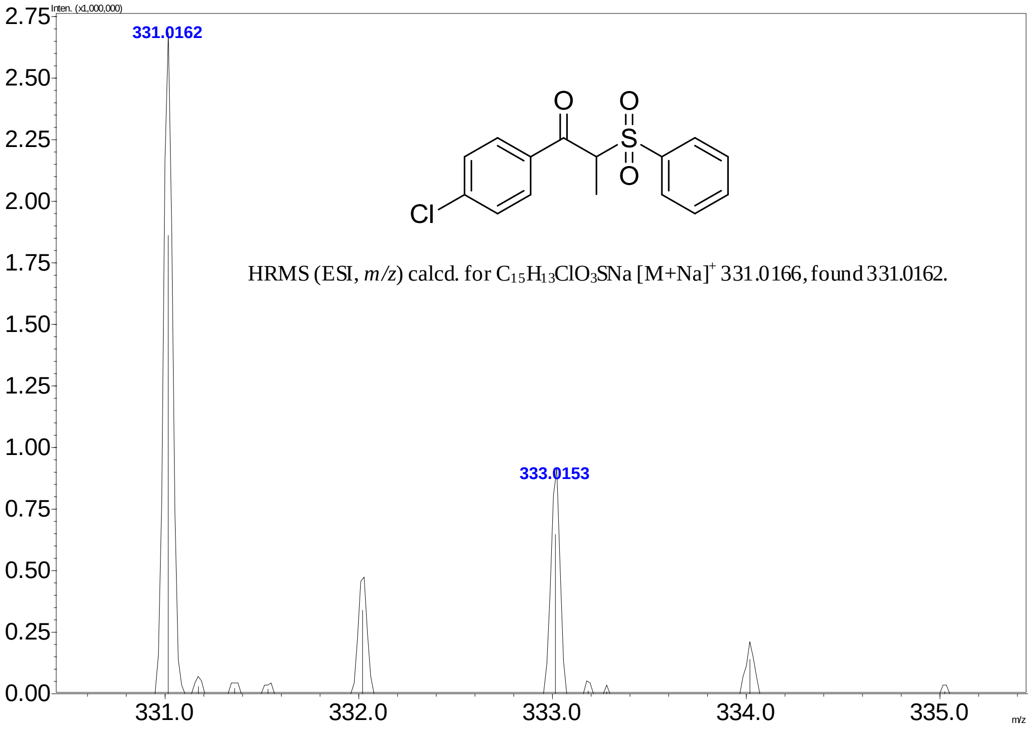

HRMS spectrum of product **3ea**

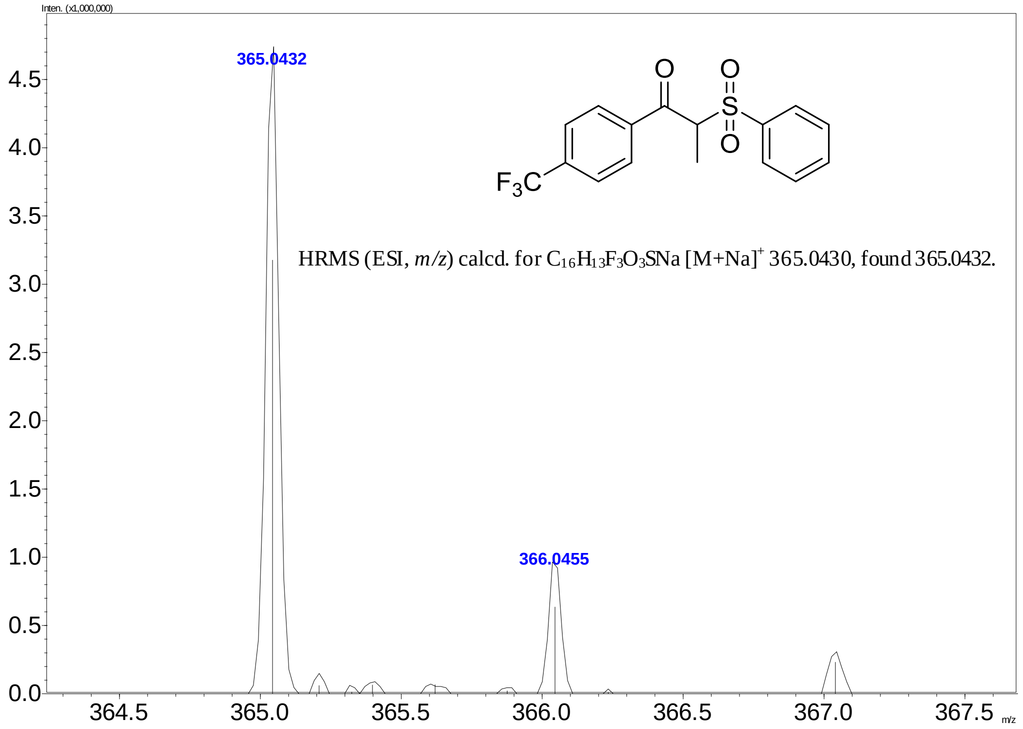

HRMS spectrum of product **3fa**

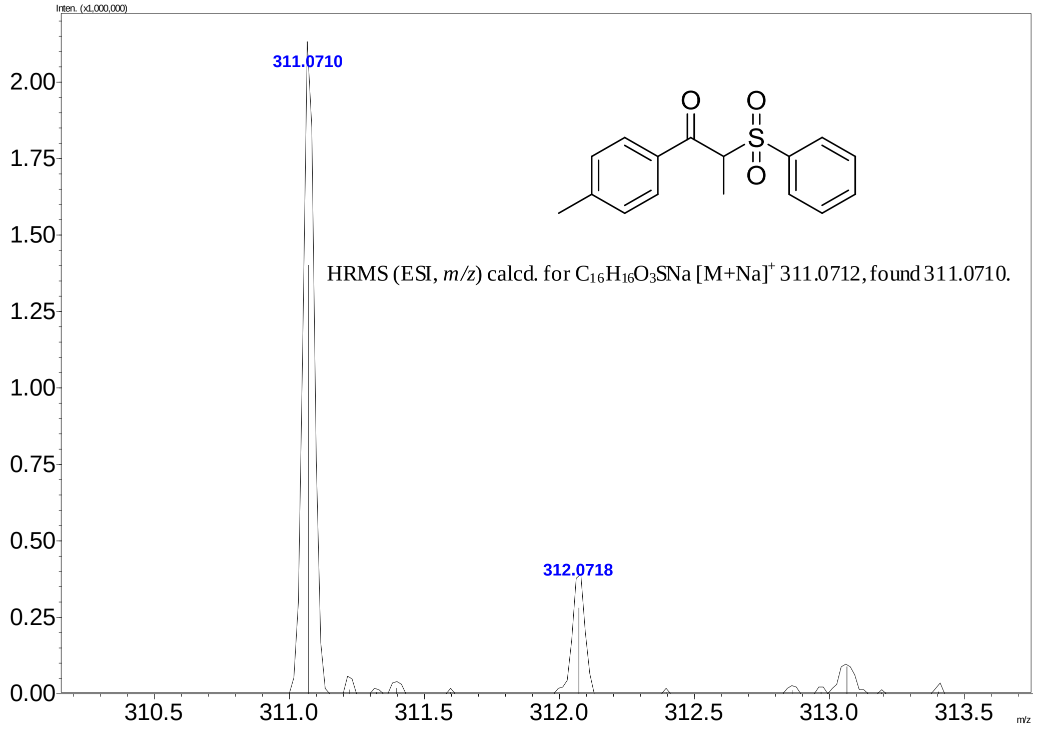

HRMS spectrum of product **3ga**

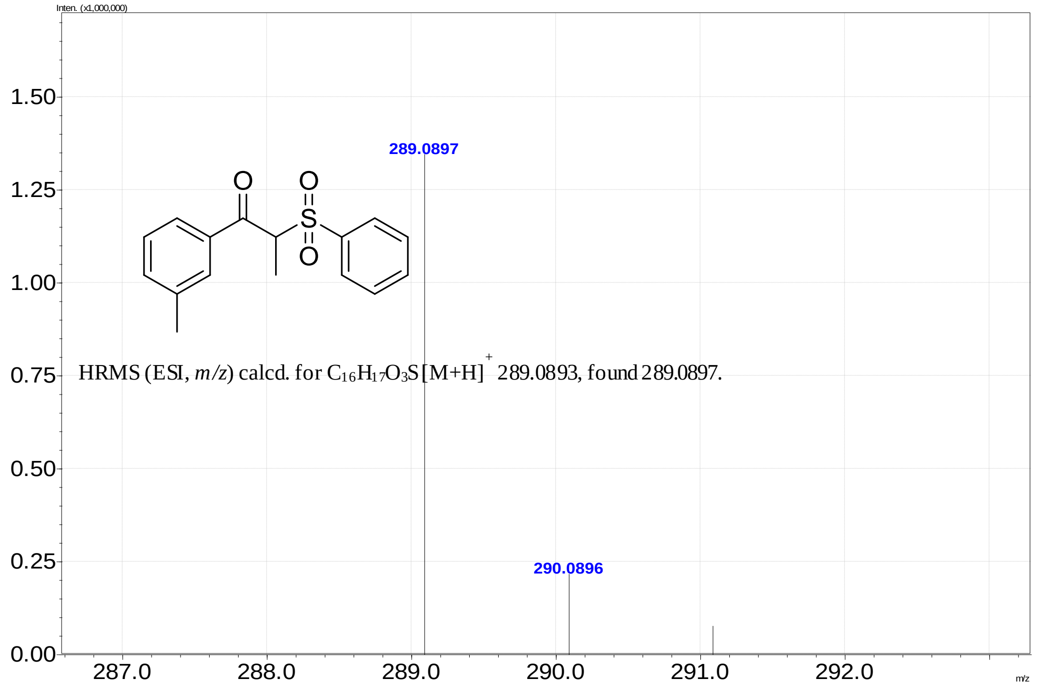

HRMS spectrum of product **3ha**

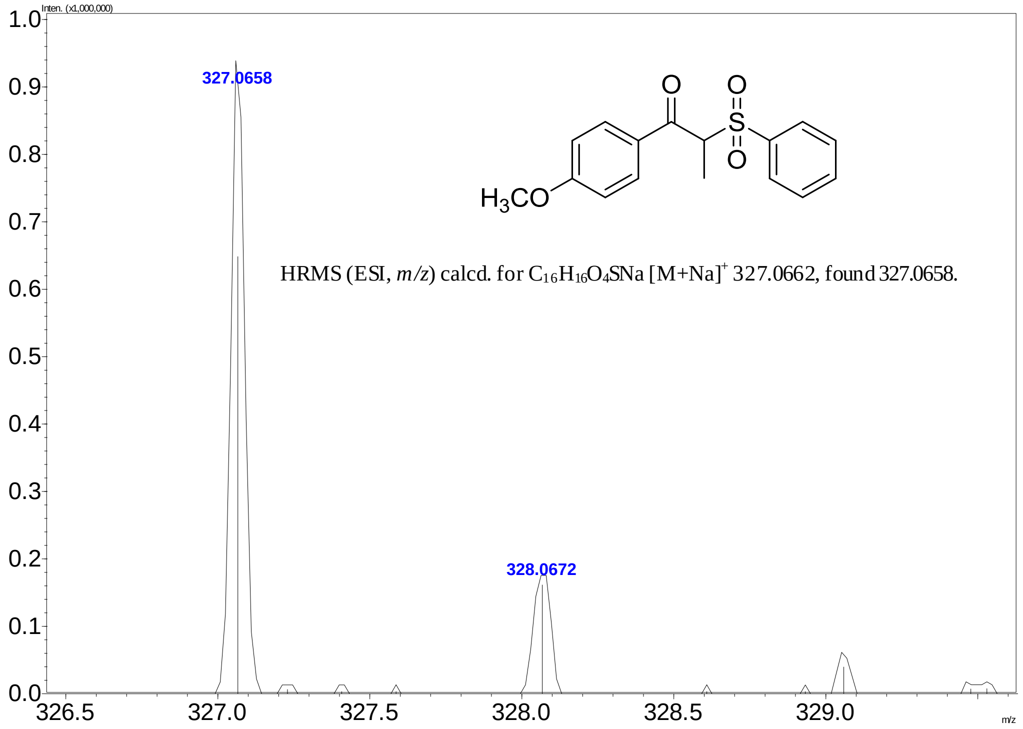

HRMS spectrum of product **3ia**

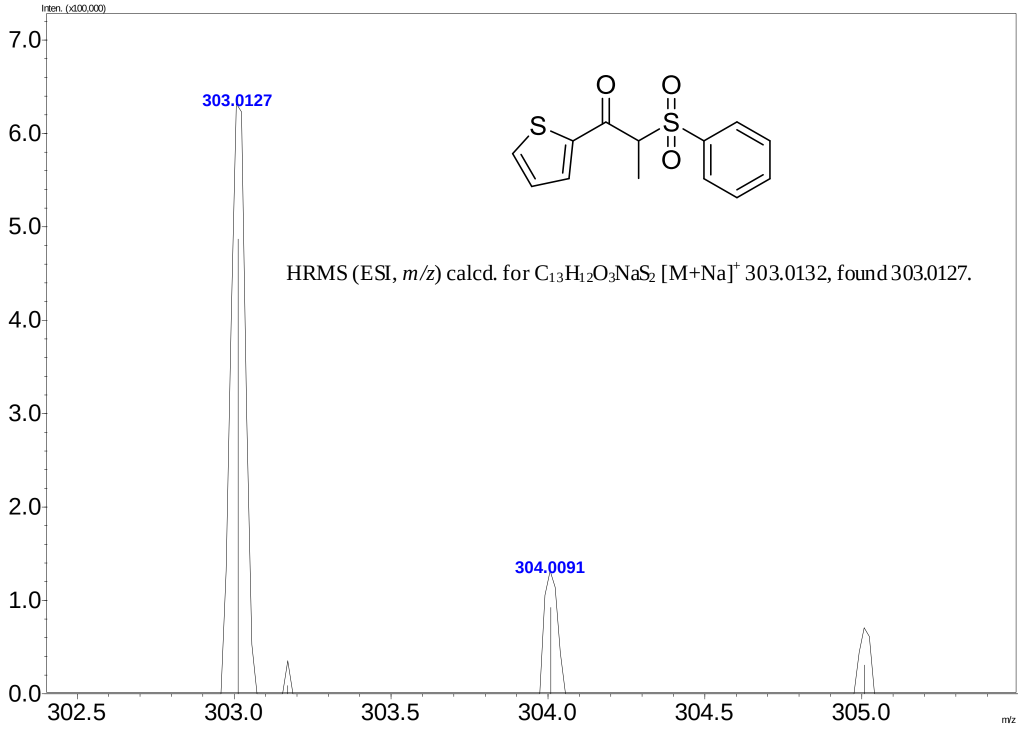

HRMS spectrum of product **3ja**

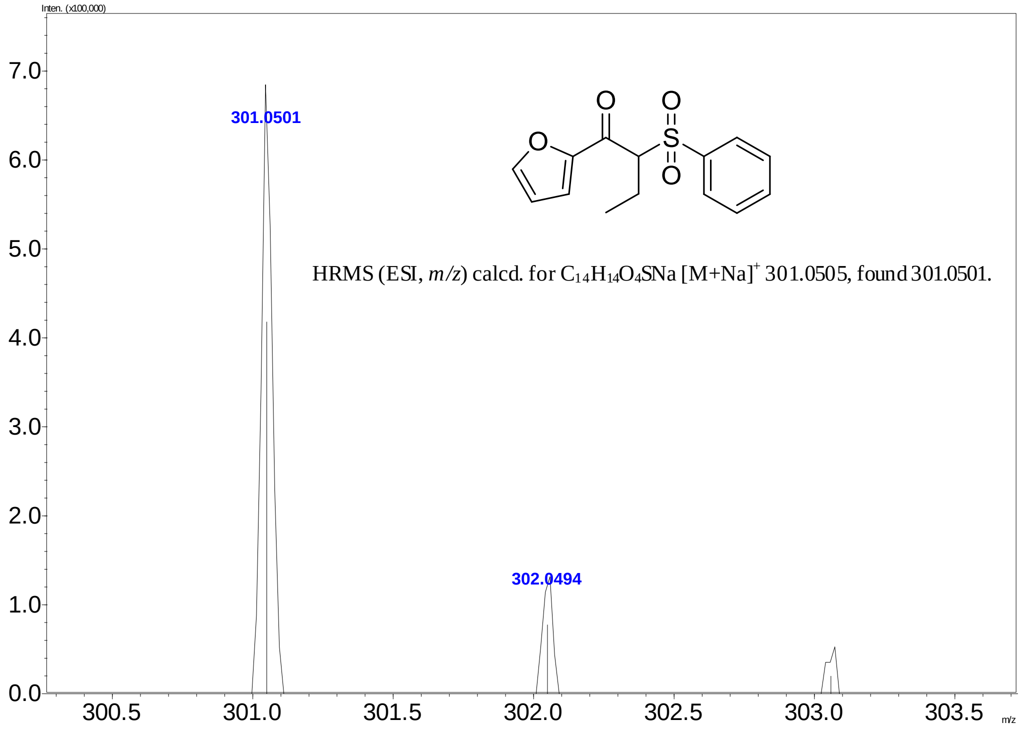

HRMS spectrum of product **3ka**

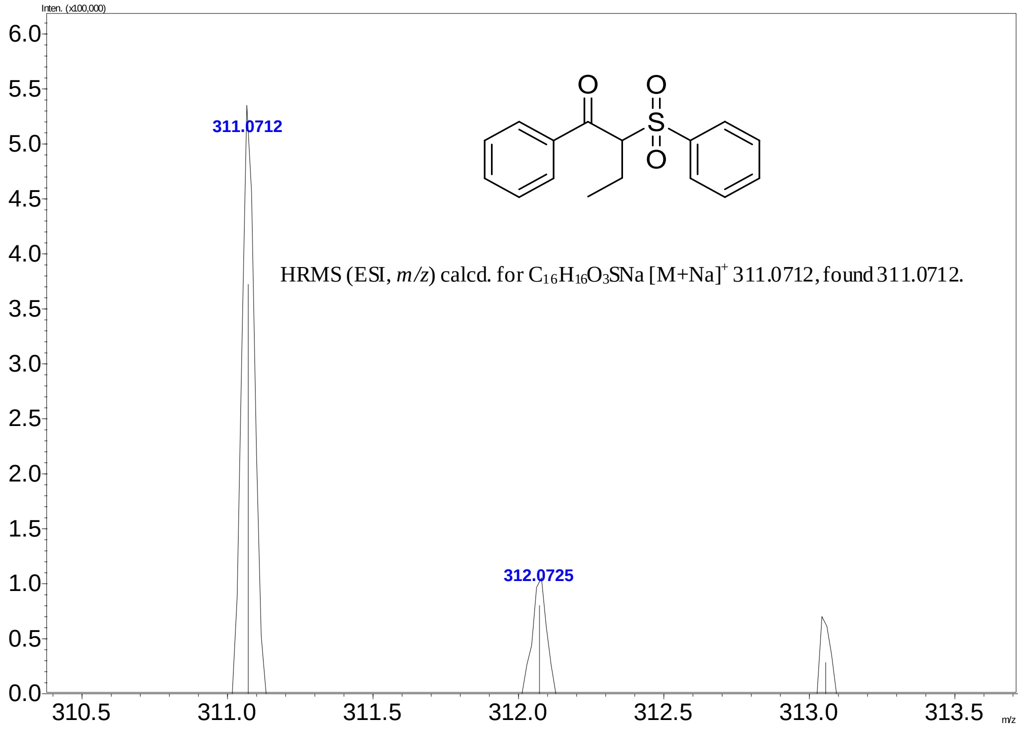

HRMS spectrum of product **3la**

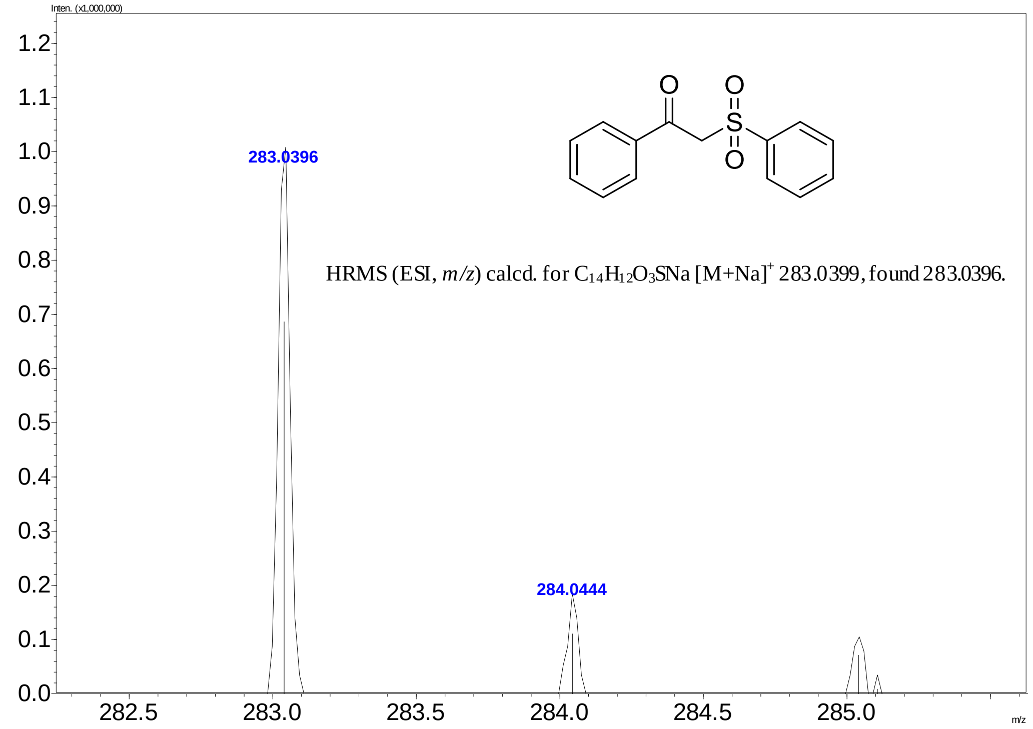

HRMS spectrum of product **3ma**

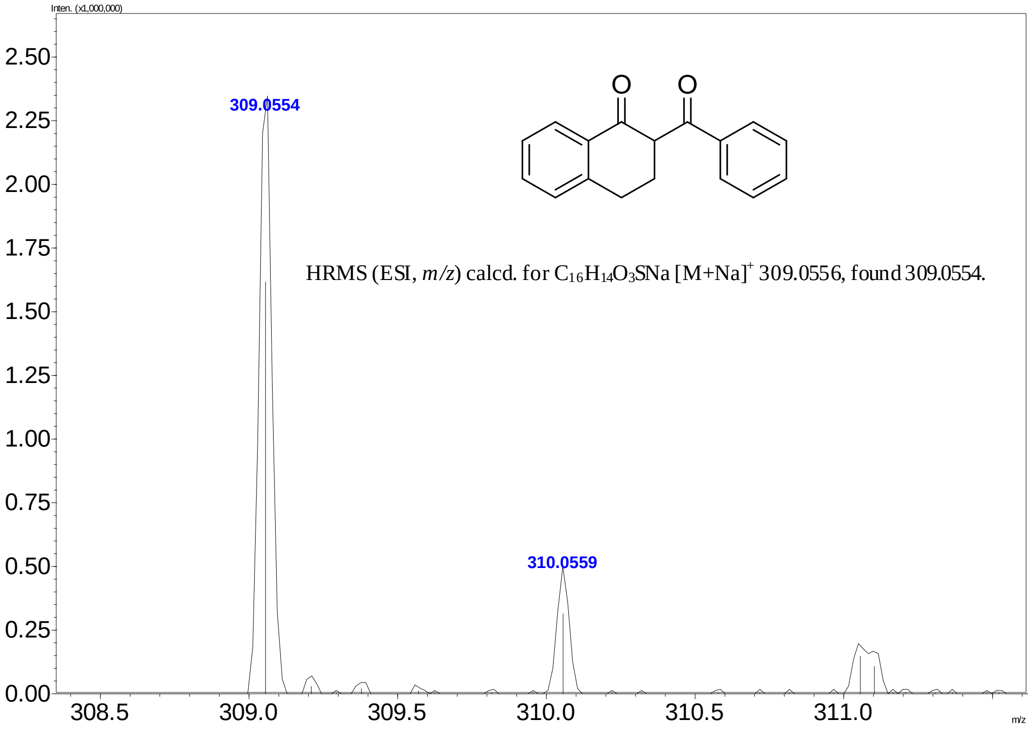

HRMS spectrum of product **3na**

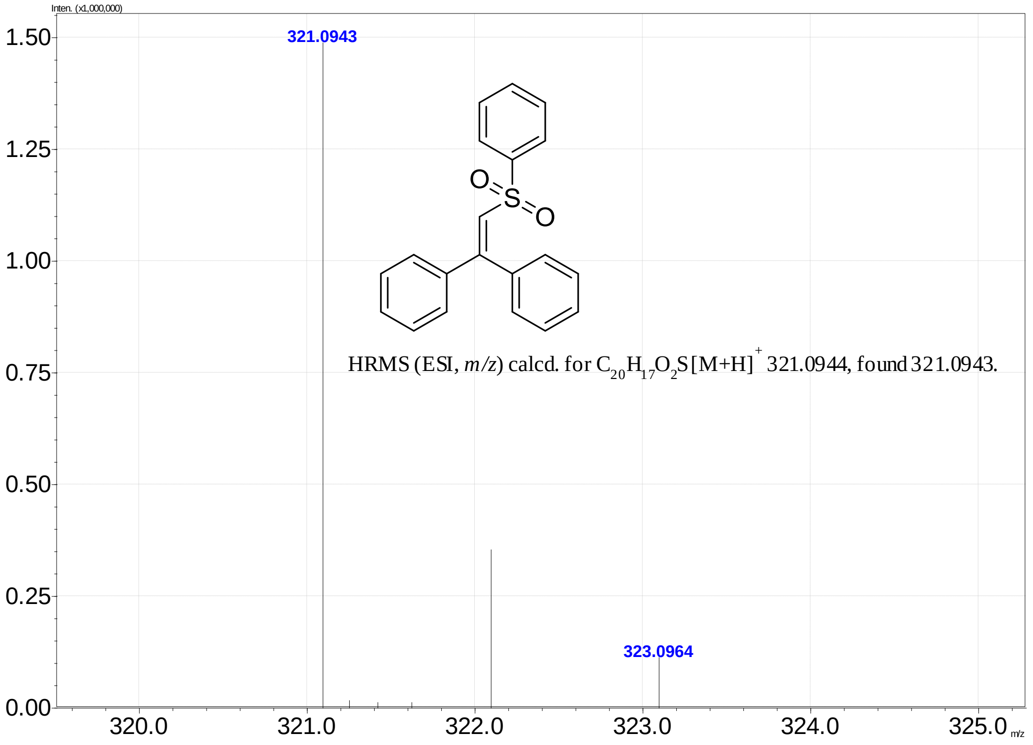

HRMS spectrum of product **4a**

7. Copy of HSQC Spectrum of 3aa

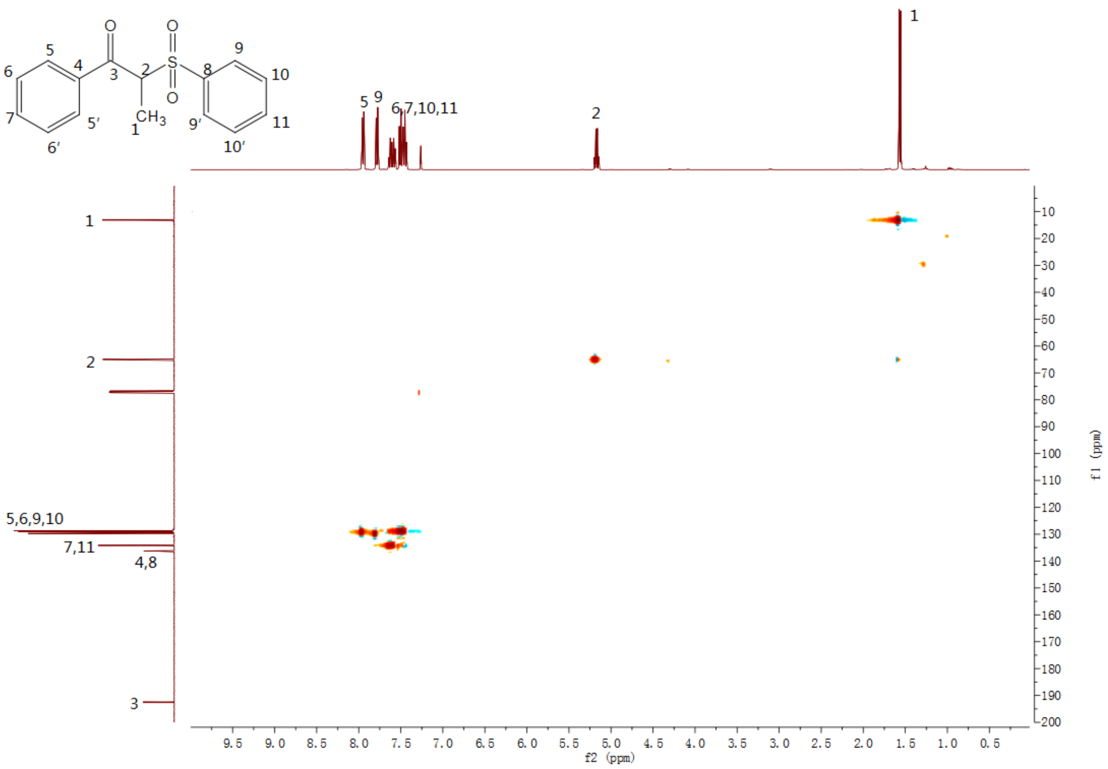

8. Copy of HMBC Spectrum of 3aa

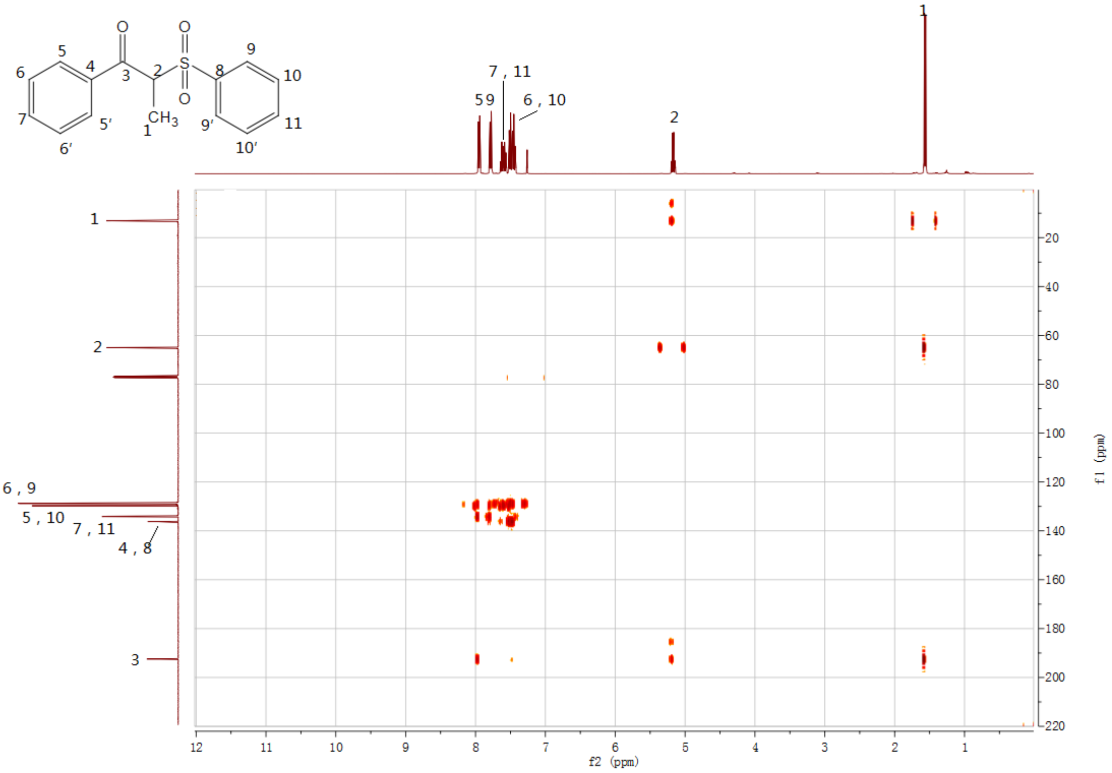

proton \ carbon		C1	C2	C3	C4	C5	C5’	C6	C6’	C7	C8	C9	C9’	C10	C10’	C11
		13.11	64.97	192.45	136.23	129.10	129.10	128.88	128.88	134.03	136,25	128.74	128.74	129.73	129.73	134.18
H1	1.57	bonded	α	β												
H2	5.18	α	bonded	α												
H5	7.94			β	α	bonded	β	α		β						
H5’	7.94			β	α	β	bonded		α	β						
H6	7.60				β	α		bonded	β	α						
H6’	7.60				β		α	β	bonded	α						
H7	7.47					β	β	α	α	bonded						
H9	7.78										α	bonded	β	α		β
H9’	7.78										α	β	bonded		α	β
H10	7.43										β	α		bonded	β	α
H10’	7.43										β		α	β	bonded	α
H11	7.51											β	β	α	α	bonded
